# Development and validation of a LAMP assay for detection of genetically diverse *Lactococcus* c2-type bacteriophages in dairy environments

**DOI:** 10.3389/fmicb.2026.1912103

**Published:** 2026-08-10

**Authors:** Sofia A. Venedyukhina, Ivan D. Antipenko, Ninel P. Sorokina, Kseniya A. Smagina, Andrey B. Lisitsyn, Aleksandr G. Kruchinin, Maxim Yu Shkurnikov

**Affiliations:** 1 Laboratory for Research on Molecular Mechanisms of Longevity, Department of Biology and Biotechnology, HSE University, Moscow, Russia; 2 All-Russian Research Institute of Butter and Cheese Making, Branch of the Gorbatov Federal Research Center for Food Systems, Uglich, Russia; 3 Gorbatov Federal Research Center for Food Systems of RAS, Moscow, Russia

**Keywords:** bacteriophage detection, c2-type bacteriophages, cheese whey, dairy industry, high-fidelity DNA polymerase, *Lactococcus lactis*, LAMP

## Abstract

Bacteriophages infecting *Lactococcus* spp. are a major cause of fermentation failures in the dairy industry. Among them, c2-type phages remain insufficiently studied, and rapid detection methods are limited. This study aimed to develop a loop-mediated isothermal amplification (LAMP) assay for sensitive detection of genetically diverse c2 phages. As part of this work, 12 prevalent c2 phages isolated from Russian dairy plants and 22 additional c2 phages were sequenced and analyzed. Conserved genomic regions were identified, and two primer sets were designed to account for the high genetic variability of c2 phages. Together, these primer sets enabled detection of all 34 tested c2 isolates despite varying numbers of mismatches within primer-binding regions. The assay detected 34 c2 isolates with varying numbers of mismatches in primer-binding regions. The detection limit was 10^2^ copies/μL for plasmid DNA and 10^2^–10^3^ PFU/mL for bacterial lysates. Validation using industrial cheese whey samples confirmed assay applicability. Addition of high-fidelity Tersus polymerase improved mismatch tolerance, and *in silico* analysis predicted potential detection of up to 98% of publicly available c2 phages genomes. The developed assay provides a rapid and cost-effective tool for phage monitoring in dairy fermentations and may help reduce the risk of fermentation failures. Furthermore, the proposed approach may serve as a framework for developing diagnostic assays targeting other genetically diverse bacteriophage groups.

## 1 Introduction

Microbial cultures, predominantly lactic acid bacteria, play a central role in fermentation processes in the production of fermented dairy products (del Rio et al., 2012). One of the major causes of fermentation failures is the infection of starter cultures by bacteriophages (phages) (White et al., 2024). Currently, bacteriophages are considered among the most significant and persistent threats to industrial dairy fermentations, as their wide distribution, high adaptability, and expanding host range contribute to increased frequencies of technological disruptions and associated economic losses (Ortiz Charneco et al., 2023; Ranveer et al., 2024).

The consequences of phage contamination include reduced fermentation efficiency, deterioration of product quality, proliferation of undesirable microbiota, and in severe cases, complete process failure (Moineau and Lévesque, 2004). It has been estimated that bacteriophages account for approximately 0.1–15% of technological failures in fermented dairy production (Szczepankowska et al., 2013).

Members of the genus *Lactococcus* are among the most widely used microorganisms in dairy fermentations, including the production of cheese and yogurt (Romero et al., 2020). Phages infecting *Lactococcus* spp. are classified into ten groups; however, three of them – P335, 936 (*Skunavirus*), and c2 (*Ceduovirus*) – are most frequently encountered in industrial settings (Marcó et al., 2012). Together, these groups account for approximately 98% of all known lactococcal phages (Deveau et al., 2006). Globally, the 936 group is the most prevalent, followed by P335, while c2 phages represent the third most frequently detected group (Mahony et al., 2017; Oliveira et al., 2018). Data on the distribution of lactococcal phages in Russia remain limited. Nevertheless, available studies indicate that phages belonging to the 936 and c2 groups predominate in dairy production environments, with c2 phages in some reports considered among the most widespread (Oliveira et al., 2018; Pechenov et al., 2023).

Effective control of phage populations at different stages of production is essential to minimize phage-associated risks, which has led to the development of numerous detection methods. The classical double-layer agar assay remains a standard approach due to its simplicity; however, it requires prolonged incubation, limiting its applicability in the processing of perishable raw materials such as milk (del Rio et al., 2012). More recently, this method has been increasingly replaced by faster and more sensitive molecular approaches, particularly PCR. PCR enables the detection of bacteriophage DNA in various sample types within 2–4 h with high sensitivity and specificity (White et al., 2022). However, its application is limited by the need for specialized and relatively expensive equipment, as well as more complex sample preparation procedures.

An alternative approach suitable for field and industrial applications is loop-mediated isothermal amplification (LAMP). Unlike PCR, LAMP does not require sophisticated instrumentation and can be performed using simple equipment such as a water bath or heating block (Cui et al., 2024). Results can be obtained within 15–60 min, significantly reducing assay time (Bu et al., 2022). In addition, LAMP is generally more tolerant to inhibitory substances present in complex food matrices, facilitating its application in dairy production environments (Kumar and Mondal, 2015).

In the dairy industry, LAMP has been widely applied for the detection of microbial contamination in milk; however, relatively few studies have focused on bacteriophage detection. One of the few reported examples is the LAMP-based detection of phage P680, belonging to the 936 group and infecting *Lactococcus* spp., in water and whey samples (Brinks et al., 2018). Although c2 phages are less prevalent than 936 phages, they remain a significant threat to dairy fermentation processes. Moreover, c2 phages are considerably less studied compared to the 936 and P335 groups (Chmielewska-Jeznach et al., 2020), and no rapid and reliable LAMP-based detection systems are currently available for this phage group. Therefore, the development of such assays is highly relevant, as LAMP-based detection would enable rapid and cost-effective monitoring of c2 phages in industrial samples, thereby reducing the risk of fermentation failures and associated economic losses.

When developing diagnostic assays, it is essential to define an appropriate detection limit that ensures both analytical performance and practical applicability for industrial monitoring. Critical phage concentrations in dairy raw materials vary depending on the phage type, the phage resistance of *Lactococcus* strains, and processing conditions. Nevertheless, levels in the range of 10^4^–10^5^ PFU/mL are often considered threshold values, as further increases can rapidly lead to phage propagation and lysis of starter cultures, even those with high phage resistance (Ganina, 2022). Concentrations of 10^2^–10^4^ PFU/mL may already cause fermentation disturbances, whereas titers of 10^6^–10^7^ PFU/mL are typically associated with severe technological failures (Moineau and Lévesque, 2004; Pujato et al., 2019). In particular, it has been shown that at an initial c2 phage concentration of approximately 3 × 10^3^ PFU/mL (MOI = 10^−4^), massive cell lysis begins within approximately 3 h post-infection, reaching titers of ~10^9^ PFU/mL (Michelsen et al., 2007). Based on these observations, a detection limit of no higher than 10^3^ PFU/mL is considered appropriate, as it would enable early detection of contamination 2–3 h prior to the onset of massive lysis, allowing timely implementation of corrective measures. An additional challenge is the high genomic variability of bacteriophages (Li et al., 2019), which necessitates the selection of conserved genomic regions for the development of robust diagnostic assays.

**Figure 1:**
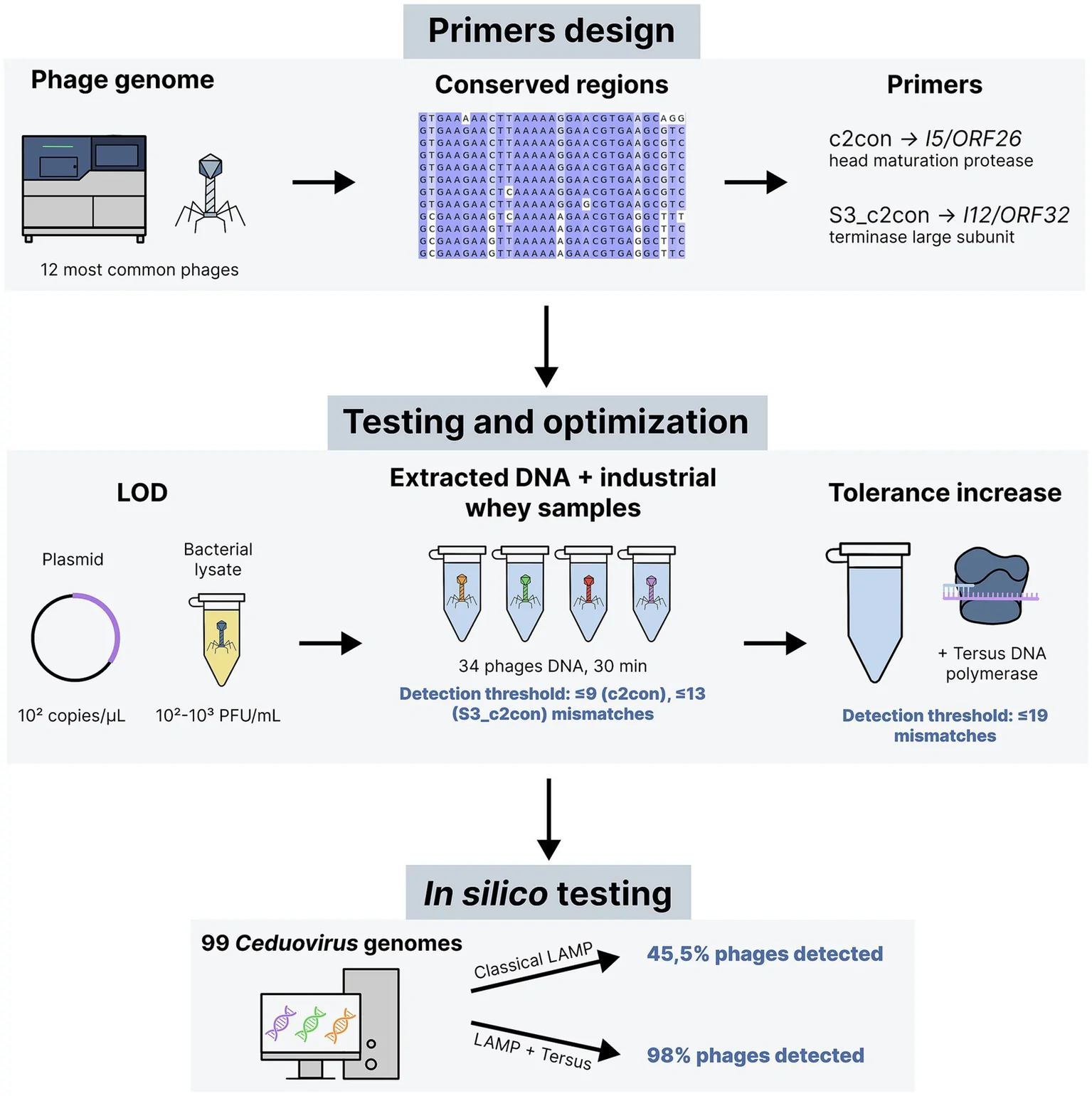
Study workflow for the development and validation of the LAMP assay for detection of genetically diverse c2-type bacteriophages.

The aim of this study was to develop a sensitive LAMP-based assay for the detection of c2 bacteriophages that is tolerant to their genetic variability, based on the analysis of conserved genomic regions and subsequent validation on a panel of industrial isolates. The overall study workflow is summarized in Figure 1.

## 2 Materials and methods

### 2.1 Phage strains

c2-type bacteriophages from the Collection of Lactic Acid Bacteria for Cheese Production and Their Bacteriophages (All-Russian Dairy Research Institute, Branch of the V. M. Gorbatov Federal Research Center for Food Systems of RAS) were used in this study. Phages were isolated from cheese whey samples. The sensitive host strain *Lactococcus lactis* subsp. *lactis* was cultivated in M17 broth supplemented with lactose (HiMedia, India). Bacteriophages were isolated using the double-layer agar method as previously described (Chuksina et al., 2024). Bacterial lysates containing phage particles were stored at −80 °C until use.

### 2.2 Extraction of phage DNA from bacterial lysates

Prior to phage precipitation, bacterial lysates were filtered through 0.22 μm membrane filters. Polyethylene glycol PEG6000 (neoFroxx, Cat. No. 1518KG001) and NaCl were added to the filtrates to final concentrations of 4% and 1 M, respectively. Samples were incubated overnight at 4 °C and centrifuged at 15,000 × g for 15 min at 4 °C. The resulting pellets were resuspended in distilled water to a final volume of 200 μL. DNase I (Qiagen, Cat. No. 79254; 1,500 Kunitz units, 12.5 μL/mL) was added to the suspensions followed by incubation at 37 °C for 30 min. DNase was inactivated by incubation at 75 °C for 20 min. Subsequently, 1.25 μL of proteinase K solution (20 mg/mL) was added and samples were incubated at 56 °C for 1 h. DNA extraction was performed using the ExtractDNA Blood and Cells kit (Evrogen, Russia) according to the manufacturer’s instructions.

### 2.3 Genome sequencing and assembly

DNA libraries were prepared using the MGIEasy Fast FS DNA Library Prep Set V2.0 (MGI, China). Library quality was assessed using the Qubit 1X dsDNA HS Assay Kit (Thermo Fisher Scientific, USA) and fragment size distribution was analyzed by capillary electrophoresis using a QIAxcel Advanced system with the QX DNA Fast Analysis Kit (Qiagen, Germany). Sequencing was performed on the MGI DNBSEQ-G50 platform (BGI, China) in paired-end 150 bp mode (PE150). Raw read quality was assessed using FastQC v0.12.1 and preprocessing was performed with fastp v0.23.2. Taxonomic classification was conducted using Kraken2 (Wood et al., 2019) with the standard database (accessed on 31 October 2024), genome assembly using SPAdes v4.0.0, assembly quality assessment using QUAST v5.2.0, and viral genome validation using CheckV v1.0.1. Preliminary phage identification was performed using BLAST.

Whole-genome sequences were deposited in GenBank under accession numbers shown in Supplementary Table S1.

### 2.4 LAMP primer design

To identify conserved genomic regions suitable for LAMP primer design, multiple sequence alignment of phage genomes was performed using Clustal Omega (Sievers and Higgins, 2014) with the parameters --force --threads = 2 --verbose. Conserved regions of approximately 800 and 600 nucleotides were identified based on alignment conservation scores. Conservation was assessed for each alignment position by calculating the frequency of the most abundant nucleotide. Fully conserved positions contributed maximally to the cumulative conservation score, whereas variable positions contributed proportionally less. A sliding-window approach was subsequently applied to identify genomic regions with the highest cumulative conservation. LAMP primers were designed using the NEB LAMP Primer Design Tool (primer set c2con) and PrimerExplorer V5 (primer set S3_c2con).

### 2.5 Plasmid standard preparation

Fragments corresponding to the head maturation protease gene (*l5*) of phage 309A and the terminase large subunit gene (*ORF32*) of phage 65 were amplified using Q5 High-Fidelity DNA Polymerase (NEB, USA). Amplicons were cloned into the pX458 vector and transformed into *Escherichia coli* JM109 competent cells. Plasmids were purified using the Plasmid MiniPrep 2.0 kit (Evrogen, Russia) according to the manufacturer’s instructions. Plasmid concentration and purity were measured spectrophotometrically using a NanoDrop instrument.

### 2.6 In silico analysis of LAMP primers

An *in silico* analysis was performed to evaluate the applicability of the developed primer sets to genetically diverse c2 phage isolates. For this purpose, a custom Python v3.12 script was developed using the Biopython library (SeqIO and Seq modules) to screen each phage genome for potential primer-binding sites. For each primer, the reverse-complement sequence was calculated where required, and the script performed a sliding-window comparison of the primer sequence against the full length of each genome, evaluating every possible alignment position. At each position, the number of mismatches between the primer and the corresponding genomic fragment was counted. Mismatch analysis was performed using both c2 phage genomes obtained in this study and 99 publicly available complete c2 phage genomes, as well as genomes representing other Lactococcus phage groups, including the 936 group (*n* = 214), P335 group (*n* = 38), and the 1,358, Q54, P087, 1706, 949, P034, and KSY1 groups (*n* = 68 genomes combined). All complete genome sequences were retrieved from the NCBI GenBank database (accessed on 09 July 2026). Comparative whole-genome visualization of the investigated bacteriophage genomes was performed using clinker v0.0.32 (Gilchrist and Chooi, 2021).

### 2.7 LAMP assay conditions and determination of the limit of detection

Real-time LAMP amplification with SYBR Green I detection was performed using the BioMaster LAMP SYBR 2 × kit (Biolabmix, Russia), which contains *Bst* DNA polymerase. Each 25 μL reaction mixture contained 2.5 μL of DNA template. Lambda phage genomic DNA (1.88 × 10^10^ PFU/mL) or nuclease-free water was used as a negative control. Amplification was carried out at 65 °C for 60 min using a DTprime thermocycler (DNA-Technology, Russia).

DNA of phages 309A and 65 used in the optimization experiments was extracted from bacterial lysates obtained after phage propagation on host bacterial cultures. Primer concentration optimization was performed using phage 65 DNA at 26.4 pg./μL (10^9^ PFU/mL). Temperature optimization for the c2con primer set was performed using phage 309A DNA at 5.3 pg./μL (2 × 10^8^ PFU/mL), whereas optimization of the S3_c2con set used phage 65 DNA at 28.2 pg./μL (10^9^ PFU/mL). Qualitative detection experiments were conducted using phage DNA at 0.01 ng/μL (4 × 10^8^ PFU/mL).

The analytical sensitivity of the assay was evaluated using 10-fold serial dilutions of plasmid standards ranging from 10^6^ to 10^−1^ copies/μL. For testing with biological phage-containing material, bacterial lysates obtained after propagation of phages 309A and 65 on host bacterial cultures were used as a source of native phage particles and diluted from 10^8^ to 10^−1^ PFU/mL. Prior to amplification, lysates were heated at 95 °C for 10 min. The LOD was defined as the lowest target concentration producing positive amplification in at least 95% of reactions (Anderson, 1989).

Comparability between amplification kinetics in plasmid standards and bacterial lysates was evaluated using time-to-positive values obtained for corresponding serial dilutions. Correlation was assessed using Pearson’s correlation coefficient with two-sided *p*-values.

Alternative endpoint detection approaches were evaluated using representative c2 phage DNA samples. Fluorescence detection under UV illumination and turbidity-based detection were tested using the BioMaster LAMP SYBR 2 × kit (Biolabmix, Russia). Reactions were performed with phage 309A DNA at a concentration of 5.3 pg./μL and the c2con primer set, or with phage 65 DNA at a concentration of 28.2 pg./μL and the S3_c2con primer set. Colorimetric detection was assessed using the BioMaster LAMP-Color 2 × kit (Biolabmix, Russia) with phage 309A DNA at a concentration of 5.3 pg./μL and the c2con primer set, or with phage 65 DNA at a concentration of 28.2 pg./μL and the S3_c2con primer set. Amplification was carried out at 65 °C for 60 min using a C1000 Touch Thermal Cycler (Bio-rad, USA).

### 2.8 Quantification of encapsidated and free phage DNA

To estimate the proportions of free and encapsidated phage DNA, bacterial lysates were treated with DNase I to selectively remove extracellular DNA. DNase I (Qiagen, Cat. No. 79254; 1,500 Kunitz units, 12.5 μL/mL) was added and samples were incubated at 37 °C for 30 min, followed by enzyme inactivation at 75 °C for 20 min. DNA was subsequently extracted using the ExtractDNA Blood and Cells kit (Evrogen, Russia). Phage DNA quantification was performed by real-time quantitative PCR using phage-specific primer pairs C2 and PC2 (Table 1). Relative changes in DNA content following DNase treatment were calculated based on ΔCt values corrected for amplification efficiency. DNase-resistant DNA was interpreted as the capsid-associated fraction and calculated as 1/E^ΔCt^.

**Table 1:** Primers used to compare the amounts of free and capsid phage DNA.

Primer name	Primer sequence (5′-3′)	Efficiency ± std	Source
C2_Forward	GCATTAAAGCCAACTGATAGC	1.76 ± 0.08	Evseev et al. (2020)
C2_Reverse	AGTAAGAGGGATAGCGAACC		
PC2_Forward	ATGCACGTAAGTCAGGTATC	1.86 ± 0.02	This study
PC2_Reverse	AGACAATGCACCTGAATCAT		

### 2.9 Evaluation of assay specificity

Assay specificity was evaluated using lambda phage genomic DNA (Sibenzyme; 1.88 × 10^10^ PFU/mL), *Lactococcus lactis* subsp. *lactis* genomic DNA (strain 637-4; 0.01 ng/μL), and suspensions obtained from overnight cultures of two industrial *Lactococcus* strains (997-50 and 551-3). Prior to analysis, bacterial suspensions were heated at 95 °C for 20 min. Amplification detected within 30 min was considered a positive result.

Cross-reactivity was additionally assessed *in silico* using 214 complete genomes of 936-group phages, 38 complete genomes of P335-group phages, and 68 complete genomes representing the 1,358, Q54, P087, 1706, 949, P034, and KSY1 groups. Complete genome sequences were retrieved from the NCBI GenBank database (accessed on 09 July 2026) using group-specific phage names as search terms. Genome assemblies were screened for primer-binding sites with no more than six mismatches per primer. In the absence of such sites, successful amplification was considered unlikely.

### 2.10 Improvement of LAMP mismatch tolerance using a high-fidelity DNA polymerase

To increase assay tolerance to primer-template mismatches, a high-fidelity DNA polymerase possessing 3′ → 5′ exonuclease activity was added to the LAMP reaction mixture (Li et al., 2019) as a second polymerase, while *Bst* DNA polymerase remained present as part of the commercial LAMP reagent. Tersus polymerase (Evrogen, Russia), a mixture of thermostable DNA polymerases designed for high-fidelity amplification, was used in this study. Prior to use, the Tersus polymerase solution was pre-heated at 95 °C for 3 min, after which the polymerase was added to the reaction wells. Each reaction contained 0.25 μL of 50x Tersus enzyme and 2.5 μL of 10x Tersus Plus buffer. Phage lysates of isolates 14A, 6A, 296, 311A, 304, 303, 318, 290, 283, and 46 (all at 4 × 10^8^ PFU/mL) were used as templates.

### 2.11 Statistical analysis

The effect of primer-template mismatches on amplification efficiency was evaluated using sequential threshold analysis. Reaction outcomes were analyzed as binary variables (amplification/no amplification), with a positive threshold defined as amplification within 30 min. Effect size was estimated using odds ratios. When zero values were present in contingency tables, the Haldane-Anscombe correction (0.5) was applied. A threshold value was considered significant at *p* < 0.05 and odds ratio > 1.

### 2.12 Gel electrophoresis

LAMP products were analyzed by electrophoresis in 2% agarose gel. Five microliters of amplified DNA mixed with loading dye (4x Gel Loading Dye Blue, Evrogen, Russia) were loaded into each well. DNA fragment size was estimated using a 100 bp DNA ladder (Evrogen, Russia). Electrophoresis was performed at 90 V for 90 min.

### 2.13 Validation on industrial samples

To evaluate assay applicability under industrial conditions, five whey samples obtained from dairy plants were analyzed. Phage titers were determined using the double-layer agar method with a sensitive indicator strain. Phage concentrations in the analyzed samples were 2 × 10^7^, 1 × 10^6^, and 7 × 10^6^ PFU/mL, respectively.

## 3 Results

### 3.1 Genome analysis and identification of conserved regions

Whole-genome sequencing yielded 34 bacteriophage genomes belonging to the c2 group. General genome characteristics are summarized in Supplementary Table S1. Genome sequences of 12 bacteriophages representing the most prevalent c2 isolates detected at Russian dairy plants were used for LAMP primer design. The remaining 22 genomes were subsequently employed for *in silico* validation of the developed primer sets.

Comparative genomic analysis demonstrated that the most conserved regions corresponded to the genes encoding the terminase large subunit (*l12/ORF32*) and the head maturation protease (*l5/ORF26*). Corresponding multiple sequence alignments are presented in Supplementary Figures S1, S2, while a whole-genome comparison of all investigated genomes is shown in Supplementary Figure S3.

### 3.2 LAMP primer sets

Two LAMP primer sets were designed in this study: c2con, targeting the head maturation protease gene, and S3_c2con, targeting the terminase large subunit gene (Tables 2, 3). The predicted amplicon lengths were 154 bp for the c2con set and 159 bp for the S3_c2con set.

**Table 2:** Characteristics of the c2con primer set.

Primer name	Length (bp)	Primer sequence (5′-3′)
F3	21	TGAAAAGATTGAGGAAACAGG
B3	20	GCACCCATTTGTTCTTGTTC
FIP	46	CTTTGTAATAGCCTACTCCGTCAAT GCTTTCAGTTGGTTTTAATGC
BIP	47	TGTTACAATTACGGAGGTGTCACTT TCCTTTTTCTTCTTCTCGTACT
F2	21	GCTTTCAGTTGGTTTTAATGC
F1c	25	CTTTGTAATAGCCTACTCCGTCAAT
B2	22	TCCTTTTTCTTCTTCTCGTACT
B1c	25	TGTTACAATTACGGAGGTGTCACTT
LF	20	TTCACGTGCTTTCACACCGT
LB	25	TCCGTTACCAAGTAATAAAGGTGCT

**Table 3:** Characteristics of the S3_c2con primer set.

Primer name	Length (bp)	Primer sequence (5′-3′)
F3	19	TGGGTAAAAGACGACGATT
B3	24	CTGTCAGTCATTGATAAATTGAAG
FIP	47	AGCGGGGTTAGCTTTAATGTATTTAGTCATTGGGTTTTCTATTATGC
BIP	47	TAGGCTACACTTTAACACTTGAGGATTTTAGCCATTTTAACAGGGTT
F2	22	GTCATTGGGTTTTCTATTATGC
F1c	25	AGCGGGGTTAGCTTTAATGTATTTA
B2	22	TTTTAGCCATTTTAACAGGGTT
B1c	25	TAGGCTACACTTTAACACTTGAGGA
LF	25	ACTGTCTTTTACTTCGTCGTAATCG
LB	25	TCAAAAGGACTTTATAGGGGCAATC

### 3.3 In silico analysis of LAMP primers for the studied phages

**Figure 2:**
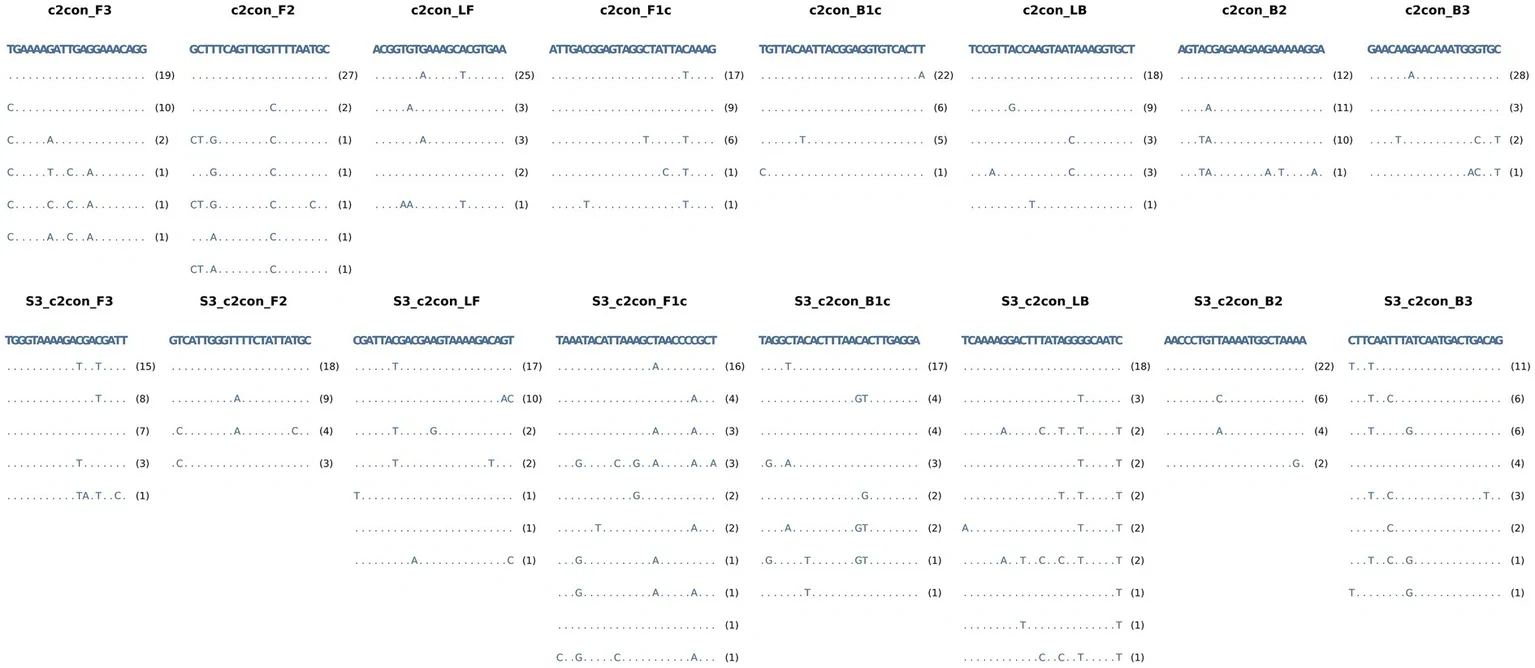
Visualization of phage groups sharing identical primer mismatch patterns. Numbers in parentheses indicate the number of phages in each group.

To further evaluate primer performance, primer sequences were aligned against the target regions of the studied bacteriophage genomes to assess the number of nucleotide mismatches. Supplementary Figure S4 presents a clustered heatmap showing the number of substitutions detected for each primer in each phage genome. Figure 2 illustrates the distribution of phage groups according to mismatch profiles with the primer sets.

The results demonstrated that the target regions of the S3_c2con primer set exhibited higher sequence variability than those targeted by c2con. Variability was assessed based on the mean cumulative number of mismatches between each phage genome and the corresponding primer set, which was higher for S3_c2con. These findings indicate lower conservation of the S3_c2con target region and suggest a potentially reduced detection efficiency for this primer set.

### 3.4 Optimization of primer concentrations

**Figure 3:**
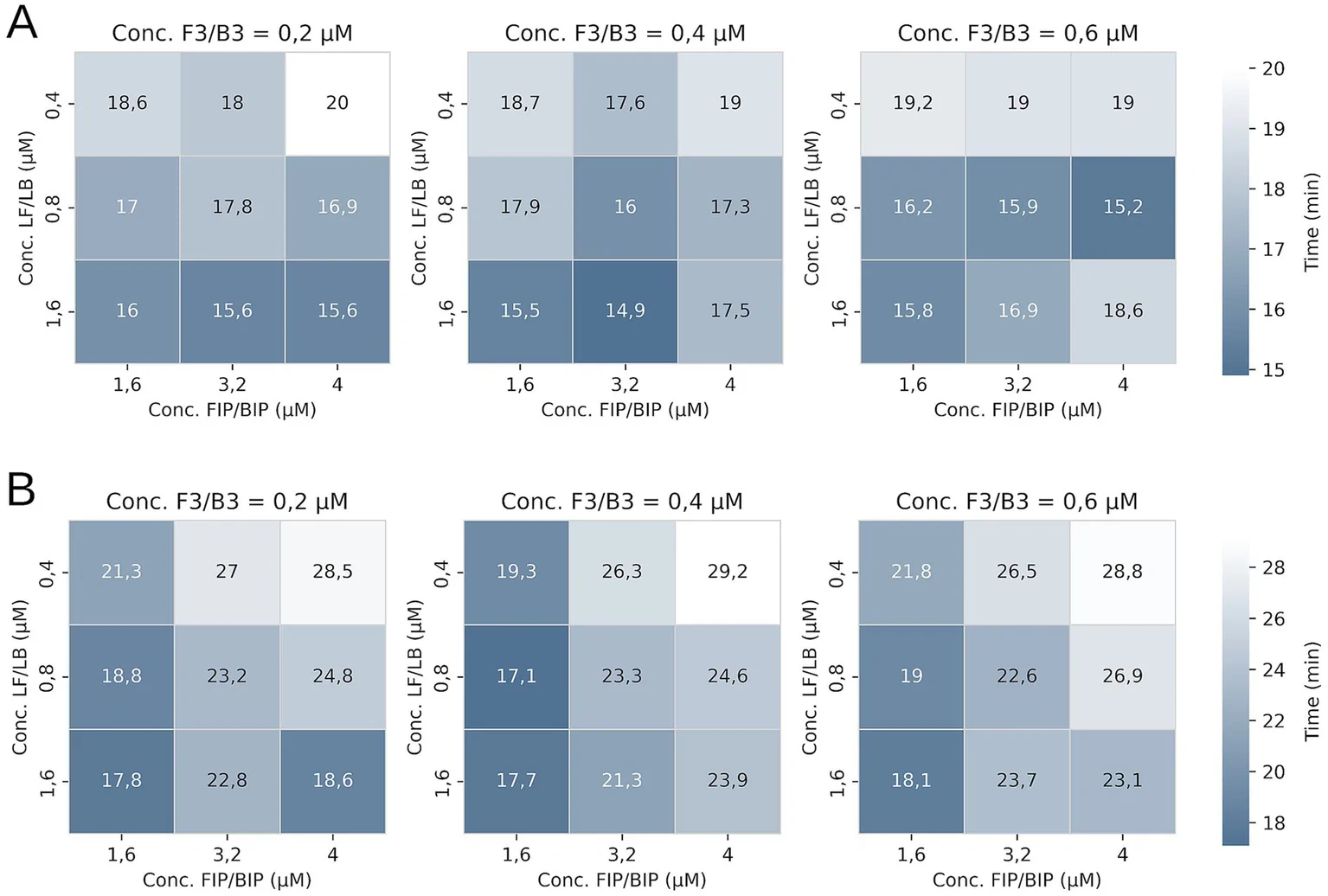
Optimization of primer concentrations for the c2con **(A)** and S3_c2con **(B)** primer sets.

Different primer concentrations were tested to optimize the time-to-positive signal in the LAMP assay. Primer concentrations and corresponding detection times for the c2con and S3_c2con primer sets are shown in Figure 3.

For the c2con set, the shortest detection time (14.9 min) was achieved using 0.4 μM F3/B3, 3.2 μM FIP/BIP, and 1.6 μM LF/LB. For the S3_c2con set, the minimum detection time (17.1 min) was obtained at concentrations of 0.4 μM F3/B3, 1.6 μM FIP/BIP, and 0.8 μM LF/LB.

### 3.5 Optimization of reaction temperature

To verify the suitability of the manufacturer-recommended reaction temperature (65 °C), LAMP efficiency was evaluated at 60, 62, 65, and 70 °C. Phage 309A DNA was used as the template for the c2con primer set, whereas phage 65 DNA was used for S3_c2con.

Analysis of mean detection time and reaction reproducibility based on three technical replicates demonstrated that 65 °C provided the optimal balance between amplification speed and assay stability for both primer sets (Table 4). Although the shortest detection time for c2con (10 min) was observed at 62 °C, amplification was detected in only one of three replicates, indicating poor reproducibility under these conditions. In contrast, amplification at 65 °C was observed in all replicates with only a minor increase in detection time (10.5 min), making these conditions preferable in terms of assay robustness. Based on these results, 65 °C was selected as the optimal amplification temperature for both primer sets.

**Table 4:** Optimization of LAMP reaction temperature.

Primer set	Temperature (°C)	Average time to result (min) ± STD	Number of positive reactions
c2con	60	12.8 ± 0.57	2/3
	62	10	1/3
	65	10.5 ± 0.23	3/3
	70	17.8 ± 0.23	3/3
S3_c2con	60	17.1 ± 0.71	3/3
	62	15.0 ± 0.4	3/3
	65	12.9 ± 0.06	3/3
	70	15.6 ± 0.2	3/3

### 3.6 Limit of detection (LOD)

The analytical sensitivity of the assay was initially evaluated using plasmid standards representing ideal target templates (Table 5). Both primer sets consistently detected target DNA at concentrations ranging from 10^6^ to 10^2^ copies/μL with a 100% positive detection rate. For the c2con primer set, the mean detection time ranged from 9.68 to 17.70 min at plasmid concentrations between 10^6^ and 10^2^ copies/μL. For S3_c2con, amplification times ranged from 10.16 to 17.16 min within the same concentration range. At concentrations below 10^2^ copies/μL, detection probability decreased and amplification times increased.

**Table 5:** Mean detection time and detection frequency for plasmid standards at different concentrations.

Plasmid copies/μL	c2con		S3_c2con	
	Average time (min)	Detection frequency (%)	Average time (min)	Detection frequency (%)
10^6^	9.68	100	10.16	100
10^5^	11.04	100	11.68	100
10^4^	12.09	100	13.01	100
10^3^	14.12	100	14.60	100
10^2^	17.70	100	17.16	100
10^1^	24.56	77.8	24.81	88.9
10^0^	27.33	44.4	21.92	66.7
10^−1^	40.6	11.1	24.60	11.1

Reproducibility analysis demonstrated low intra- and inter-assay coefficients of variation (CV ≤ 7.6%) for both primer sets at concentrations between 10^6^ and 10^3^ copies/μL. At 10^2^ copies/μL, CV values increased to 12–18%, whereas substantially higher variability was observed at 10^1^ copies/μL (Table 6). Thus, the LOD determined using plasmid standards was 10^2^ copies/μL, while the most stable assay performance was observed at concentrations ≥10^3^ copies/μL.

**Table 6:** Reproducibility of plasmid standard detection at different concentrations.

Plasmid copies/μL	c2con		S3_c2con	
	Intra-CV (%)	Inter-CV (%)	Intra-CV (%)	Inter-CV (%)
10^6^	1.63	4.31	1.37	0.19
10^5^	2.80	4.53	2.29	2.16
10^4^	1.58	5.13	2.86	2.21
10^3^	5.05	7.60	2.52	4.66
10^2^	17.79	12.62	5.67	11.51
10^1^	23.33	34.61	18.52	12.61
10^0^	–	–	31.51	11.50
10^−1^	–	–	–	–

The sensitivity of the primer sets was subsequently evaluated using experimental bacterial lysates (Table 7). Lysates containing phages fully complementary to the primer sets were used for the analysis. For c2con, 100% detection was achieved at 10^3^ PFU/mL, whereas S3_c2con provided consistent detection at 10^2^ PFU/mL. Detection frequency for c2con decreased below 10^2^ PFU/mL, while S3_c2con demonstrated slightly greater stability at low phage concentrations.

**Table 7:** Mean detection time and detection frequency for bacterial lysates at different concentrations.

PFU/mL	c2con		S3_c2con	
	Average time (min)	Detection frequency (%)	Average time (min)	Detection frequency (%)
10^8^	10.66	100	8.84	100
10^7^	10.81	100	10.43	100
10^6^	11.82	100	11.94	100
10^5^	13.08	100	13.37	100
10^4^	14.88	100	14.33	100
10^3^	17.39	100	16.53	100
10^2^	22.83	77.8	17.28	100
10^1^	23.10	77.8	21.65	91.7
10^0^	15.92	66.7	23.50	91.7
10^−1^	22.49	77.8	24.57	91.7

High reproducibility was observed at concentrations ≥10^4^ PFU/mL (CV < 7% for c2con and < 6% for S3_c2con). At the LOD level, coefficients of variation increased (c2con: intra-assay 6.6–15.7%, inter-assay 3.9–24.2%; S3_c2con: intra-assay 4.1–11.2%, inter-assay 13.6–15.4%), indicating assay performance near the detection threshold. At concentrations below the LOD, variability increased markedly, confirming stable detection only at or above the established LOD values (Table 8). Thus, the LOD in bacterial lysates was determined to be 10^3^ PFU/mL for c2con and 10^2^ PFU/mL for S3_c2con.

**Table 8:** Reproducibility of bacterial lysate detection at different concentrations.

PFU/mL	c2con		S3_c2con	
	Intra-CV (%)	Inter-CV (%)	Intra-CV (%)	Inter-CV (%)
10^8^	3.96	5.44	2.27	3.53
10^7^	1.30	3.61	2.84	5.07
10^6^	2.25	2.36	1.66	6.16
10^5^	1.93	4.78	1.26	4.62
10^4^	3.74	6.34	1.25	3.96
10^3^	6.63	3.94	4.12	13.65
10^2^	15.73	24.18	11.16	15.44
10^1^	21.29	19.34	19.71	21.60
10^0^	12.74	30.06	21.31	30.84
10^−1^	20.14	55.66	50.76	22.10

**Figure 4:**
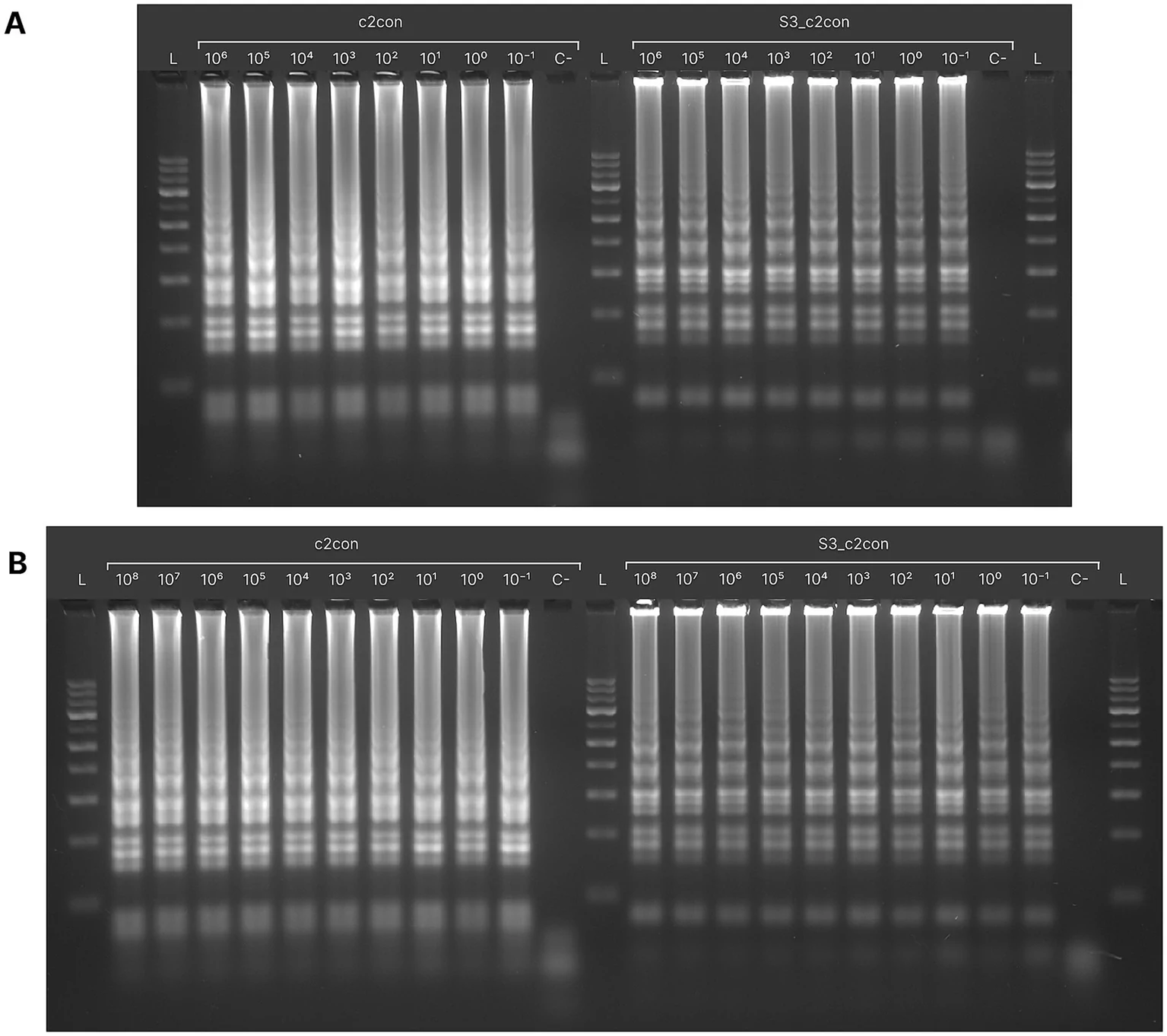
**(A)** Electrophoretic profiles of plasmid standard amplification products at different concentrations (10^6^–10^−1^ copies/μL). **(B)** Electrophoretic profiles of bacterial lysate amplification products at different concentrations (10^8^–10^−1^ PFU/mL). L, 100 bp DNA ladder; C-, negative control.

Electrophoretic analysis of LAMP products was performed to confirm the formation of the characteristic ladder-like amplification pattern (Notomi et al., 2000) (Figure 4). Electrophoretic profiles demonstrated the typical ladder structure associated with LAMP amplification, whereas no specific amplification patterns were observed in negative controls.

**Figure 5:**
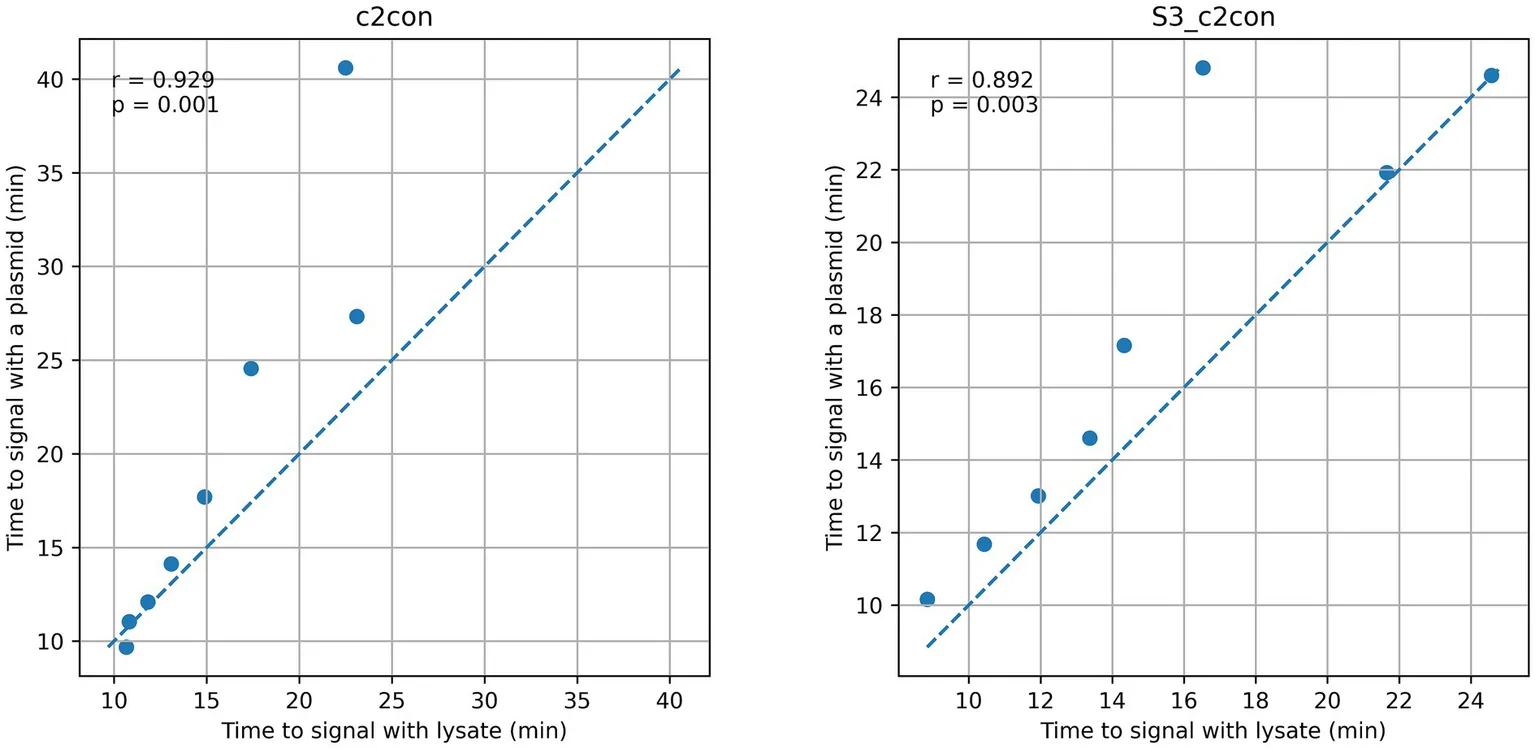
Changes in amplification kinetics with decreasing template concentration.

The potential effect of bacterial lysate components on amplification efficiency was evaluated by comparing LAMP reaction kinetics obtained using plasmid standards and bacterial lysates across serial dilutions (Figure 5). For each dilution level, the time-to-positive signal obtained for plasmid DNA and lysate samples was compared.

Concentration ranges from 10^6^ to 10^−1^ copies/μL (plasmid standard) and PFU/mL (lysate) were treated as equivalent dilution steps, since the aim of the analysis was to compare reaction kinetics rather than establish absolute quantitative equivalence between matrices. A strong positive correlation was observed between detection times for plasmid standards and bacterial lysates for both primer sets (*r* = 0.929, *p* = 0.001 for c2con; *r* = 0.892, *p* = 0.003 for S3_c2con), indicating similar amplification kinetics as template concentration decreased.

These results suggest a comparable dependence of amplification rate on template concentration in both systems. Although the findings do not exclude a possible systematic influence of bacterial lysate components on absolute detection times, no pronounced distortion of the amplification kinetics relative to plasmid standards was observed.

### 3.7 Ratio of encapsidated and free phage DNA

To estimate the actual proportion of phage genome copies present within intact virions in bacterial lysates, the amount of detectable phage DNA was compared before and after DNase treatment. The analysis was based on the assumption that DNA enclosed within intact phage capsids is protected from DNase digestion, whereas free extracellular phage DNA is completely degraded. DNase treatment resulted in a substantial decrease in detectable signal: a 6.1-fold reduction was observed using the C2 primer pair and an 8.2-fold reduction using the PC2 primer pair relative to untreated samples. Based on these data, the proportion of capsid-associated phage DNA was estimated at approximately 12–16%, whereas 84–88% of the detected DNA corresponded to free extracellular DNA (Table 9).

**Table 9:** Estimation of the ratio between capsid-associated and free phage DNA copies.

Parameter	C2	PC2
Ct before DNase treatment	16.0	17.9
Ct after DNase treatment	19.2	21.3
ΔCt	3.2	3.4
Amplification efficiency (E)	1.76	1.86
Fold reduction (K = E^ΔCt^)	6.1	8.2
Fraction of DNA remaining after DNase treatment (1/K)	0.164	0.122
Capsid-associated DNA fraction, %	16%	12%
Free DNA fraction, %	84%	88%

### 3.8 Evaluation of qualitative phage detection

**Figure 6:**
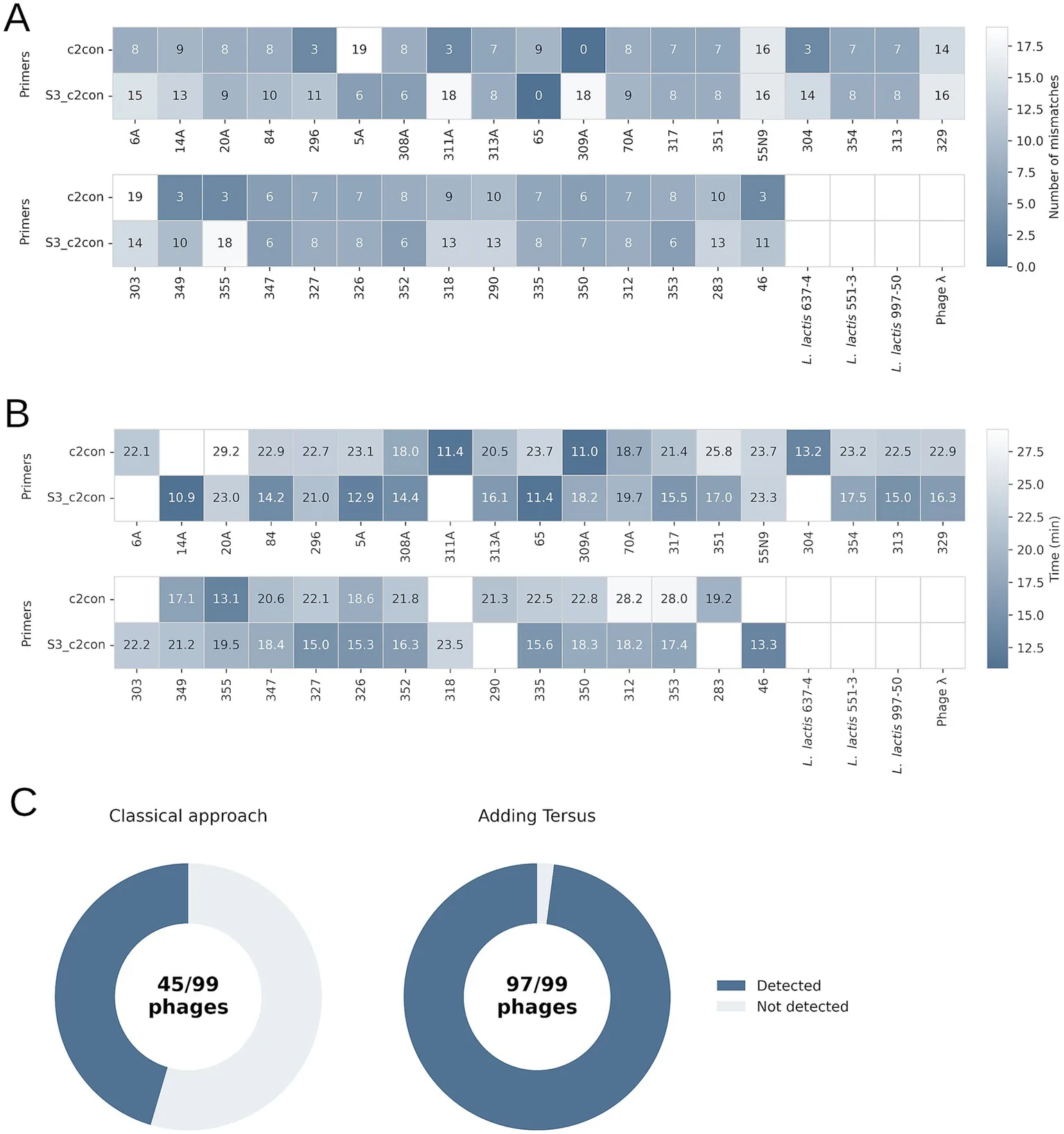
**(A)** Number of mismatches between primer sets and target or non-target genomes. Empty cells correspond to non-target genomes lacking primer-binding sites. **(B)** Detection times for the 34 studied bacteriophages and non-target samples. Empty cells indicate no amplification within 30 min. **(С)** Number of published c2 phages predicted to be detectable using conventional LAMP and LAMP supplemented with high-fidelity Tersus polymerase. Left: Phages containing ≤9 mismatches for c2con and ≤13 mismatches for S3_c2con. Right: Phages containing ≤19 mismatches with at least one primer set.

The ability of both primer sets to qualitatively detect the 34 studied bacteriophage isolates containing different numbers of primer-template mismatches was evaluated (Figure 6A). Nearly all isolates were successfully detected within 30 min of amplification (Figure 6B).

Among non-target samples, amplification was observed in two of six technical replicates containing *L. lactis* strain 551–3 DNA (Supplementary Table S2), likely indicating contamination of the sample with a small amount of c2 phage DNA. This assumption is supported by the metagenomic analysis, which identified 0.02% Ceduovirus DNA in this sample, exceeding the corresponding values observed in the remaining samples. Taxonomic classification profiles of samples are provided in Supplementary File S1.

Threshold mismatch values associated with a statistically significant decrease in amplification probability differed between the two primer sets. For c2con, a significant association was observed at >9 mismatches (*p* = 0.048; OR = 12), whereas for S3_c2con a stronger and more statistically significant association was identified at >13 mismatches (*p* = 0.0028; OR = 33).

Alternative endpoint detection approaches were evaluated for the developed LAMP assay, including fluorescence under UV illumination, turbidity-based detection, and colorimetric detection (Supplementary Figure S5). SYBR Green I fluorescence was observed in primer-containing mixtures even before amplification, most likely due to dye binding to oligonucleotide primers and/or primer-derived secondary structures. Since SYBR Green I can detect low amounts of nucleic acids, including 1–2 ng of a synthetic 24-mer oligonucleotide (Merck KGaA, 2022; Product Information Sheet) and dsDNA in the range of approximately 1–1,000 ng/mL (Thermo Fisher Scientific, n.d.; SYBR Green I dsDNA Assay), this background fluorescence was expected and was not considered reliable evidence of target-specific amplification. In contrast, turbidity-based detection successfully distinguished positive reactions from negative controls, and colorimetric detection produced a clear positive result after 60 min of amplification.

### 3.9 Validation of the assay using industrial samples

LAMP amplification was performed using cheese whey samples collected from dairy plants and previously confirmed to contain bacteriophages. The effect of preliminary thermal treatment (95 °C, 10 min) on time-to-positive signal was additionally evaluated (Table 10).

**Table 10:** Time-to-positive signal (min, mean ± STD) for whey samples containing different phage titers. ND, not detected.

Whey sample	Phage titer (PFU/mL)	c2con (heat) (min ± STD)	c2con (no heat) (min ± STD)	S3_c2con (heat) (min ± STD)	S3_c2con (no heat) (min ± STD)
1	2 × 10^7^	14.5 ± 0.06	22.6 ± 2.34	ND	ND
2	1 × 10^6^	21.7 ± 1.93	33.6 ± 6.45	ND	ND
3	7 × 10^6^	25.7 ± 5.87	32.9 ± 1.60	ND	ND

Only the c2con primer set enabled successful detection of phages in whey samples. Preliminary heat treatment proved critical, reducing the time-to-positive signal by 7.2–11.9 min. The absence of amplification using the S3_c2con primer set may be associated with the high genetic variability of phages present in whey samples and the presence of numerous mismatches in primer-binding regions, which likely impaired amplification efficiency.

### 3.10 Increasing LAMP tolerance to primer-template mismatches

According to Li Y. et al., the addition of a high-fidelity DNA polymerase possessing 3′ → 5′ proofreading activity to LAMP reactions may reduce the negative effects of primer-template mismatches, particularly near the primer 3′-end, thereby improving amplification efficiency (Li et al., 2019). Based on this approach, additional experiments were performed using Tersus high-fidelity polymerase for phage samples that either produced delayed positive signals (>30 min) or failed to amplify with one of the primer sets (Supplementary Table S2).

In most cases, addition of Tersus polymerase reduced detection time by 2.1–25.3 min, indicating improved amplification efficiency in the presence of primer-template mismatches. However, for phage 296 analyzed using the c2con primer set, the opposite effect was observed, with a slight increase in amplification time. This effect may be related to template-specific structural features, which could in turn alter reaction kinetics. In this case, mismatches may not have been the limiting factor, while polymerase addition altered the balance between amplification speed and specificity.

Bacteriophage 46 was not detected by the c2con primer set despite containing only three mismatches within primer-binding regions. Similarly, no amplification signal was observed for this phage with the S3_c2con set following addition of Tersus polymerase. One possible explanation is the high level of bacterial DNA contamination identified by metagenomic analysis, which may have negatively affected amplification efficiency.

Overall, Tersus polymerase reduced the negative impact of primer–template mismatches on amplification efficiency and expanded the range of detectable phages, although it did not completely eliminate the relationship between mismatch number and amplification probability.

### 3.11 *In silico* analysis using publicly available phage genomes

Specificity of the developed assay toward other widespread Lactococcus phage groups was evaluated *in silico* using 214 complete genomes of 936-group phages, 38 genomes of P335-group phages, and 68 genomes representing the less prevalent 1358, Q54, P087, 1706, 949, P034, and KSY1 groups. No primer-binding sites meeting the predefined mismatch criteria were identified, indicating the absence of predicted cross-reactivity with these phage groups.

To evaluate the potential applicability of the developed LAMP primers to additional c2 phages, mismatch analysis was performed using publicly available genome sequences. The analysis demonstrated that for 97 of 99 c2 bacteriophages, the number of mismatches in at least one primer set did not exceed the acceptable threshold (maximum six mismatches per primer) (Supplementary Figures S6–S8). However, the average mismatch number for these phages exceeded that observed for the genomes used during primer design, suggesting the possibility of reduced amplification efficiency.

Among the 99 publicly available c2 phages, the standard LAMP assay without Tersus polymerase was predicted to detect 45 phages (45.5%), whereas inclusion of Tersus theoretically increased the number of detectable phages to 97 (98%) (Figure 6C). Detectability in the presence of Tersus was estimated using an empirical criterion derived from experimental data: phages were considered potentially detectable if they contained ≤19 mismatches with at least one primer set.

## 4 Discussion

*Lactococcus* bacteriophages, including c2-group phages, represent a major risk for the dairy industry as a key cause of fermentation failures and associated economic losses (Marcó et al., 2012). This creates a strong demand for rapid and reliable detection methods. LAMP is considered a promising approach due to its high speed, low cost, and resistance to inhibitors compared with PCR; however, its application is limited by the high genetic variability of phages and the complexity of primer design (6–8 primers), which requires strict selection of conserved genomic regions (Chen et al., 2017; Li et al., 2019).

A critical determinant of LAMP performance is primer-template complementarity. Mismatches, particularly within the 3′ regions of FIP and BIP primers, can significantly reduce efficiency or completely inhibit amplification, whereas outer primers (F3/B3) are generally more mismatch-tolerant due to their mainly initiating role (Li et al., 2019). It has been shown that up to ~20% of primer-template mismatches may be tolerated without a substantial loss of efficiency, provided that they are not located at the 3′ ends (Carascal et al., 2024). In addition, up to 10 mismatches in less critical positions within a primer set have been reported not to prevent successful amplification (Furuyama et al., 2018).

Previous studies have indicated that optimal targets for phage detection include genes encoding structural proteins (major tail protein (Brinks et al., 2018), major capsid protein (Labrie and Moineau, 2000)) and DNA packaging proteins, including terminase (Muhammed et al., 2017). In our analysis of 12 production isolates of *Ceduovirus* phages, the most conserved regions were identified in the genes encoding the large terminase subunit and the head maturation protease. Despite the relative conservation of these regions, *in silico* evaluation revealed an average of 8.9 mismatches between the designed primers and target sequences, reflecting the inherent genetic variability of these loci. To address this heterogeneity, two independent primer sets were developed; however, mismatches in critical positions could not be fully eliminated.

**Figure 7:**
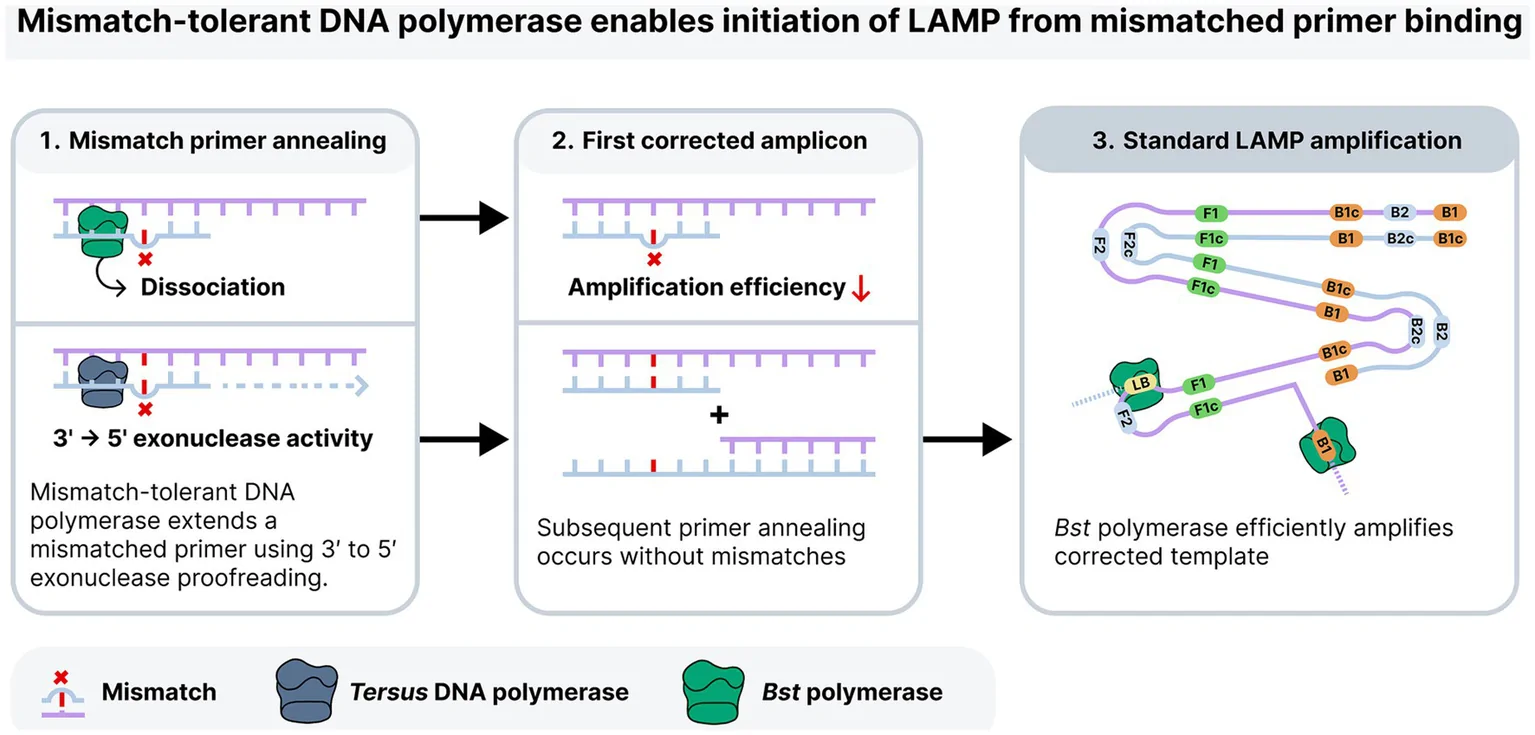
Proposed mechanism of mismatch-tolerant LAMP amplification in the presence of *Tersus* DNA polymerase.

The inability to completely avoid critical mismatches at the primer design stage highlights the need for alternative strategies to improve reaction robustness, including the use of high-fidelity polymerases capable of compensating for reduced efficiency caused by 3′ mismatches (Li et al., 2019; Zhou et al., 2019). In this study, we report for the first time the use of the high-fidelity Tersus DNA polymerase (Evrogen) in LAMP reactions. The presumed mechanism is related to the 3′ → 5′ exonuclease activity of high-fidelity polymerases. When a primer contains a mismatch close to its 3′ end, extension by *Bst* DNA polymerase alone may be inefficient because the mismatched terminus is poorly extended, which can lead to dissociation of the enzyme from the primer-template complex. A proofreading polymerase can remove the mismatched 3′ nucleotide, generating a correctly paired primer terminus that can subsequently be extended during the LAMP reaction. Thus, the additional high-fidelity polymerase may partially rescue amplification from templates containing primer–template mismatches, thereby increasing the tolerance of the assay to sequence variability (Figure 7). The addition of Tersus broadened the detection range, reducing time-to-signal by 2–25 min and enabling detection of all tested bacteriophages while maintaining specificity (no detection of *L. lactis* or phage *λ*). Moreover, *in silico* analysis suggested that the modified system could potentially detect up to 98% of known *Ceduovirus* representatives. However, experimental validation on an expanded phage panel is required to confirm this prediction.

Direct LAMP assays specifically targeting c2-type *Lactococcus* phages are scarcely reported in the literature; instead, multiplex systems targeting c2, 936, and P335 groups are more common. For qPCR-based detection of Lactococcus phages, reported sensitivity is approximately 10^2^–10^3^ PFU/mL, which is comparable to the values obtained in this study (Ly-Chatain et al., 2011; Muhammed et al., 2017). To date, only one LAMP assay targeting lactic acid bacterial phages has been described, namely for the thermophilic phage P680 (Skunavirus, 936 group) (Brinks et al., 2018), with a detection limit of approximately 10^3^ PFU/mL in whey. In the present study, a comparable range (10^2^–10^3^ PFU/mL) was achieved in bacterial lysates using only thermal lysis without additional DNA purification, indicating the feasibility of simplified sample preparation while maintaining analytical sensitivity.

The performance and sensitivity of the assay were evaluated using two matrix types: a plasmid standard and bacterial lysates. On the plasmid template, both primer sets achieved 100% detection at 10^2^ copies/μL, with stable amplification kinetics observed at concentrations ≥10^3^ copies/μL. The obtained LOD (10^2^ copies/μL) is consistent with previously reported ranges for LAMP-based viral genome detection in standardized systems (Shuryaeva et al., 2022; Suther et al., 2022; Koryukov et al., 2024; Mozioğlu et al., 2025). In bacterial lysates, the detection limit was 10^3^ PFU/mL for the c2con set and 10^2^ PFU/mL for the S3_c2con set. Increased variability at LOD levels reflects the stochastic nature of amplification at low template copy numbers (Hardinge and Murray, 2020). These values are comparable to previously reported LAMP assays for viruses and bacteriophages in complex matrices (Chotiwan et al., 2017; Brinks et al., 2018).

The differences between plasmid and biological matrices are primarily due to the discrepancy between PFU and genome copy number: PFU reflects only infectious particles, whereas lysates also contain non-infectious virions and free DNA detectable by LAMP (Benavent-Celma et al., 2026). This is supported by DNA partitioning data, which showed that the majority of DNA (~86%) was extracellular, with the estimated genome copy number exceeding PFU by approximately seven-fold. Thus, plasmid standards reflect the analytical limit of the assay, whereas lysates, containing real phage particles rather than synthetic DNA, represent its practical performance on biologically relevant samples. Importantly, matrix effects did not distort reaction kinetics, as evidenced by a strong correlation in amplification time between matrices (*r* = 0.892–0.929; *p* < 0.01). The assay was also successfully validated on industrial whey samples, confirming its applicability under real production conditions. In the proposed test system, both primer sets are intended to be used in parallel but in separate reaction tubes, thereby increasing the coverage of genetically diverse c2 phages while allowing the results for each primer set to be interpreted independently.

This study has several limitations. First, the empirically derived mismatch threshold (≤19 for Tersus) was based on a limited isolate panel and requires validation on independent datasets. Second, successful detection in industrial whey was demonstrated only for the c2con primer set; the lack of amplification with S3_c2con was not confirmed by sequencing and remains inferential. Finally, potential contamination of industrial samples with unknown phage strains may affect specificity. Although no cross-reactivity with 936 and P335 phages was observed in silico, this finding was not further confirmed by *in vitro* testing due to limited availability of representative isolates from these phage groups within our collection. We acknowledge this as a limitation of the present study, and further in vitro validation on an expanded panel of *Lactococcus* phage isolates, including representatives of the 936 and P335 groups, would strengthen confidence in assay specificity.

## 5 Conclusion

In this study, a LAMP-based assay for the detection of c2-type bacteriophages infecting *Lactococcus* was developed. Both primer sets demonstrated sensitive detection at levels compatible with industrial monitoring requirements and remained functional in real industrial whey samples, confirming their practical applicability.

For the first time, a high-fidelity DNA polymerase Tersus was applied in a standard (non–RT) LAMP assay, increasing tolerance to primer–template mismatches and expanding the detectable range of genetically diverse phages. *In silico* analysis indicated that the modified system could potentially cover up to 98% of described c2 phages, highlighting its promise for industrial monitoring applications.

Overall, LAMP-based detection of c2 phages is determined by the interplay between target genetic variability, matrix effects, and mismatch tolerance of the amplification system. Improved robustness via high-fidelity polymerase supplementation, combined with demonstrated performance in industrial samples, supports the proposed approach as a basis for diagnostic tools adapted to highly variable lactic acid bacteriophages. Further validation on expanded phage collections and diverse dairy matrices is required to define the full applicability and robustness of the system.

## Data Availability

The datasets presented in this study can be found in online repositories. The names of the repository/repositories and accession number(s) can be found at: https://www.ncbi.nlm.nih.gov/, PQ062249; PQ594011; PQ594013; PQ594016; PQ062250; PQ594018; PQ594019; PQ594020; PQ594021; PQ582088; PQ594015; PQ594014; PZ517723; PZ517724; PZ517725; PZ517726; PZ517727; PZ517728; PZ517729; PZ517730; PZ517731; PZ517732; PZ517733; PZ517734; PZ517735; PZ517736; PZ517737; PZ517738; PZ517739; PZ517740; PZ517741; PZ517742; PZ517743; PZ517744.

## Supplementary material

SUPPLEMENTARY FIGURE S1 Alignment of the terminase large subunit gene of 12 bacteriophages used in primer design.

SUPPLEMENTARY FIGURE S2 Alignment of the head maturation protease gene of 12 bacteriophages used in primer design.

SUPPLEMENTARY FIGURE S3 Comparative whole-genome alignment of the c2 phage isolates analyzed in this study.

SUPPLEMENTARY FIGURE S4 Heatmap of primer-template mismatches with the studied c2 phages with clustering.

SUPPLEMENTARY FIGURE S5 Alternative endpoint detection approaches evaluated for the LAMP assay. **(A)** Fluorescence detection under UV illumination. **(B)** Turbidity-based detection. **(C)** Colorimetric detection based on color change. NTC, no-template negative control; T^+^, positive sample containing phage DNA.

SUPPLEMENTARY FIGURE S6 Heatmap of primer-template mismatches with publicly available c2 phages with clustering.

SUPPLEMENTARY FIGURE S7 Heat map of the sum of primer-template mismatches with publicly available c2 phages.

SUPPLEMENTARY FIGURE S8 Visualization of phage groups from the literature with identical primer mismatches. The number of phages in each group is indicated in parentheses.
