# Supplementary material for: Development and validation of a LAMP assay for detection of genetically diverse Lactococcus c2-type bacteriophages in dairy environments
Source: Front Microbiol. 2026 Aug 10;17:1912103. doi: 10.3389/fmicb.2026.1912103 (PMC13500662; doi:10.3389/fmicb.2026.1912103)
Supplement: Supplementary file 1 [file Table_1.docx]

# **Supplementary Tables**

## **Table S1.** General characteristics of the genomes of the studied bacteriophages

| Phage | NCBI genome ID | Genome size (bp) | GC composition (%) | ORF count |
| --- | --- | --- | --- | --- |
| 20А | PQ062249 | 21909 | 35.75 | 40 |
| 70A | PQ594011 | 21488 | 35.79 | 38 |
| 84 | PQ594013 | 21957 | 35.89 | 37 |
| 5A | PQ594016 | 21693 | 35.96 | 37 |
| 296 | PQ062250 | 22667 | 35.91 | 41 |
| 14A | PQ594018 | 22187 | 35.99 | 38 |
| 6A | PQ594019 | 21605 | 35.60 | 38 |
| 308A | PQ594020 | 22697 | 36.06 | 38 |
| 65 | PQ594021 | 22447 | 35.96 | 40 |
| 311A | PQ582088 | 21837 | 36.25 | 35 |
| 309A | PQ594015 | 22059 | 35.92 | 40 |
| 313A | PQ594014 | 22072 | 35.82 | 38 |
| 317 | PZ517723 | 20602 | 35.90 | 35 |
| 351 | PZ517724 | 21811 | 35.79 | 39 |
| 55N9 | PZ517725 | 22213 | 35.68 | 39 |
| 304 | PZ517726 | 22023 | 35.42 | 41 |
| 354 | PZ517727 | 22149 | 35.79 | 38 |
| 313 | PZ517728 | 22149 | 35.74 | 38 |
| 329 | PZ517729 | 22332 | 35.85 | 39 |
| 303 | PZ517730 | 22055 | 35.41 | 41 |
| 349 | PZ517731 | 21475 | 35.98 | 38 |
| 355 | PZ517732 | 21928 | 35.88 | 38 |
| 347 | PZ517733 | 22114 | 35.92 | 39 |
| 327 | PZ517734 | 22152 | 35.85 | 39 |
| 326 | PZ517735 | 22149 | 35.79 | 39 |
| 352 | PZ517736 | 22774 | 36.02 | 38 |
| 318 | PZ517737 | 22264 | 35.98 | 39 |
| 290 | PZ517738 | 22042 | 35.77 | 38 |
| 335 | PZ517739 | 22149 | 35.79 | 38 |
| 350 | PZ517740 | 22219 | 35.67 | 42 |
| 312 | PZ517741 | 22149 | 35.81 | 39 |
| 353 | PZ517742 | 22774 | 36.01 | 38 |
| 283 | PZ517743 | 22042 | 35.73 | 37 |
| 46 | PZ517744 | 22524 | 35.91 | 39 |

## **Table S2.** Changes in detection time following addition of the high-fidelity Tersus polymerase. Numbers in parentheses indicate positive technical replicates. ND, no detection

| **Organism** | **Primer set** | **Mismatches** | **Detection time without Tersus (min)** | **Detection time with Tersus (min)** | **Δ Detection time (min)** |
| --- | --- | --- | --- | --- | --- |
| 14A | c2con | 9 | 35.4 | 23.2 | 12.2 |
|  | S3_c2con | 13 | 10.9 | 7.8 | 3.1 |
| 6A | c2con | 8 | 22.1 | 17.6 | 4.5 |
|  | S3_c2con | 15 | ND | ND | ND |
| 311A | c2con | 3 | 11.4 | 9.3 | 2.1 |
|  | S3_c2con | 18 | ND | ND | ND |
| 296 | c2con | 3 | 22.7 | 23.9 | −1.2 |
|  | S3_c2con | 11 | 21.0 | ND | ND |
| 304 | c2con | 3 | 13.2 | 10.3 | 2.9 |
|  | S3_c2con | 14 | 37.1 | 21.8 | 15.3 |
| 303 | c2con | 16 | ND | ND | ND |
|  | S3_c2con | 14 | 28.2 | 17.9 | 10.3 |
| 318 | c2con | 9 | 41.0 | 23.3 | 17.7 |
|  | S3_c2con | 13 | 23.5 | 18.5 | 5.0 |
| 290 | c2con | 10 | 21.3 | 15.2 | 6.1 |
|  | S3_c2con | 13 | ND | 24.1 | Detected only after Tersus addition |
| 283 | c2con | 10 | 19.2 | 14.5 | 4.7 |
|  | S3_c2con | 13 | 52.1 | 26.8 | 25.3 |
| 46 | c2con | 3 | ND | ND | ND |
|  | S3_c2con | 11 | 13.3 | ND | ND |
| *L. lactis* 551-3 | c2con | – | ND | ND | ND |
|  | S3_c2con | – | 19.1 (2/6) | 17.8 (2/6) | 1.3 |
| *L. lactis* 997-50 | c2con | – | ND | ND | ND |
|  | S3_c2con | – | ND | ND | ND |
| Phage λ | c2con | – | ND | ND | ND |
|  | S3_c2con | – | ND | ND | ND |
