# Supplementary material for: Development and validation of a LAMP assay for detection of genetically diverse Lactococcus c2-type bacteriophages in dairy environments
Source: Front Microbiol. 2026 Aug 10;17:1912103. doi: 10.3389/fmicb.2026.1912103 (PMC13500662; doi:10.3389/fmicb.2026.1912103)
Supplement: Supplementary file 2 [file Data_Sheet_1.ZIP › Supplementary File S1/L.lactis_551_3_krona.html]

Javascript must be enabled to view this page.

magnitude
magnitudeUnassigned

Illumina
ONT

2080004917448

20542
39949791

00
19871

00
19871

19871
00

19871
00

19871
00

19871

5
0

0
5

5
0

5
0

5
0

5

00
2933

4
0

0
4

4
1

3
0

3

00
2893

20
0

0
3

3
1

2

17
0

0
17

1

16

00
2693

2693
00

12
2693

256

2

1

1

21

2

3

1

5
1

0
2

0
1

1
0

1

0
1

1
0

1

2
0

0
2

0
2

2

108662
120

5
0

0
5

0
5

5
2

1

2

0
7

0
7

3
7

0
4

4

00
142

92
00

92
00

5
2

1

1

1

42
01

1

21

1

0
5

1
5

3
0

3

1
0

1

27
1

4
22

0
5

0
5

5

0
13

0
13

13

4
0

4
0

0
4

4

0
10801

10801
0

10801
0

30
10801

240

3

10528

38390077
00

38390077
00

38390077
00

38390077
00

00
38390077

38390077

250
3744

19
0

0
19

0
19

1
0

1

0
2

2

0
1

1

3
0

1

2

1
12

9

1

1

0
45

0
45

0
45

45
0

45

100
1023

611
20

4
0

1
0

1

3
0

3

5
0

1
0

1

4
0

4

3
0

3
0

3

371
00

110
371

4

1

18

1

2

1

0
10

0
10

1

7

2

2
0

2
0

2
0

1

1

00
201

0
8

5
0

5

0
3

3

00
81

61
81

1

1

0
4

0
2

2

2
0

2

00
91

91
00

91
61

1

2

3
34

19
4

7
0

0
7

7

8
0

0
8

8

0
12

2
12

4
0

4

2
0

2

4
0

4

107
0

105
2

95
6

14
52

14

10

3

3

8

37
1

30

6

8
0

8
0

7

1

0
2

2
0

2
0

2

7
0

7
0

6
0

6
0

6

1
0

1

351
00

1
0

1
0

1
0

1

341
00

341
00

00
11

11

0
33

33

0
1

0
1

1
0

1
0

1
0

1

8
0

8
0

8
0

0
8

1
8

2

5

51
00

51
00

51
00

00
51

51

00
522

31
00

31
00

0
1

0
1

0
1

1

00
21

00
21

1
0

1

1
0

1

1
0

1

1
0

1
0

1
0

1

481
00

0
2

2
0

0
2

2
0

1

1

30
0

30
2

1
0

0
1

1

0
8

2
0

2

2
0

1

1

1
0

1

1
0

1

0
1

1

0
1

1

0
4

0
1

1

3
0

1

1

1

11
1

0
2

2

4
1

1

1

1

0
1

1

2
0

2

0
1

1

0
1

0
1

1

3
0

0
1

1

1
2

1

0
1

0
1

0
1

0
1

1

00
21

21
00

00
11

00
11

11

0
1

0
1

1

0
1

0
1

1
0

0
1

1

0
11

0
10

0
10

0
10

3

2

3

1

1

1
0

0
1

0
1

1

1
0

0
1

1
0

0
1

1

11182762188

164858
10

00
51

0
2

2
0

0
2

1
0

1

0
1

1

00
31

2
0

0
2

0
2

2

00
11

11
00

00
11

11

164777
00

164777
6360

318
1221

31

741
861

4

4

4

7

2

13

1

4

1

1

7

2

2

10

1

1

11

1

20

20

4

11
0

8

1

2

0
3

0
1

1

2

1

2

0
1

0
1

0
1

1

4
0

1

1

2

2
0

2

1

11

4172
2173

181

28

41

2

99

5

68

55

26

28

288

2

47

22

32

39

138

16

1

37

10

87

116

61

5

25

49

167

5

1

44

14

26

98

136

0
3

3

0
1

1

0
3

1

2

22671

15842

0
1

1

2347

1

1769
20

1

3

1

2

1720

4

1

5

1

4

1

1

1

1

3

1
0

1

20
611

25

31

31

41

13

2
0

1

1

30
33

1

2

0
2

2

1

6

100
71

1

1

1

3

1

2

1

1

1

5

1

3

1

1

1

1

1

1

1

1

0
1

1

7033
21403

54

1383

1

5

40
29

3

1

7

1
0

0
1

1
0

0
1

1
0

1

1
0

1
0

0
1

1
0

1

1926573915159
8004737

0
3793

3793
6

0
264

264
5

0
5

5

11
2

5

4

122
243

2

24

3

11

1

61

8

1

1

3

5

1

0
3523

3523
0

3523
930

8

9

618

9

10

181

62

5

211

41

28

6

11

7

6

4

8

2

24

12

27

1

4

1299

1
0

0
1

1
0

1
0

1

731
00

00
731

721
00

4
0

0
4

1

1

2

1
9

0
3

1

2

1
0

1

0
4

1

3

00
231

231
20

3

1

2

1

1

3

3

1

3

2

1

1

6
0

6
0

1

4

1

9
0

7
9

1

1

0
21

21
0

15

1

5

1
0

1
0

0
1

1

3
0

3
0

3
0

0
3

3
0

3

5840
4494725

60
0

0
60

60
0

0
60

60

5540
154464

1590
41541

38021
1020

0
1

1

21
0

21

2
0

2

0
3

3

0
21

19

2

0
2

2

0
6

6

0
4

1

3

0
1

1

1
0

1

9300
33071

53

230

49

281

6

3

47

9

54

588

25

20

12

19

7

8

13

13

8

4

20

76

12

375

3

1

10

506

30

15

51

18

3

50

7

1

3

0
18

18

0
7

2

3

2

0
9

5

3

1

3
0

3

0
3

3

0
3

3

3
0

3

2
0

1

1

3
0

3

1
0

1

0
4

4

0
214

214

3
10

3

2

2

25
1

4

7

13

1
0

1

0
2

2

12
3

4

2

2

1

0
1

1

0
1

1

4
0

1

3

2
0

2

0
1

1

0
1

1

1
0

1

28
193

3
0

2

1

1
0

1

6
0

1

2

1

2

31
2

2

3

1

2

2

3

6

1

1

2

1

2

1

2

0
2

2

2
0

2

3
0

3

1
6

1

1

1

2

3
0

3

1
0

1

2
0

1

1

0
3

3

3
0

3

10
1

1

8

1
0

1

1
0

1

1
11

3

4

3

10
0

2

2

1

1

4

4
10

2

1

3

0
1

1

6
0

1

2

1

1

1

0
1

1

23
0

23

2
0

1

1

12
2

4

1

1

3

1

0
3

2

1

3
0

1

2

0
5

5

3
0

3
0

0
3

3

0
2

2
0

2
0

2

1727
89

1479
159

2
0

2

0
6

6

2
0

1

1

0
2

1

1

0
4

1

1

2

9
17

1

2

3

2

643
165

2

1

3

7

3

3

3

13

4

4

2

5

11

10

4

6

6

5

2

9

1

2

1

3

5

1

17

2

5

9

12

1

3

1

44

3

1

4

7

8

5

4

2

6

3

62

1

13

50

3

2

3

1

6

6

1

3

2

1

2

2

1

6

6

6

5

5

2

17

19

10
55

1

1

5

5

2

1

2

12

5

1

4

1

2

1

1

1

5
0

1

4

24
0

1

1

22

3
0

2

1

1
0

1

0
2

2

4
0

4

12
74

2

16

3

1

2

1

7

3

1

5

2

2

1

7

3

1

4

1

187
476

3

4

2

1

3

2

1

3

5

9

1

1

1

14

2

1

4

11

12

195

1

6

4

1

2

0
9

0
6

4

2

0
3

3

17
148

0
6

1

5

29
4

2

5

2

3

1

1

2

1

1

2

2

3

5
0

1

3

1

1
9

3

3

1

1

2
0

2

0
3

3

3
26

2

1

2

4

1

7

6

10
0

2

1

5

2

15
2

4

7

1

1

6
0

3

3

1
20

11

4

1

1

1

1

0
2

0
2

2

2
0

0
2

0
2

2

3
0

0
3

3
0

3

2450
68212

2
40

4
0

4

0
3

3

3
5

1

1

3
26

2

1

2

2

4

1

6

1

1

1

2

0
2

2

0
3

3

5
93

9
1

3

5

0
3

3

40
17

12

7

4

4
0

4

0
1

1

31
4

3

6

6

9

1

2

0
6

0
6

2

4

0
45

42
17

1

1

1

15

2

5

1
0

1

1
2

1

0
53

15
53

4

1

5

2

3

2

5

5

1

4

4

2

1
0

1
0

1

5
0

5
1

4

0
7

0
7

1

2

4

1
21

0
2

1

1

2
18

4

2

5

5

6
0

6
5

1

2
0

2
0

2

2
0

2
0

1

1

7
0

0
2

2

0
1

1

0
4

4

1852
553

10
46

5

1

6

1

6

17

0
4

4

0
6

2

1

1

1

1

3
0

2

1

8
0

3

4

1

24
3

1

1

1

1

4

4

2

1

1

4

1

2
15

2

2

2

1

3

3

4
13

5

4

1
0

1

1062
436

3

1

530

3

6

2

3

64

3

2

7

2

115
36

1

2

1

3

2

3

5

1

10

2

1

1

2

5

5

1

2

1

1

1

2

1

2

1

2

14

1

1

1

1

3

0
2

2

5
0

0
2

2

0
1

1

2
0

2

11
179

0
6

1

3

2

3
0

2

1

0
5

5

5
3

2

52
146

1

1

1

13

1

4

1

2

3

1

1

6

2

1

1

1

1

4

3

1

1

26

1

10

1

1

1

2

2

0
3

3

0
1

1

0
25

2
0

2

0
12

4

5

1

2

3
1

1

1

0
8

8

2
0

0
2

2

3
0

2
0

2

1
0

1

9261
460

0
1

1

261
00

14

8

41

0
2

2

12
4

1

2

1

4

0
5

3

1

1

834
422

17

1

11

4

2

2

3

2

19

7

3

1

6

1

9

7

1

7

4

5

12

7

69

2

1

1

2

5

4

1

3

11

2

2

7

2

3

10

6

3

4

5

20

1

4

1

1

2

5

1

7

4

21

1

41

5

2

3

2

4

13

0
3

3
0

1

2

4
0

0
4

4

0
3

3

35
3

19
0

7

3

4

1

4

3
0

3

1
5

3

1

5
0

5

23
0

0
3

3

1
9

2

1

3

2

3
0

3

1

0
7

4

1

2

370
32221

30921
20121

48

12

4

5

16

114

21

4

4

14

2

6

12

3

5

1

15

13

34

30

1

20

671

8

12

5

15
2

1

1

6

1

4

78
17

16

19

26

2
285

0
7

0
7

6

1

0
11

11
5

5

1

0
7

1
0

1

0
3

3

3
0

1

2

13
206

1
0

1

6
3

1

2

1
2

1

0
4

4

6
0

6

3
0

2

1

3
2

1

0
3

3

0
6

6

0
2

2

4
0

4

1
0

1

123
78

5

4

7

3

3

23

0
4

4

0
21

21

3

0
1

1

0
2

2
0

2

0
3

0
2

2

0
1

1

2
47

10
29

1

4

2

3

2

1

2

1

3

4
0

4

12
5

1

1

5

0
2

2
0

0
2

2

0
1

1
0

1
0

1

2
0

0
2

0
1

1

1
0

1

0
1

1
0

0
1

1

281
00

121
00

30
51

1

1

1

5
7

1

1

15
0

4
3

1

6
11

1

1

1

1

1

0
1

0
1

1

0
1861

30
1861

1
0

1

20
88

7

17

1

5

3

1

4

30

605
1718

52

18

6

8

29

106

21

10

8

5

12

20

10

88

10

28

34

41

7

11

9

58

19

27

110

222

16

24

15

17

72

2
8

1

5

1
16

2

5

3

2

3

600
47644

1
0

0
1

1

2
75

7
0

4
0

4

3
0

3

31
0

1
0

1

0
28

28

0
2

2

0
7

1
0

1

6
1

2

2

1

18
0

0
2

2

1
3

2

1
13

12

0
10

3
0

3

7
0

4

1

2

1
0

1

1720
40423

63
542

22
3

5

3

2

2

1

1

5

1
0

1

7
0

2

5

0
9

9

2
0

2

0
6

1

5

1
2

1

44
2

38

4

6
9

1

1

1

0
2

2

28
67

3

3

8

2

6

1

1

1

5

1

8

57
308

3

99

37

80

5

4

4

6

6

6

1

38
2109

1
0

1

78
24

1

1

3

1

1

4

4

4

3

1

24

1

3

3

3
9

1

1

1

1

1

1

1
0

1

0
31

31

179
33

1

1

7

3

4

1

2

1

5

1

1

3

92

1

2

1

14

3

1

1

1

36
9

2

1

1

3

2

1

3

2

1

1

1

3

1

1

2

2

1
5

4

2
6

2

1

1

5
0

5

1704
476

13

255

238

12

1

15

38

637

19

2
16

3

1

1

1

3

1

2

1

1

1
0

1
0

1

0
2

2

14
0

3

11

0
3

3

7201
1060

260
2171

2

2

6

33

21

5

4

5

10

5

5

4

108

3
4

1

0
15

2

1

4

3

3

1

1

0
9

7

1

1

3
0

3

3
0

2

1

0
1

1

38
9

1

4

3

2

2

3

3

5

5

1

1
11

7

3

74
83

4

1

3

1

74
42

4

1

2

5

1

1

2

2

1

1

9

2

1

1
0

1

5
0

5

13
1

3

4

2

3

5
0

5

0
1

1

1
0

1

96
8

2

40

1

2

2

1

1

2

1

7

5

2

20

1

1

1
0

1

0
1

1

3
0

1

2

0
2

2

27
7

4

1

6

2

1

2

1

2

1

382
17

0
3

3

7
2

3

1

1

14
0

14

0
3

3

0
140

139

1

170
1

5

163

1

6
23

4

1

3

2

1

4

2

0
5

5

5
0

4

1

5
0

5

4
0

4

0
1

1

822
40

271
100

2

4

3

1

3

2

2

1

4
0

3

1

0
7

6

1

1
0

1

0
5

5

0
1

1

101
20

1

3

4

1

21
3

1

3

2

5

1

2

1

3

2
0

2

45
0

22
1

9
1

2

1

3

2

5
0

5

2
0

2

0
5

5

1
0

1
0

1

1
8

0
1

1

0
4

1

3

0
1

1

0
1

1

14
0

0
1

1

0
1

1

0
3

3

3
9

1

2

3

10
5391

11
1

5
1

3

1

1
0

1

3
4

1

523
66

16
0

16

0
2

1

1

0
1

1

0
1

1

1
0

1

0
1

1

8
0

1

3

4

0
2

2

0
2

2

420
172

12

1

1

10

9

2

2

17

19

137

2

5

3

3

1

24

3
0

3

00
41

1
0

1

3
0

3

0
1

1

1
0

1

0
6

6
0

0
6

0
6

6

2408717
3380

4012
20

1
0

1
0

1

00
2441

1650
2441

2

2

8

1

4

1

1

1

2

1

19

1

2

1

2

1

14

1

1

3

11

1

25
0

23
12

2

1

1

1

1

1

1

1

1

1

2
0

2

12
0

0
3

2

1

2
0

2

7
0

2

5

1161
00

0
2

1

1

1
0

1

0
1

1

1
113

2

1

1

108

1
0

1
0

1

0
10

0
10

1
0

1

0
3

1

1

1

3
0

3

0
3

2

1

3

0
15640

15640
134

7067
2085

4532

6

105

1

2

97

1

222

14

1

1

256
66

2

66

4

2

25

24

6

1

2

1

11

2

1

1

4

1

1

1

5

4

26

8182
3910

28

2

1

3

2

1

5

1

4

3

2

2

16

16

37

3

3

1

267

1

18

1

1

1

31

2

6

3

9

1

7

11

2

1

7

2

32

37

2

8

2

35

1

8

14

2

4

46

2

5

1

2364

11

119

34

1

33

5

1

29

51

3

858

4

1

1

8

1

38

6

3

1

0
1

1

11009
300

0
6

1
0

1

5
2

1

1

1

00
232

15
3

10

1

1

82
10

1

1

3

12

1

0
62

0
62

61

1

29
0

4
0

1

3

7
12

2

2

1

0
3

3

10
0

10

0
2

2
1

1

00
362

5
0

3

2

0
4

4

130
262

1

1

1

1

1

1

11

6

1

0
1

1

1260
6833

0
13

13

0
5

5

0
2

2

96
122

3

4

3

3

8

4

1

13
21

3

1

1

2

1

3

18
14

1

3

2
0

2

2
0

2

1

213
140

1

1

70

1

4
7

3

0
1

1

1
0

1

40
2

36

2

1

0
1

1

1
3

1

1

1

1
0

1

11

0
3

3

9
3

3

3

0
5

3

2

83
34

1

8

8

4

23

1

4

2282
50

3
0

3

8
0

8

0
12

12

161
31

1

1

1

11

60

2

33

1

1

5

13

1

361
100

6

1

2

131

2

1

1

00
31

3

1

1
0

0
1

1

24
0

24
0

24
0

24

1
0

1

0
22

6
0

1
6

1

1

2

1

11
0

0
2

1

1

3
4

1

0
4

4

1
0

1

0
5

4
0

4

0
1

1

40
36464

11
0

4
11

1

3

1

1

1

780
36314

42
139

89

1

2

3

2

1
0

1

3
0

1

2

1
0

1

33963
21011

75

1

1

1

2

21

20

1

5

4

2

1

1

6

1

17

2

3

2

1

1

1

14

3

2

1

1

1

1

2

2

2

3

7

8

2

6

1

2

1

13

3

14

1

2

3

34

2

2

20

6

2

2

4

22

9

14

1

4

2

4

3

1

125

1

2

1

1

1

1

3

38

2

4

2

2

12

6

7

26

1

8

42

1

1

2

2

1

2

2

2

1

11

3

4

1

1

1

1

1

6

2

1

1

1

8

1

1

1

3

2

1

9

30

55

25

5

2

3

3

2

2

3

3

2

4

15

1

5

1

2

3

6

1

1

3

1

32

1

2

2

1

4

1

56

1

1

3

3

5

1

3

1

2

8

1

5

2

3

4

1

2

1

5

1

4

3

4

1

1

9

3

3

4

2

3

1

20

6

1

1

1

2

4

1

4

1

1

4

12

5

1

1

1

1

1

3

30

1

2

7

4

1

2

18

3

5

1

3

1

3

2

4

1

4

1

1

2

1

3

1

1

4

2

2

2

13
0

1

2

10

0
1

1

134
0

134
0

131
86

6

1

1

12

1

3

1

1

2

1

11

2

3

1
0

1

1

0
1

1

465
3

97
432

252
76

2

2

6

6

30

2

1

1

2

1

13

4

3

4

2

12

11

4

2

1

3

3

9

1

23

17

10

1

0
3

3

0
3

1

2

5
9

3

1

0
1

1

1

1
0

1

1
65

4

60

0
1

0
1

1

0
4

4
0

1

1

2

0
9

9
0

9

0
6

6
0

6

0
10

0
6

2

3

1

0
1

1

1
0

1

2

1
69

5
0

1
0

1

0
2

2

0
1

1

0
1

1

1
45

1
0

1

0
2

1

1

0
1

1

1
0

1

0
1

1

0
11

11

6
27

5

15

1

15
0

0
12

1

11

1
0

1

0
1

1

1
0

1

1
0

0
1

1

1
0

0
1

1

0
1

0
1

1

0
9

0
9

9
2

2

5

1036
28

818
89

163
28

1

99

2

1

2

4

1

1

14

3

1

2

2

2

336
123

7

1

7

1

9

3

6

11

13

4

47

36

1

1

66

43
8

2

4

1

5

5

2

6

1

5

4

3
0

3

5
0

5

6
21

3

4

3

5

39
99

1

1

2

2

1

2

3

2

2

2

4

2

12

1

10

2

2

4

1

2

2

59
14

3

4

30

2

1

2

3

190
24

3
0

3

0
6

4

2

1
0

1

53
113

9

21

16

6

6

2

0
2

2

0
3

2

1

38
6

1

1

7

5

2

3

9

4

2
0

0
2

0
2

2

0
1

0
1

1
0

1

0
1

1

0
9

0
2

2
0

1

1

3
0

1
0

1

0
2

2

0
4

4
1

1

1

1

0
1037

1037
32

59
919

26

50

6

2

6

755

15

13
78

13

2

14

25

3

8

1
0

1

1

0
1

1

2

0
3

2

1

1362
00

1362
30

1
0

1

0
1

1

15
1

3

2

3

1

5

4
0

1

3

1122
290

1

11

11

1

1

2

2

1

2

2

1

1

1

17

1

1

1

1

2

24

2

1

1

1

3

2

3

2

1

1

2

0
3

1
0

1

2
0

0
2

1

1

24998710
740

160
0

3
0

0
3

2
0

2

1
0

1

157
0

3
1

0
2

2

1
154

6
0

1

1

4

2
0

2

145
0

145

74
2

2
0

0
2

0
2

2

10
64

24
0

0
24

2

5

14

3

4
0

4
0

4

11
0

1
11

2

8

0
15

0
9

4

5

6
0

6

6
0

6
0

0
6

6

3
0

3
0

3
0

0
3

2

1

12150
24965310

0
10

10
0

10
0

4

6

2
0

2
0

0
2

2

1261
00

100
1261

1
0

1

2
8

5

1

10
3

1

2

4

12
4

1

2

2

3

0
7

5

2

3
2

1

13
2

2

1

1

2

2

1

1

1

0
2

2

571
201

1

2

1

2

2

1

1

1

1

1

1

3

1

1

1

1

4

3

1

1

1

1

1

1

1

1

1

1
0

1

0
2

2

90
15948

184
15

0
2

1

1

47
85

4

3

3

1

1

2

1

2

1

2

1

2

1

2

3

6

2

1

45
0

45

8
37

1

1

1

2

1

3

1

6

1

1

1

2

2

3

3

0
428

1
0

1

103
427

5

20

4

4

7

89

38

6

1

5

1

1

2

1

4

8

1

1

108

3

10

3

2

0
11

0
11

9

1

1

940
121

2
11

6

3

42
159

3

20

6

4

6

10

9

59

62
228

1

1

1

27

4

2

2

3

3

1

2

1

5

7

1

3

1

3

3

4

1

1

8

3

1

2

1

7

2

13

2

3

1

3

1

1

2

7

5

3

2

3

1

6

1

9

2

67
404

3

2

1

42

3

3

2

3

10

4

1

1

4

127

15

2

2

1

3

4

1

2

2

6

2

2

2

2

4

3

1

2

3

1

1

2

3

1

14

2

2

3

6

3

2

3

1

1

4

3

20

17
3

1

2

6

2

3

0
115

0
115

115

0
1

1

138
0

138
37

2

30

20

4

6

28

6

5

14041
0

14041
2422

9

2

1

488

1

2

38

2

4

109

10

3

6

15

8

49

35

4

28

2

1

5

26

1

2

1

170

5

56

2

1

33

4

1

243

14

3

8

6

739

5

104

1

3

44

4

1

1

19

2

326

3

3

1

2

48

2

3

2

3335

1

2

76

21

4

80

362

121

3

2

14

2

6

3

29

3

5

15

343

4

3

54

38

1

888

36

26

16

186

1

2

3

2

589

1

27

1

7

50

1378

3

21

2

1

835

2

2

5

1

80

4

11

2

41

46

111

1

71581
1770

1049
134

4
1

1

1

1

0
2

2

0
5

5

23
5

3

1

1

5

6

2

0
2

2

0
5

4

1

9
0

5

3

1

2
0

2

6
28

4

2

4

2

6

4

2
0

2

3
18

9

2

1

1

2

3
0

2

1

2
0

2

0
5

5

1
0

1

13
39

3

4

6

1

1

6

2

3

1
0

1

0
5

4

1

23
1

1

3

2

4

1

9

2

14
42

5

12

11

2
0

1

1

10
36

2

11

1

1

7

2

1

1

15
33

2

5

1

2

3

1

1

1

1

1

0
83

83

9
0

8

1

0
2

2

0
6

6

0
3

3

9
17

1

1

3

1

1

1

0
29

1

3

3

1

1

4

6

3

1

3

1

2

458
184

6

6

17

3

3

3

2

11

7

9

3

2

5

4

1

4

3

11

3

2

2

3

1

4

2

1

3

4

3

1

1

2

5

7

2

1

1

2

2

5

4

4

1

8

2

2

3

1

1

2

3

1

3

11

5

3

2

5

2

3

1

2

2

10

2

2

1

5

2

2

2

1

2

3

5

5

1

3

0
8

4

1

3

7
0

2

2

2

1

0
1

1

1
0

1
0

1

0
21

9
2

1

1

5

6
0

1

5

4
1

1

1

1

0
2

2

0
68

0
68

68

2
0

2

0
2

0
1

1

1
0

1

0
13

13
0

3

1

3

6

2
0

0
2

1

1

0
14

3
14

4

1

6

4553
56

3
0

3

8
13

1

2

2

1
0

1

2
15

1

3

4

1

2

1

1

57
798

8

2

6

2

2

319

238

3

161

4
1

3

13
690

32

114

2

241

149

90

49

0
3

3

0
15

4

1

10

15
0

3

12

2
0

2

11
117

2

1

2

3

4

5

27

2

1

1

2

1

2

2

13

3

4

4

1

1

3

4

1

1

1

3

2

1

2

1

1

2

1

2

2815
1933

37

33

5

1

78

37

26

2

39

1

6

592

25

1
0

1

3
5

1

1

101
4

1
0

1

0
16

16

4
75

48

23

0
2

2

3
0

3

1321
50

7
0

2

5

2
0

2

200
1071

24

8

4

2

41

31

14

0
1

1

0
6

6

4
0

4

78
0

68
7

17

1

3

6

3

3

2

1

3

1

3

4

1

3

2

1

3

3

1

1
0

1

0
5

5

0
4

4

0
7

0
6

6

1
0

1

0
591

590
183

1

3

2

1

1

1

49

6

3

144

1

116

1

70

2

3

2

1

1
0

1

5
37

15
3

1

1

2

8

1
17

4

3

1

6

2

0
310

15
158

112

8

23

0
1

1

151
57

4

7

1

32

6

3

2

3

27

4

4

1

20
511

19
1

5
7

2

1
0

1

2
0

1

1

2
8

2

3

1

00
121

0
4

2

2

20
71

11

1

3

1
0

1

0
18

6
13

1

1

1

2

2

2
3

1

2
0

2

0
7

0
7

0
7

5

2

1091
0

1091
8

192
7

3

139

11

2

16

14

20
0

20

25
0

25

1
0

1

1
0

1

0
1

1

814
240

8

34

63

50

1

1

1

2

6

24

8

3

1

196

2

147

10

16

1

2
0

2

0
2

2

14
0

14

7
0

2

1

4

4
0

1

1

2

00
13491

41
13491

0
3

3

0
2

2

49
20

1

1

1

13

2

1

2

2

6

204
1291

283

10

111

683

55
0

55
4

0
22

10

12

6
22

1

1

6

4

4

7
1

1

5

15
0

0
15

2
0

2

13
1

1

4

2

1

4

1
0

0
1

0
1

1

31
221732

221127
57

2
0

1

1

5339
220947

1393

213016

146

17

1036

7
0

3

1

3

5
0

5

0
12

1

1

10

7
25

8

6

2

2

0
37

30

7

1
0

1

31
3

3

17

8

0
3

1

2

19
539

450
94

6

2

4

6

5

1

9

2

7

1

2

10

8

10

22

2

8

8

4

7

4

3

2

102

5

3

4

1

4

1

7

4

2

11

4

3

3

5

1

6

1

4

5

8

19

3

7

1

5

2

2

0
17

17

0
3

3

0
4

4

1

2
38

2

3

1

1

4

5

2

14

1

3

7
0

7

0
3

0
3

3

0
32

6
32

1

10

1

1

3

3

1

3

1

2

5265
00

140
5265

6
0

6

0
9

1

8

1611
4725

1

3

1

2

1

1

1

1

2

2

2

1

1

2

1

17

2

1

1

1

1

1

1

1

1

1

1

1

2

2

3

6

2

3

1

1

1

1

3

15

2

1

1

1

6

7

1

2

1

1

1

2

1

1

1

1

1

1

1

2

1

3

3

1

1

1

1

1

1

1

1

1

1

1

1

2

2

1

3

1

1

1

4

2

2

1

2

1

1

2

1

1

1

1

1

1

1

1

1

1

3

2

1

4

2

2

1

2

1

1

1

1

1

1

2

1

1

1

1

3

2

1

2

3

1

1

1

1

1

1

1

3

1

1

1

1

1

1

3

1

1

1

1

2

1

1

4

2

1

1

2

1

1

2

1

1

2

1

1

1

1

3

1

1

2

1

1

2

1

1

3

1

1

2

1

1

5

1

2

1

1

1

2

1

2

2

3

1

0
1

1

1
0

1

20
3

3

1

2

2

1

1

1

2

1

1

2

3
0

3

8
0

8
0

8
1

4

3

0
9

0
9

1
3

1

1

0
2

2

0
2

2

0
2

2

0
8

8
0

8
0

3

1

2

1

1

3421
00

3421
100

1
11

2

2

6

1
3

2

9
1

3

3

2

0
3

3

2
0

2

13
70

4

1

11

1

11

2

1

21

1

4

1361
50

1

11

1

128

1
0

1

3
0

2

1

13
0

4

3

4

2

0
2

2

15
58

1

1

4

1

1

1

3

2

1

1

3

2

1

2

1

3

1

4

2

3

2

3

18
0

18

3
1

2

0
4

4
0

0
4

4
0

4

19
0

3
19

1
16

0
3

3

4
0

4

6
0

4

2

2
0

2

1888201315059
9308

20
7652

00
7212

7211
100

445
0

445

1
0

1

0
2

2

1771
130

25

3

55

3

21

76

6
78

1

29

42

0
8

8

0
1

1
0

1

1
0

1

41
2

31

8

3
0

3
0

3
2

1

10466630
1887918115039

1876690614995
2364986197

10
4121

254
10

2

6

11

223

2

92
0

22

70

2
0

2

14
2

10

2

71
20

5

1

4
20

16

0
22

22

29987161
1632049314735

8
0

8

1594965914628
81997142265

134

1462

11581

700676711166

472535

2

4366

2892

574

44389

1466317

9144

36

72953614

383515

48

639344488

7095546
27748

18

19

9

15

8

2

2441

3

3

31

90

881

13

46

10001

1

7

5

1

75

117

69

5

1901

1

4

691

2

6

23

96

44

4

3

9

411

15

8581

1

1

1

1

73

5

58

8

9

17

185

107

60

781

3

55

2

13

39

3

1

2

2

2

7

582

10

6

1

533

381

76

174

40

21

15

63

2

561

6

9

330

4

6274825

5

39

8

339217
270

10
1651

17

3

22

12

85

4

21

3

11

3

2

4021
310315

2

1

3

2

16288

1

3

58

2

1

6432

14

2

461

11

2

7

2

4

12

1051

1522

10
971

5

78

131

282
7

1
0

1

3
7

4

2
0

2

78
8

13

20

10

27

0
170

170

0
8

8

0
1

1

0
8

8

7734145
44138

1140
19273

16761

4

1161

6

2

91

8
0

8

1170
15621

28

1

4

1

512

114

7691

3

2

7

4

4
0

2

2

21
0

21

0
27

18

9

1510
50216

1

48135

371

1

18

0
6

6

9
0

9

1360
153605

1

1

43

3031

18

594

2

1

3

9

1

2

2

1

142394

1

3

00
291

8

7

31

8

3

10
1061

951

1

1

8

1946814
00

1942314

10

26

5

2

2

28
14

4

10

90
1

27

62

7
0

7

3
86

4

68

1

1

4

3

1

1

0
4

4

1121
90

1

8

2

64

20

21

6

46
170

55

63

5

1

5
2

2

1

7
4

1

2

53
0

2

7

22

9

6

2

5

287945
23610

1

57

5958

2291

16

44

958

2

1

253

187754

131

8

0
24

6

18

760914
1141

8
0

8
0

4

1

1

2

1641
60

1

0
2

1

1

170
1431

1

1

4

2

1

1

4

1

1

1

1

1

1

6

2

1

3

1

1

401

1

1

1

1

1

13

2

1

1

1

20

1

6

1

1

12
1

3

2

2

1

3

34
109

10

13

16

1

4

7

3

1

9

3

1

3

2

2

261
9715

1
0

1

1

4
0

4

14
5

2

5

2

3

9
2

3

4

1
0

1

10
231

21

1

4

7

1

1

1

5

7
0

7

0
1

1

4123
2021

1

1

1

61

1

1

7

1

20

18

2

13

1

5

5

1

10

2

2

11

24

23

1

36

1

3

1

3

14

2

3

1
0

1

1
3

2

0
4

4

19
50

4

2

1

1

1

2

1

18

1

9
0

9

4
0

1

1

2

0
2

2

2
0

2

174
54

2

111

1

6

0
3

3

0
3

1

1

1

8

9
0

9

30
0

28

1

1

8
3

1

1

3

0
1

1

13
4

1

5

1

1

1

0
3

2

1

57
2

1

1

52

1

3
0

3

2
0

1

1

9
4

2

1

2

0
1

1

12
3

3

5

1

57
58

1

58443
220

10
131

1

10

2

1
30

2

11

15

1

1
0

1

6460
57322

1

679

3

1

9

10

3

7

1

3

19701

359

1

4

22

270

1

1

11

1

606

15

5

2

2

7

2

1

1

6

801

5

1

8

57

2

11

613

2

2

232

65

2

2

18
0

17

1

16
1

15

1
12

11

0
1

1
0

1

14
0

14
0

14

0
49

1
0

1

2
0

2

4
17

2

1

2

2

5

1

1
2

1

0
1

1

0
2

2

2
24

1

3

1

1

6

1

8

1

0
245

7
245

21

23

192

2

0
2

0
2

2

884
00

80
444

1

2

8

2

7

164

0
44

44

0
560

0
538

538
0

535
30

14

3

44

4

6

420

14

1
0

1

2
0

2

0
17

17
0

4
14

1

3

2

3

1

3
0

3

0
5

0
5

3
1

2

0
2

2

52510
230

2
0

0
2

2

603
00

1
0

0
1

1

141
00

111
00

111

3
0

2

1

0
6

6
0

6

362
00

0
1

1

00
281

281

1
0

1

4
0

3

1

21
00

2

1

3
0

1
3

2

0
114

1
114

0
1

1

0
3

3

109
0

109

2
0

2

0
1

1
0

1
0

1

51
00

00
41

0
2

2

00
21

21

1
0

0
1

1

73
0

69
4

0
3

3

3
0

3

0
4

1

1

2

1
0

1

12
0

8

1

1

1

1

0
2

2

0
1

1

0
1

1

1
0

1

0
1

1

3
0

3

2
0

2

0
2

2

0
4

4

2
0

2

3
0

2

1

14
1

3

10

2
0

2

2
0

2

2
0

1

1

0
3

2
3

1

1
0

1
0

1

2456
60

0
4

1
0

1

3
0

3

0
1

0
1

1

15
5

0
10

10

0
5

5
0

5

0
2

0
1

1

0
1

1

1
0

1
0

1

1
0

0
1

1

1
0

1

0
1

1
0

1

0
2

0
2

2

0
3

3

2
66

1
0

1

1
4

2

1

0
1

1

4

4
0

4

1
0

1

11
0

11

0
8

5

3

1
0

1

0
1

1

3
0

3

0
1

1

10
24

1

1

12

3
0

0
3

3

0
1

1
0

1

1
0

1

00
1245

271
1225

2

1

1

2

17

1

1

1

1

1

1

2

51

1

1

1

2

81

1

47

2

9
0

9

2
0

0
2

2
0

2
0

2

47
0

0
47

4
39

7
0

7

0
1

1

1
0

1

2
0

2

23
1

1

14

2

3

1

1

1
0

1

5
0

2
5

2

1

3
0

1
0

1

2
0

2

0
31

31
0

6
0

6
0

0
6

1

1

2

1

1

24
2

16
1

1
0

1

4
14

2

1

1

1

1

3

1

5
0

0
5

1

1

3

1
0

1
0

1

1
0

1
0

0
1

1

00
1132

180
1132

1
0

0
1

0
1

1

3
12

3
0

3
0

1

1

1

0
6

2
0

2

0
4

4

3
0

3
0

3
0

3

1
0

0
1

1

0
24

9
0

0
7

7

0
2

2

2
0

2

0
4

4
2

1

1

0
9

9
7

1

1

0
9

9
0

9
8

1

0
3

1
0

1
0

1

0
2

0
2

2

0
3

0
3

0
3

3

3
0

3
0

0
3

1

2

0
12

10
2

0
8

8

2
0

0
1

1

0
1

1

40
251

1
0

0
1

1

0
8

3

3
0

3

1
0

1

1
0

1

00
91

0
1

1

00
71

1

2

2

1

1

1

1
0

1

3
0

3
0

3

63
2

0
8

8
0

3
0

0
2

1

1

0
1

1

5
0

0
5

5

0
53

3
0

3
0

3
0

3

0
50

12
50

0
8

8

0
1

1

1
0

1

4
0

4

0
1

1

0
5

5

5
0

5

13
0

5

8

0
1

1
0

1
0

1
0

1
0

1

22
9

8
0

0
7

7
0

1
7

5

1

1
0

1
0

0
1

1

2
5

3

0
4

0
4

4
0

0
4

4

0
538

0
477

0
477

477
0

477
0

476

1

0
1

0
1

1
0

0
1

1

20
0

0
20

0
20

20
1

19

7
0

0
7

0
7

7

0
33

0
33

0
33

0
33

33

00
41

00
41

00
31

31
00

31
20

1

1

1
0

0
1

0
1

1

2
0

0
2

0
2

0
2

2
0

2

0
1

0
1

0
1

1
0

0
1

1

531
00

0
31

31
1

1
19

1
0

1

4
10

1

5

7
1

6

4
0

4
2

1

1

7
0

1
4

3

0
2

2

0
1

1

0
2

2
0

0
2

2

0
2

0
2

2
0

2

00
181

0
2

2
0

2

0
4

4
0

4

00
121

2
0

2

21
00

21

2
8

6

0
1

0
1

0
1

0
1

1
0

1

2
0

2
0

2
0

0
2

2
0

2

0
7

7
0

7
0

7
0

2
0

2

3
0

1

2

0
2

2

0
6

0
6

0
6

1
0

0
1

1

0
5

1
4

2

1

1
0

1

4
0

0
4

0
4

4
0

1
0

1

0
3

3

30
3642

331
0

327
0

0
3

1

2

0
324

1

319

1

1

1

1

0
4

2
0

1

1

0
2

1

1

302
00

3
0

3
1

1

0
1

1

191
20

171
00

171
80

1

1

6

1

1

00
81

51
00

51
01

1

1

1

2

0
3

3
0

3

10
0

10
0

0
10

10
0

0
9

9

0
1

1

60
403

20
131

00
71

71
00

0
1

1

1
0

1

6
0

1

5

4
0

3
0

0
3

1

2

0
1

1
0

1

42
00

42
00

0
2

2
0

2

00
22

00
12

1

1

1

0
1

1

0
5

5
0

5
0

0
5

5

0
12

12
0

0
12

0
6

4

1

1

0
3

3

1
0

1

1
0

1

0
1

1

361
00

00
311

12
0

12
0

0
2

2

1
0

1

2
0

2

0
4

4

1
0

1

1
0

1

0
1

1

00
91

91
00

0
2

2

0
1

1

11
00

1

1

2
0

2

0
3

3

2
0

2
0

0
2

2

8
0

0
3

2
0

2

1
0

1

1
5

0
1

1

3
0

3

3
0

3

2
0

0
2

2
0

2
0

2

30
0

15
0

0
15

0
1

0
1

1

14
0

0
4

4

10
0

10

0
6

6
0

6
0

6

9
0

0
9

9
0

9
1

5

2

1

00
1282

1282
00

1282
00

00
1031

1
0

1

240
1021

2

2

11

4

49

41

4

1

1

00
251

241
40

2

11

2

1

2

1

2

5

1

3

0
1

1

337714
520

00
24999

24999
220

0
7

1
7

6

70
2278

1
0

1

2
0

1

1

7
0

4

1

2

00
11

11

0
1

1

0
1

1

1
0

1

753
221

2

1

1

1

1

1

2

1

1

1

1

1

11

7

1

1

1

1

1

2

1

1

1

1

2

1

1

5

1

3

1

2

1

3

1

0
2

1

1

0
2

1

1

3
11

1

7

1

181
90

2

2

1

1

1

2

1

3
0

3

0
1

1

2
0

2

2
0

1

1

90
601

6

1

6

1

21

11

1

3

4

1

3

3

1
11

1

4

5

1
0

1

1
0

1

1
0

1

0
1

1

0
1

1

0
1

1

0
6

3

1

2

1
0

1

0
2

2

0
1

1

2
0

2

1

1
0

1

2
0

2

2
0

0
2

2

1260
22391

44
701

1

1

7

3

1

3

1

2

3

1

1

2

19

13

2

1

3

2

1

2

1

3

2

9

1

1

1

1

551

1

1

9

2

1

2

2

71
0

6

2

1

6

56

1
0

1

32
8

6

2

16

2
0

2

12451
170

11971

31

28
0

2

5

1

17

3

1
0

1

32
10

15

2

5

0
112

0
112

3
110

5
2

1

1

1

0
12

2

2

1

4

2

1

90
16

36

2

12

3

1

1

3

1

13

1

1

2
0

0
2

2

10
4713

4612
130

0
23

0
3

1

1

1

0
20

16

4

3502
60

0
1

1

0
24

21

3

40
274

15

10

15

29

1

1

18

89

1

9

6

25

6

7

2

452
41

18

211

2

3
0

1
0

1

2
1

1

0
4

4
0

2

2

0
7

7
0

1

6

1
0

0
1

1

1
18

11
3

1

1

1

1

1

1

1

1

0
6

1

1

4

42
0

1
0

1

41
4

2

6

7

4

2

16

00
91

3
0

3
0

3

00
61

1
0

1

2
0

2

00
21

21

0
1

1

11
00

00
11

0
1

0
1

1

0
1

1

481
00

481
00

481
20

23
33

1

1

2

1

4

1

1
0

1

0
3

3

1
0

1

00
11

11

5
0

5

0
1

1

0
1

1

0
194

194
4

3
0

3
0

3

1
0

0
1

1

1
0

1

3
0

0
3

1

1

1

0
7

2
6

1

1

2

1
0

1

9
142

1
0

1

2
1

1

15
68

2

1

2

1

2

4

1

4

8

3

1

1

3

6

2

1

4

1

2

4

60
0

60

0
2

1

1

0
2

2

0
3

3
2

1

0
4

0
1

1

1

2
0

2

1
21

3
0

3

14
3

5

1

1

1

3

1
0

1

2
0

2

3
0

3
0

2

1

3
0

0
3

3
0

0
3

3
0

3

3
0

0
3

3
0

3
0

0
3

3

10
321

0
1

1

00
71

1
0

1
0

0
1

1

00
61

61
00

0
2

2

00
31

11

1

1

0
1

1

0
14

14
1

1
13

0
3

1

1

1

6
0

1

5

2
1

1

1
0

1

9
0

0
9

0
9

1
9

2

6
