# Supplementary material for: Development and validation of a LAMP assay for detection of genetically diverse Lactococcus c2-type bacteriophages in dairy environments
Source: Front Microbiol. 2026 Aug 10;17:1912103. doi: 10.3389/fmicb.2026.1912103 (PMC13500662; doi:10.3389/fmicb.2026.1912103)
Supplement: Supplementary file 2 [file Data_Sheet_1.ZIP › Supplementary File S1/L.lactis_637_4_krona.html]

Javascript must be enabled to view this page.

members
magnitude
magnitudeUnassigned
count
unassigned
taxon
rank

117.kraken2


 99.49 99.40 0.00 0.00
4
1879
2308
3197121

16335
1

 0.51

2
1
1709

 99.21
18408
superkingdom
3171771

11
phylum
0
203691
22
1

 0.00

11
0
class

 0.00
1
21
203692

136
12
1

 0.00
0
order
9

2
0
family
2791015

 0.00
1
2

2
genus

 0.00
1
399320

1
3

 0.00
1643685
1
family
0

1

 0.00
2
64895
1
0
genus

1
species
42109

 0.00
1

6
0
family
6
1

 0.00
137

2
0
genus

 0.00
1
2
1616789

1

 0.00
1307761
2
species

146

 0.00
1
3
0
genus
4

4
species
0

 0.00
1
2
46355

1

 0.00
889378
strain
4


 0.00
1
8
1643688
0
order
2


 0.00
1
7
170
2
family
0

171
1

 0.00
3
genus
0
1

0
no rank
1
1
2

 0.00
2633828

1513297
1

 0.00
species
1

338321

 0.00
1
3
genus
0
1

1
species
0

 0.00
1
2
29510

strain
1
869212

 0.00
1

4
0
phylum
9
1

 0.00
1297

188787
1

 0.00
8
2
class
4

118964
1

 0.00
7
order
0
2

2
family
0
183710
1
6

 0.00

1298
1
5

 0.00
0
genus
2

1
0
no rank
1

 0.00
2
2623546

1
species
1

 0.00
3093660

2
1

 0.00
502394
species
0
1

745776
1

 0.00
strain
1

clade
0
159
1783270
1

 0.00
130

159
clade
0

 0.00
1
129
68336

1
6

 0.00
1853220
0
phylum
3

1853222

 0.00
1
5
class
0
3


 0.00
1
4
1853224
order
0
3

0
family
3
3
1

 0.00
1853225

0
genus
3
2
1

 0.00
146918

146919
1

 0.00
3
species

phylum
5
155
976
1
115

 0.00

117743
1
42

 0.00
30
class
0

200644
41
1

 0.00
30
order
3

1853230

 0.00
1
4
1
family
0

0
genus
1
332102

 0.00
1
3

species
0
1
191579
1
2

 0.00

strain
1

 0.00
1
755732

39782

 0.00
1
3
0
family
1

1
2

 0.00
34098
0
genus
1

species
1
1

 0.00
1653831


 0.00
1
15
49546
11
0
family

0
genus
1
104264

 0.00
1
2


 0.00
1
979
species
1


 0.00
1
3
417127
genus
0
1

2632541

 0.00
1
2
no rank
0
1

1
species
1

 0.00
2779359

252356
1

 0.00
3
1
genus
0

2615042
2
1

 0.00
no rank
0
1

species
1
3053613

 0.00
1

genus
1

 0.00
1
143222

genus
0
1
444459
1

 0.00
3

0
no rank
1
1

 0.00
2
2644544

1
species
1

 0.00
2654676

237
2
1

 0.00
4
genus
6


 0.00
1
996
species
2

no rank
0
3
313602
7
1

 0.00


 0.00
1
2
336809
genus
0
1

336810

 0.00
1
species
1

genus
0
2
1940138
1
4

 0.00

1
no rank
0
1

 0.00
2
3066218


 0.00
1
3066219
1
species

1

 0.00
1415657
1
species

family
1
11
2762318
11
1

 0.00

0
genus
1
1

 0.00
2
1778601

2500547

 0.00
1
species
1

no rank
0
9
2782232

 0.00
1
8

8
0
genus
59732

 0.00
1
5

species
6
373672
1

 0.00

species
1

 0.00
1
3042315

0
no rank
2593645
1

 0.00

1
species

 0.00
1
1241978

1
0
genus
2782229
1

 0.00
2

1
species

 0.00
1
2487072

1853228
1
10

 0.00
4
class
0

4
order
0
1
9

 0.00
1853229

0
family
4
8
1

 0.00
563835

genus
0
2
2836216
2
1

 0.00

1

 0.00
2676868
2
species

genus
0
1
2812019
2
1

 0.00

1

 0.00
2502779
1
species

1649506
3
1

 0.00
1
genus
0

1

 0.00
2
3099314
1
no rank
0

1968839

 0.00
1
1
species

3
0
class
117747
5
1

 0.00

3
0
order
200666
1

 0.00
4

1

 0.00
3
84566
family
0
3

1
genus
423349
1

 0.00

2
genus
28453

 0.00
1

39
class
0
1
28

 0.00
200643

37
order
0
171549
1
22

 0.00

1970190

 0.00
1
3
family
0
1

1970191
1

 0.00
2
genus
0
1

1
species
1

 0.00
1307839

24
family
31
1

 0.00
8
171552

genus
0
7
838
1
7

 0.00

no rank
0
2
3
1

 0.00
2638335

species
1
712471
1

 0.00

1
species
1

 0.00
2691580

1

 0.00
28133
1
species

species
3

 0.00
1
28131

28132

 0.00
1
1
species

1

 0.00
3
1853231
2
family
0


 0.00
1
2
283168
2
0
genus

species
2
28118

 0.00
1


 0.00
1
4
2005520
0
family
2

307628
3
1

 0.00
0
genus
2

2
no rank
0
2646834

 0.00
1
2


 0.00
1
2023866
2
species

2760872

 0.00
1
3
0
family
1

1

 0.00
2
2760873
1
0
genus

species
1
2590900
1

 0.00

1970189
1

 0.00
5
order
0
2

1
4

 0.00
558415
2
family
0

2
genus
0
1

 0.00
3
1628153

2
1

 0.00
1628155
2
no rank
0

2
species
1628156
1

 0.00

1

 0.00
29
768503
class
0
74

74
order
0
768507

 0.00
1
28

family
7
8
3
1

 0.00
200667

59739
1
2

 0.00
1
genus
0

species
1
367791
1

 0.00

89373
1
3

 0.00
0
family
2

0
genus
2
1

 0.00
2
107

2666025
1

 0.00
2
species

64
family
1
21
1

 0.00
1853232

1

 0.00
18
89966
61
6
genus

1

 0.00
2932251
1
species

2
species
497967

 0.00
1

species
1

 0.00
1
2496028

2761579

 0.00
1
species
1

species
3
1850093
1

 0.00

3
species

 0.00
1
1385715

40
no rank
1
2615202
1
10

 0.00

6
species
1

 0.00
1484118

2596915
1

 0.00
1
species

species
3
2932250

 0.00
1

species
22

 0.00
1
2675878

1
species
1

 0.00
2987689

2675877
1

 0.00
1
species

species
2
2932253

 0.00
1

species
1
1

 0.00
2835648

2
species
3093922
1

 0.00

1

 0.00
1411621
species
4

1
genus
2
323449
1

 0.00
2

2592049
1

 0.00
species
1

1134404
1
7

 0.00
1
phylum
0

1
0
class
795747
6
1

 0.00

1
0
order
1

 0.00
5
795748

1334117
1

 0.00
4
0
family
1

1134403
1

 0.00
3
1
0
genus

1
2

 0.00
1134405
0
species
1

1191523

 0.00
1
strain
1

2138240
7
1

 0.00
3
phylum
0

6
1

 0.00
2138243
0
class
3


 0.00
1
5
2138246
0
order
3

2138247
1

 0.00
4
3
0
family

genus
0
3
68335

 0.00
1
3

0
species
3
1
2

 0.00
35786


 0.00
1
309798
3
strain

1

 0.00
20
3018035
35
0
phylum

0
class
3
3031419
1
5

 0.00

1

 0.00
4
2024979
3
0
order

263369
3
1

 0.00
0
family
3

3
genus
0
263370
2
1

 0.00

3
species
1

 0.00
28202

7
1

 0.00
1553900
class
0
30

1

 0.00
6
2024973
30
0
order

2024974
1
5

 0.00
30
0
family

3
0
genus
2024975
2
1

 0.00

1915309
1

 0.00
species
3

0
genus
27
2698753

 0.00
1
2

1

 0.00
2493639
species
27

class
0
2
3031418
7
1

 0.00

order
0
2
6
1

 0.00
213481

family
0
2
1

 0.00
5
213483

958

 0.00
1
2
1
genus
0

species
1
1

 0.00
959

213485
1
2

 0.00
0
genus
1

349221

 0.00
1
species
1

13
1

 0.00
200783
3
phylum
0

1
12

 0.00
187857
3
class
0

0
order
1
1485951
1
2

 0.00

family
1
1

 0.00
558314

32069
9
1

 0.00
0
order
2

224027
4
1

 0.00
1
family
0

182899
1

 0.00
3
1
0
genus

1
0
species

 0.00
1
2
309805

1
strain
1

 0.00
123214

1
0
family
64898

 0.00
1
4


 0.00
1
3
2713
0
genus
1

species
0
1
63363
1

 0.00
2

224324

 0.00
1
1
strain

1224

 0.10
1
365
3181
phylum
10

191
1

 0.10
1236
class
19
3040


 0.00
1
3
118884
0
no rank
1

198346

 0.00
1
2
1
0
genus

186490

 0.00
1
species
1

135624
6
1

 0.00
order
0
4

4
0
family
84642
1
5

 0.00

genus
0
4
642

 0.00
1
4

1

 0.00
645
species
2

1
species
1

 0.00
654

1
species
1

 0.00
648

2887326

 0.02
1
28
525
order
0

525
0
family
1

 0.02
27
468

1

 0.00
3
497
0
genus
1

0
no rank
1
196806

 0.00
1
2

species
1
1

 0.00
3064892

137
genus
33
469

 0.00
1
20

1

 0.00
2
909768
3
species group
0

1

 0.00
470
species
3

2053287
1

 0.00
1
species

species
2
108981
1

 0.00


 0.00
1
28090
47
species

species
38
40214
1

 0.00

1
species
1879050
1

 0.00

108980

 0.00
1
species
2


 0.00
1
9
196816
no rank
0
9

species
1
2004644
1

 0.00

species
1
1

 0.00
2714110

1
species
3120518
1

 0.00

1636603
1

 0.00
1
species

species
1

 0.00
1
1758189

2004646
1

 0.00
species
1

1
species

 0.00
1
2743575

2725684

 0.00
1
2
species

1

 0.00
2
40216
species
0
1

1
strain

 0.00
1
981334

475

 0.01
1
3
0
genus
387

1
species
90241
1

 0.00

34062
1

 0.01
386
species

1

 0.00
12
135622
9
0
order

8
family
0
1

 0.00
7
267890

22

 0.00
1
6
8
genus
1

species
3

 0.00
1
256839

species
1
2487742

 0.00
1


 0.00
1
332186
2
species

2
1

 0.00
196818
1
no rank
0

1
species
351745
1

 0.00

family
0
1
1

 0.00
4
267889

1
0
genus
1
3

 0.00
1518149

2614972
1

 0.00
2
1
0
no rank


 0.00
1
2552945
1
species

72273
6
1

 0.00
1
order
0

1

 0.00
5
34064
family
0
1

262

 0.00
1
4
1
genus
0

263

 0.00
1
3
1
0
species

119857

 0.00
1
2
1
subspecies
0


 0.00
1
393011
1
strain

1
order
0
1
3

 0.00
1706369


 0.00
1
2
1706373
1
family
0

48073
1

 0.00
genus
1

72274
26
1

 0.00
35
order
0

135621
1
25

 0.00
0
family
35

2901164

 0.00
1
4
genus
0
1

136846
3
1

 0.00
1
species group
0

1

 0.00
2
578833
species subgroup
0
1


 0.00
1
203192
1
species

2
genus
34
286

 0.00
1
20

1

 0.00
2
136841
species group
0
2

287
1

 0.00
2
species


 0.00
1
2993566
1
species

species group
0
3
1

 0.00
2
136845

1

 0.00
47885
3
species

136849
3
1

 0.00
1
0
species group

0
species subgroup
1
1
2

 0.00
251695

species
1

 0.00
1
317


 0.00
1
9
196821
24
no rank
7

species
1
2025658

 0.00
1

10
species

 0.00
1
2855434

2973088
1

 0.00
species
1

1
species
2749808
1

 0.00

1
species
2697023

 0.00
1


 0.00
1
2762896
1
species

1
species

 0.00
1
2605424

3016341
1

 0.00
species
1

0
species group
1
136842

 0.00
1
2

species
1
587753
1

 0.00

1
11

 0.00
135625
0
order
4

family
0
4
712
1
10

 0.00

genus
0
1

 0.00
1
4
745

1
0
species
747

 0.00
1
3

44283
2
1

 0.00
1
subspecies
0

strain
1

 0.00
1
272843


 0.00
1
2
416916
1
genus
0

1
species
714

 0.00
1

724
1
3

 0.00
0
genus
2


 0.00
1
729
species
1

1

 0.00
727
1
species

52
0
order
135623
1

 0.00
13

52
0
family

 0.00
1
12
641

2
0
genus
657
3
1

 0.00

2
1

 0.00
38293
2
species
0

1

 0.00
85581
2
subspecies

50
genus
2
662
1
8

 0.00

2
1

 0.00
2614977
0
no rank
1

2819096

 0.00
1
1
species

1
species

 0.00
1
990268

666

 0.00
1
species
3

0
species group
43
717610
1

 0.00
3

670
1

 0.00
41
species


 0.00
1
669
2
species

2377
order
2
72
1

 0.07
91347

1903409

 0.00
1
6
0
family
2

32199
1

 0.00
3
genus
0
1

species
0
1
1
2

 0.00
9


 0.00
1
3075418
forma specialis
1

genus
0
1
1
2

 0.00
53335

470931

 0.00
1
species
1

2300
0
family
1903414
1

 0.07
15

0
genus
2
4
1

 0.00
637

1
species
1

 0.00
638


 0.00
1
2
2627083
no rank
0
1

species
1
1231049

 0.00
1

0
genus
2292
1
3

 0.07
626

2292
species
0

 0.07
1
2
40576

strain
2292
1

 0.07
406818

1
genus
0
2
1

 0.00
583

584

 0.00
1
species
1

586
3
1

 0.00
0
genus
1

516075
1

 0.00
2
1
species
0

1141660

 0.00
1
1
strain

4
genus
0
581
1

 0.00
2

1

 0.00
582
4
species

67
family
3
1
36

 0.00
543


 0.00
1
2
2055880
3
0
genus

species
3
1

 0.00
566

4
1

 0.00
544
genus
0
4

species
3
1

 0.00
2971264

2
1

 0.00
1344959
1
species group
0

1

 0.00
546
species
1

1
no rank
0
1
2

 0.00
36866

species
1

 0.00
1
2675791

0
genus
21
4
1

 0.00
561

21
17
species

 0.00
1
3
562

1
strain
1

 0.00
316435

1

 0.00
3038394
no rank
3

no rank
1
25
2890311
6
1

 0.00

24
15
genus
570

 0.00
1
5


 0.00
1
2058152
4
species

species
1
548
1

 0.00

573

 0.00
1
2
3
species
4

1
subspecies
1

 0.00
72407

5
genus
2
1
5

 0.00
547

1

 0.00
881260
1
species

354276
1

 0.00
3
0
species group
2

species
1
299767
1

 0.00

1
species
550

 0.00
1

0
genus
2

 0.00
1
2
620

2
species
1

 0.00
623

590
1
4

 0.00
1
0
genus

28901

 0.00
1
3
0
species
1

2
1

 0.00
59201
subspecies
0
1

1

 0.00
29474
1
no rank

191675
1

 0.00
4
no rank
0
1

3
1

 0.00
84563
1
0
clade

0
genus
1
1906659
1
2

 0.00

1
species

 0.00
1
1778263

0
genus
1
83654
2
1

 0.00

1
species

 0.00
1
83655

2812006
4
1

 0.00
1
family
0

84565
1
3

 0.00
genus
0
1

1
0
no rank
2636512
2
1

 0.00

1929246
1

 0.00
species
1

2
family
0
4
1

 0.00
1903410

2
genus
0

 0.00
1
3
122277

1

 0.00
180957
1
species

1

 0.00
1905730
1
species

3
family
0
1
6

 0.00
1903411

613
5
1

 0.00
genus
0
3

3
1

 0.00
2647522
2
no rank
0

1
species
2785630

 0.00
1

1
species
1

 0.00
2986763

1

 0.00
138074
1
species

135614
10
1

 0.00
12
0
order

12
0
family

 0.00
1
9
32033

0
genus
10
338

 0.00
1
4

339
1

 0.00
4
species

2
species

 0.00
1
2775159

species
4
29447

 0.00
1

2
0
genus
1
4

 0.00
40323

1
0
no rank
1

 0.00
2
196198

1
species

 0.00
1
2691571

3003272
1

 0.00
species
1

1
class
65
28216
62
1

 0.00

32003
1

 0.00
4
0
order
1

0
family
1
2008793
1

 0.00
3

2
1

 0.00
1914449
1
genus
0

1
species
1565605

 0.00
1

1442155
1

 0.00
4
1
0
order

1
3

 0.00
1442156
1
0
family

1

 0.00
2
416212
1
0
genus

species
1
1

 0.00
416213

41
order
1

 0.00
1
39
80840

0
family
26
13
1

 0.00
75682

29580
1

 0.00
2
genus
0
1

29581
1

 0.00
species
1

1
0
genus
963
1

 0.00
3

341045
1

 0.00
2
0
species
1

1
strain
1

 0.00
1262470

2895353
1

 0.00
7
0
no rank
24

2
genus
0

 0.00
1
2
34069

1

 0.00
3068633
species
2

149698
4
1

 0.00
0
genus
22

945844

 0.00
1
5
species

2
1

 0.00
2609279
no rank
0
17

1

 0.00
2861282
17
species

80864
1
8

 0.00
5
0
family

1

 0.00
2
47420
genus
1
2

2610897
1

 0.00
1
no rank

0
genus
1
1

 0.00
2
283

1

 0.00
225991
1
species

80865
1

 0.00
genus
1

34072
1

 0.00
2
genus
0
1

1
no rank
1

 0.00
663243

0
no rank
1
224471
1

 0.00
3

genus
0
1
92793
2
1

 0.00

species
1
1296669
1

 0.00

4
0
family

 0.00
1
8
119060

2
0
genus
32008

 0.00
1
4

3
1

 0.00
111527
2
species group
0

57975
1

 0.00
2
2
0
species

1

 0.00
271848
strain
2

3
1

 0.00
1822464
2
1
genus

2615204
2
1

 0.00
no rank
0
1

1

 0.00
3141544
1
species

4
0
family
506

 0.00
1
6

29574
1
3

 0.00
genus
0
3

29575
1

 0.00
2
3
0
species

1

 0.00
743973
strain
3

1
0
genus
1

 0.00
2
517

35814

 0.00
1
1
species

0
no rank
1
119066
3
1

 0.00

1301080
1

 0.00
2
1
0
genus

1160784
1

 0.00
1
species

order
0
20
206351
11
1

 0.00


 0.00
1
4
2897176
family
0
1

168470
1
3

 0.00
0
genus
1

0
species
1
168471
1
2

 0.00

strain
1
557598
1

 0.00

0
family
19
6
1

 0.00
481

0
genus
18
1
2

 0.00
2593687

18
species
2067065
1

 0.00

0
genus
1
482
1
3

 0.00

0
species
1
496
1

 0.00
2

strain
1
997348
1

 0.00


 0.00
1
107
28211
65
class
1

1

 0.00
5
3119832
0
order
1

3119833

 0.00
1
4
0
family
1

1
genus
0
767891

 0.00
1
3

1
species
0

 0.00
1
2
767892

488538
1

 0.00
strain
1

204455
1
13

 0.00
10
0
order

family
0
2
2854170
1
2

 0.00

60136
1

 0.00
2
genus

8
1
family

 0.00
1
10
31989

6
1
genus
265
6
1

 0.00

species
2
59779

 0.00
1

1
4

 0.00
2688777
3
no rank
0


 0.00
1
2760307
species
1

1
species
1

 0.00
2895796

2589076
1

 0.00
1
species

1
0
genus
991903
3
1

 0.00

1
species
0
2
1

 0.00
991904

1
strain
991905

 0.00
1

0
order
4
766

 0.00
1
8

1328881

 0.00
1
family
1

942

 0.00
1
6
3
family
0

952

 0.00
1
5
3
tribe
0

3
0
genus

 0.00
1
4
953

1
3

 0.00
2640676
3
2
no rank

2
1

 0.00
77038
species
0
1

1236909

 0.00
1
strain
1

204441
11
1

 0.00
0
order
4

41295

 0.00
1
4
family
0
2

2
genus
0
3
1

 0.00
1612157

0
species
2
1084
1

 0.00
2

strain
2

 0.00
1
1150469

1

 0.00
3
2829815
family
0
1

191
1

 0.00
2
1
0
genus

1064539

 0.00
1
1
species


 0.00
1
3
433
0
family
1

genus
0
1
125216
1

 0.00
2

species
1
2768161

 0.00
1

1

 0.00
29
356
0
order
13

335928
4
1

 0.00
1
0
family

1
genus
0
204476

 0.00
1
3

1
0
no rank
2688601
2
1

 0.00


 0.00
1
2789216
1
species

1

 0.00
4
2831111
1
0
family

1
0
genus
166953
1
3

 0.00

1
0
no rank
2649303
2
1

 0.00

species
1

 0.00
1
2795690

5
1
family
1
7

 0.00
119045

2282523
1
2

 0.00
2
genus
1

species
1
223967

 0.00
1

2
0
genus
407

 0.00
1
4

2
1

 0.00
114616
1
0
species

460265
1

 0.00
strain
1


 0.00
1
570505
1
species

family
0
4
82115

 0.00
1
9

34019

 0.00
1
5
3
0
genus

1
2

 0.00
556287
species
0
2

2
strain
658172
1

 0.00

0
species
1
34021

 0.00
1
2

strain
1

 0.00
1
1174529

0
no rank
1
227290

 0.00
1
3

357
2
1

 0.00
genus
0
1

species
1

 0.00
1
160699

4
1

 0.00
41294
0
family
2

374
1
3

 0.00
2
1
genus

0
no rank
1
1
2

 0.00
2631580

2782641
1

 0.00
species
1

13
0
order
204458
13
1

 0.00

13
0
family
1

 0.00
12
76892

20
3
1

 0.00
genus
0
1

1

 0.00
2
2640670
0
no rank
1

1
species
2903901
1

 0.00

genus
0
12

 0.00
1
8
41275

293

 0.00
1
1
species

1312364
1

 0.00
1
species

species
1
2774190
1

 0.00

no rank
0
8
2622653
1

 0.00
3

3068314
1

 0.00
species
1

2861285

 0.00
1
7
species

657642

 0.00
1
1
species

order
0
1
1921002
4
1

 0.00

family
0
1
1

 0.00
3
44746

0
genus
1
2900571
1

 0.00
2

species
1
1

 0.00
2806609

54526
1
5

 0.00
0
order
1

1655514
1
4

 0.00
1
family
0

0
genus
1
198251
1

 0.00
3

0
no rank
1
2
1

 0.00
2647897

species
1
1

 0.00
1002672

204457
1

 0.00
18
17
order
0

family
0
2
2820280

 0.00
1
4

2
0
genus
335405
3
1

 0.00

0
no rank
2
2
1

 0.00
2644549

species
2
1892855

 0.00
1

335929
1
3

 0.00
1
0
family

1
genus
0
2800685
2
1

 0.00

538382
1

 0.00
1
species

1
10

 0.00
41297
14
family
2

11
2
genus
7
1

 0.00
13687

1
species
13689
1

 0.00

185951
1

 0.00
5
species


 0.00
1
4
196159
0
no rank
3

1
species
2898433

 0.00
1

1

 0.00
3014784
1
species

1
species
2596913

 0.00
1

165695
2
1

 0.00
1
0
genus


 0.00
1
13690
1
species

580370
1

 0.00
4
1
class
0

0
order
1
580371

 0.00
1
3

2
1

 0.00
580372
1
family
0

1
genus
1

 0.00
377315

508458
1

 0.00
7
1
0
phylum

0
class
1
649775
6
1

 0.00


 0.00
1
5
649776
0
order
1

3029087
1

 0.00
4
family
0
1

81466
1
3

 0.00
1
genus
0

1

 0.00
2
81468
1
species
0

strain
1
572547
1

 0.00

0
no rank
5
2323

 0.00
1
16

1
15

 0.00
1783234
clade
0
5

1

 0.00
6
95901
0
clade
1

1
0
class

 0.00
1
5
2497643

order
0
1
1

 0.00
4
2497644

0
family
1
3
1

 0.00
2497645

1
0
genus
1551504
1
2

 0.00

673862
1

 0.00
species
1

0
phylum
2

 0.00
1
3
95818

2
no rank
2905966
2

1

 0.00
2
2905967
2
0
genus

2
species
2841264

 0.00
1


 0.00
1
5
221235
2
phylum
0

order
0
2
2900568
4
1

 0.00

3
1

 0.00
2900569
family
0
2

genus
0
2
2900570
1
2

 0.00

species
2
2735562

 0.00
1

1
19

 0.00
200940
4
0
phylum

1
0
class
1
6

 0.00
67799

1
order
0
188710
5
1

 0.00

3031464
4
1

 0.00
1
0
family

1
genus
0
241192

 0.00
1
3

species
0
1
171695
1
2

 0.00

strain
1
667014
1

 0.00

1
0
class
1
5

 0.00
3031651

4
1

 0.00
3031668
1
order
0

1

 0.00
3
213422
0
family
1

1

 0.00
2
115782
1
genus
0

1
species

 0.00
1
313985

2
class
0

 0.00
1
7
3024418

2
order
0
1
6

 0.00
213118

family
0
2
1
5

 0.00
213119


 0.00
1
2289
1
genus

28222

 0.00
1
3
genus
0
1


 0.00
1
2
28223
0
species
1

651182

 0.00
1
strain
1

6
1

 0.00
68525
2
0
clade

1
5

 0.00
28221
2
0
class

1
4

 0.00
1779134
2
order
0

2
family
0
3
1

 0.00
1779135

2766927
2
1

 0.00
genus
0
2


 0.00
1
2292766
2
species

32066
1

 0.00
15
0
phylum
12

12
class
0
203490
14
1

 0.00

203491
13
1

 0.00
order
0
12

family
0
1
203492
4
1

 0.00

848
1
3

 0.00
0
genus
1

1
2

 0.00
76857
0
species
1

1
strain
393480

 0.00
1

11
0
family
1

 0.00
8
1129771

32067
5
1

 0.00
9
0
genus

species
2
1

 0.00
109328

0
no rank
3
2633022

 0.00
1
2

species
3
1

 0.00
712362

species
4

 0.00
1
157691

2755140
2
1

 0.00
genus
0
2

157692
1

 0.00
species
2

0
clade
1
2498710
1

 0.00
8

1
0
phylum
67814

 0.00
1
7

0
class
1
693071
6
1

 0.00

order
0
1
5
1

 0.00
693072

1
4

 0.00
693073
0
family
1

693074

 0.00
1
3
1
0
genus

693075
1

 0.00
2
species
0
1

1

 0.00
511051
strain
1

8
phylum
0
29547
18
1

 0.00

class
0
1
1
6

 0.00
3031853

order
0
1
1
5

 0.00
213113

3048035
4
1

 0.00
1
0
family

1
genus
0

 0.00
1
3
84404

1
2

 0.00
84405
0
species
1

760142
1

 0.00
strain
1

3031852
1

 0.00
11
7
0
class

7
1
order
1
10

 0.00
213849

5
family
0
2808963
6
1

 0.00

1
genus
0
2321111
1
3

 0.00


 0.00
1
2
28200
species
0
1

strain
1
1032239

 0.00
1

4
0
genus
2321187
1

 0.00
2


 0.00
1
1904463
4
species

72294
3
1

 0.00
1
0
family

194
1
2

 0.00
1
genus
0

1
species
1848766
1

 0.00

clade
1
12
34
1

 0.00
1783257

3
1

 0.00
67812
1
phylum
0

1

 0.00
2
2789201
1
genus
0

1
species
1930593

 0.00
1

203682
1

 0.00
11
0
phylum
2

class
0
1

 0.00
1
5
203683

1
order
0
2691355

 0.00
1
4

1
family
0
1914233

 0.00
1
3

1
2

 0.00
2774146
0
genus
1

species
1
1

 0.00
2774151

1

 0.00
5
666505
1
0
class

1
0
order
1
4

 0.00
1771349

0
family
1
1771355
3
1

 0.00


 0.00
1
2
2807511
0
genus
1

species
1
2807512

 0.00
1

10
1

 0.00
74201
4
phylum
0

1
0
class
1

 0.00
6
414999

1
order
0
415001

 0.00
1
5

1
0
family
3056371

 0.00
1
4

442430
3
1

 0.00
genus
0
1

2
1

 0.00
395922
1
species
0


 0.00
1
583355
strain
1

3
0
class
203494
1

 0.00
3

1
2

 0.00
48461
0
order
3

3
family
1

 0.00
203557

phylum
0
4
204428
1

 0.00
9

204429
1

 0.00
8
0
class
4

3
1
order
1963360
1

 0.00
5

4
1

 0.00
92713
2
family
0

genus
0
2

 0.00
1
3
282132


 0.00
1
2
362787
species
0
2

2
strain
1

 0.00
264201

51291
1
2

 0.00
1
0
order

809
1

 0.00
1
family

0
phylum
3

 0.00
1
15
2818505

1
9

 0.00
3031711
2
subclass
0

1
0
order
3031713

 0.00
1
4

1
family
0
1
3

 0.00
224463

0
genus
1
1
2

 0.00
53

1
species

 0.00
1
2995304

0
order
1

 0.00
1
4
3031712

1524216
1
3

 0.00
0
family
1

1524217
1

 0.00
2
1
genus
0

1391654

 0.00
1
1
species

class
0
1
1

 0.00
5
32015


 0.00
1
4
29
order
0
1

1
0
suborder
80811
3
1

 0.00

1
0
family

 0.00
1
2
1524215

161492
1

 0.00
1
genus

1
phylum
0

 0.00
1
5
3002442

3002443
4
1

 0.00
1
class
0

3002444
1

 0.00
3
0
order
1

1

 0.00
2
227387
0
family
1

genus
1
1

 0.00
227388

1

 98.52
981
1783272
3149912
65
kingdom

3
0
phylum
1
11

 0.00
200795

class
0
1
32061
1
5

 0.00

3139175
4
1

 0.00
1
order
0

1
3

 0.00
3139177
0
family
1

1
genus
0

 0.00
1
2
3139173

3003348
1

 0.00
species
1

2364196
5
1

 0.00
class
0
2

1

 0.00
4
2364208
order
0
2

2
0
family
1

 0.00
3
2922432

0
genus
2
2922426
1
2

 0.00

2
species
2710758
1

 0.00

230
phylum
1
201174
1
149

 0.01

0
class
3
84995
1
5

 0.00

84996
1
4

 0.00
order
0
3

family
0
3
1

 0.00
3
84997

1
genus
3

 0.00
1
2
42255

49319
1

 0.00
species
2

84998

 0.00
1
6
1
0
class

1
0
order
84999
1

 0.00
5

1
0
family

 0.00
1
4
84107

1
0
genus
1

 0.00
3
33870


 0.00
1
2
33871
0
species
1

strain
1

 0.00
1
700015

class
3
225
1760
1
137

 0.01

order
0
10
1
11

 0.00
2037

10
1

 0.00
2049
family
1
10

2
genus
0
1654
1
4

 0.00

2609248
2
1

 0.00
1
no rank
0

species
1

 0.00
1
2708340

species
1
1

 0.00
1659

genus
0
5
1
2

 0.00
184869

species
5
1

 0.00
2811781

0
genus
2

 0.00
1
3
2529408

0
no rank
2
2691889
1

 0.00
2

1

 0.00
2758572
species
2

85006

 0.00
1
25
order
0
14

4
1

 0.00
85020
family
0
1

1
3

 0.00
43668
genus
0
1

2623841
1
2

 0.00
no rank
0
1

1
species

 0.00
1
3023517

1
0
family
85019
3
1

 0.00

genus
0
1

 0.00
1
2
1696

1
species
1

 0.00
273384

6
0
family
85023

 0.00
1
10

337004
1

 0.00
genus
1

46352
1

 0.00
3
1
0
genus

no rank
0
1
2615065
1
2

 0.00

species
1
2810513

 0.00
1

1705353

 0.00
1
genus
2

4
1

 0.00
33882
0
genus
2


 0.00
1
2
2609290
0
no rank
1

1
species
3049069

 0.00
1

69362

 0.00
1
species
1

0
family
6
7
1

 0.00
1268

0
genus
1
1

 0.00
2
57493

71999
1

 0.00
species
1

1269
1

 0.00
genus
2

32207
3
1

 0.00
genus
0
3

43675

 0.00
1
2
3
species
2

strain
1
1

 0.00
680646

1
0
order
85008

 0.00
1
5

28056
1

 0.00
4
1
0
family

0
genus
1
265431
1

 0.00
3

2627926

 0.00
1
2
0
no rank
1


 0.00
1
3016090
1
species

85007
1
29

 0.00
20
0
order

0
family
11
1653
1
7

 0.00

1716

 0.00
1
6
genus
2
11

1
species
1717
1

 0.00


 0.00
1
1718
species
1

441500

 0.00
1
species
2

2
species
65058

 0.00
1

3
species
38304

 0.00
1

family
0
1
85029
1

 0.00
3

37914
2
1

 0.00
genus
0
1

species
1
1

 0.00
322509

2
0
family

 0.00
1
5
85025

1827
1

 0.00
2
0
genus
1

103816
1

 0.00
1
species

1

 0.00
2
1817
1
0
genus


 0.00
1
37329
species
1

1762
1
13

 0.00
6
1
family

1866885
3
1

 0.00
genus
0
1

0
species
1
1
2

 0.00
152142

strain
1
1226752

 0.00
1

670516

 0.00
1
2
1
0
genus


 0.00
1
1774
1
species

3
genus
0
1

 0.00
7
1763


 0.00
1
3
77643
2
species group
0

1
species
2
1773
1

 0.00
2

1

 0.00
1010835
1
strain

0
species group
1
120793

 0.00
1
3

1
2

 0.00
339268
0
species
1

1
strain
1

 0.00
1041522

order
0
1
1
5

 0.00
85010

1
family
0
2070
4
1

 0.00

1
0
genus
1
3

 0.00
1847

0
species
1
240495
1

 0.00
2

strain
1
1

 0.00
675635

0
order
1
4
1

 0.00
1643682

85030
1

 0.00
3
1
family
0

1
0
genus
88138

 0.00
1
2


 0.00
1
477641
species
1

12
order
0
1

 0.00
11
85004

1

 0.00
10
31953
12
0
family

10
0
genus
1678
7
1

 0.00

1

 0.00
2
216816
5
species
0


 0.00
1
1682
subspecies
5

2
species
1680

 0.00
1

0
species
3

 0.00
1
3
28025

3
subspecies
0
1

 0.00
2
302911

strain
3
1

 0.00
1316911

2
0
genus
1

 0.00
2
2701

1

 0.00
2702
species
2

1
0
order
1

 0.00
4
85012

1
0
family
1

 0.00
3
2004

genus
0
1
147067
1

 0.00
2

species
1
1

 0.00
2006

29
1

 0.00
85009
order
0
156

family
0
141
31957
7
1

 0.00

1912216
1
4

 0.00
140
1
genus

33011
1

 0.00
1
species

species
1
33010

 0.00
1


 0.00
1
1747
species
137

1
0
genus
1743
1
2

 0.00

species
1
1744
1

 0.00

1
3

 0.00
2726069
1
family
0

1

 0.00
2
182639
1
0
genus

1
no rank
2644121

 0.00
1

14
0
family
18
1

 0.00
85015

1
0
genus
1

 0.00
3
2040

1
0
no rank
1

 0.00
2
2633570

species
1
1

 0.00
2107713

3
1

 0.00
1546255
genus
0
1

2621872
2
1

 0.00
1
no rank
0

1
species
2708082
1

 0.00

1
2

 0.00
53387
0
genus
2

1

 0.00
546871
2
species

10
1
genus
1839
1
9

 0.00

species
1
2894081
1

 0.00

no rank
0
2
2615069
1
3

 0.00

species
1

 0.00
1
3044187


 0.00
1
2714939
species
1

species
1
2558918

 0.00
1

160826

 0.00
1
3
species


 0.00
1
2803858
1
species

2849501
1

 0.00
1
species

0
order
5
85011

 0.00
1
8

family
0
5
2062
1

 0.00
7

1
6

 0.00
1883
genus
0
5

0
no rank
4
2593676
1
4

 0.00

1882757

 0.00
1
1
species

2594462

 0.00
1
species
2

1
species
2866715

 0.00
1

1
species
116188

 0.00
1

1
0
order
5
1

 0.00
85014

85034

 0.00
1
4
0
family
1

0
genus
1
3
1

 0.00
283810

283811
1

 0.00
2
species
0
1

strain
1
446470
1

 0.00

clade
0
14
1798711
1

 0.00
40

1117
1

 0.00
39
14
phylum
0

1

 0.00
38
3028117
class
1
14

2
order
0

 0.00
1
5
1890505

1890528
4
1

 0.00
0
family
2

54298
1
3

 0.00
2
genus
0

0
no rank
2
2646205
1
2

 0.00

species
2
155894
1

 0.00

1890424

 0.00
1
7
order
0
2

1890426
6
1

 0.00
family
0
2

1
0
genus
2
1

 0.00
1129

no rank
1

 0.00
1
2626047

1
0
genus
13034
1

 0.00
3


 0.00
1
2
292566
0
species
1

1
strain
13035
1

 0.00

1

 0.00
8
3079749
order
0
3

1890438
1

 0.00
7
3
family
0

1
6

 0.00
3081713
0
no rank
3

47251
5
1

 0.00
3
genus
0

2650499
1

 0.00
2
0
no rank
1

species
1
1

 0.00
2812896


 0.00
1
2
1184
0
species
2

2
strain
3060204

 0.00
1

1161
1

 0.00
9
2
order
0

2661849
1
4

 0.00
1
0
family

0
genus
1
1
3

 0.00
1186

2619626

 0.00
1
2
0
no rank
1

2005462

 0.00
1
species
1

0
family
1

 0.00
1
4
1182

1
3

 0.00
383614
1
genus
0

1397703
1
2

 0.00
species
0
1

415709

 0.00
1
strain
1

subclass
0
4
1301283
8
1

 0.00

1150
1

 0.00
7
4
order
0

0
no rank
1

 0.00
1892256

1892252

 0.00
1
2
family
0
2

2
genus
54304

 0.00
1

3
1892254
family
2

2023214
1

 0.00
3
genus
0
2

no rank
0
2
2
1

 0.00
2781947

2
species
222819
1

 0.00

1154676
1
6

 0.00
phylum
0
2

2
0
class
1
5

 0.00
2829293

4
1

 0.00
2599632
order
0
2

family
0
2
2829291

 0.00
1
3

2
1

 0.00
3016049
2
0
genus

3016050
1

 0.00
species
2

3149558
616
phylum
719
1

 98.51
1239

11
class
0
526524
12
1

 0.00


 0.00
1
11
526525
order
0
11

2810281

 0.00
1
2
2
0
family

191303
1

 0.00
genus
2


 0.00
1
8
128827
9
family
2

1
5

 0.00
1647
0
genus
5

2
no rank
2624170
1

 0.00

2485784

 0.00
1
species
2

1
0
species
1648
1

 0.00
2

1

 0.00
3119429
1
subspecies


 0.00
1
2
2679910
2
genus
0

2
species

 0.00
1
2487118

class
0
24
909932

 0.00
1
12

0
order
23
909929
1

 0.00
8

1843490
1
4

 0.00
1
0
family

0
genus
1
81463

 0.00
1
3

1
no rank
0
673272
2
1

 0.00

673273
1

 0.00
species
1

1843491
1

 0.00
3
22
family
0

22
0
genus
158846
1

 0.00
2

1

 0.00
437897
species
22

3
1

 0.00
1843489
order
0
1

1
2

 0.00
31977
1
family
0

genus
1

 0.00
1
29465

class
0
7
1737404
12
1

 0.00

order
0
7
11
1

 0.00
1737405

family
0
3
1
4

 0.00
2042895

0
genus
3
3
1

 0.00
1505664

3
0
species
1556
1
2

 0.00

1

 0.00
1128398
3
strain

1570339
1
3

 0.00
0
family
1


 0.00
1
162289
0
genus

genus
1
1
2981628


 0.00
1
54006
species
1

3
family
0
3
1

 0.00
2848916

3
genus
0
171003
1
2

 0.00

116090

 0.00
1
species
3

class
7923
3148778
91061
1

 98.49
539

988
24
order
196
1

 0.03
1385

67
family
3
186822

 0.00
1
22

genus
4
63
44249
1
20

 0.00

1
species

 0.00
1
2509456

2044880

 0.00
1
2
2
0
species group

species
2
373687

 0.00
1


 0.00
1
1178515
1
species

1
species
1619311
1

 0.00

species
2
61624

 0.00
1


 0.00
1
10
185978
16
no rank
38

species
3
3047872
1

 0.00

species
2
2921656
1

 0.00

4
species
1

 0.00
2921641

2954726
1

 0.00
1
species


 0.00
1
2954732
species
2

species
1
2954506
1

 0.00

4
species
2777983
1

 0.00

3
species
1

 0.00
1536770

species
2

 0.00
1
2921450


 0.00
1
1338368
species
1

7
species
13

 0.00
1
2
1406

strain
6
1413214

 0.00
1

genus
1
1

 0.00
55080

family
0
540
186820
1

 0.02
11

538
314
genus
1637
1
8

 0.02

210
209
species
1639
1
2

 0.01

882095

 0.00
1
strain
1

1640
1

 0.00
species
1

2
species
4
1
2

 0.00
1642

strain
2
272626

 0.00
1

species
3
1641
1

 0.00


 0.00
1
1643
species
6

genus
0
2
2755

 0.00
1
2

1

 0.00
2756
species
2

1
5

 0.00
186824
1
family
3

0
genus
1
1274351
2
1

 0.00

species
1
1380685

 0.00
1

genus
0
1
292635

 0.00
1
2


 0.00
1
37482
species
1


 0.00
1
17
186818
family
0
8


 0.00
1
3
1569
0
genus
1

0
no rank
1

 0.00
1
2
2647733

1

 0.00
1930546
species
1

genus
0
3

 0.00
1
3
648800

76853

 0.00
1
2
3
0
species

1002809

 0.00
1
strain
3

1

 0.00
2
651660
genus
0
1

2320858
1

 0.00
species
1

1
0
genus
3
1

 0.00
1372

0
no rank
1
2662419
2
1

 0.00

species
1
1

 0.00
2908840

1
0
genus
1

 0.00
3
889388


 0.00
1
2
2625558
1
no rank
0


 0.00
1
2921704
species
1

0
genus
1
1

 0.00
2
648802

1

 0.00
241244
species
1

186817
103
1

 0.01
220
17
family

1
genus
289201

 0.00
1

3
0
genus
2675229
1

 0.00
3

species
1
1

 0.00
450367

species
2

 0.00
1
228899

0
genus
1
1055323
1

 0.00
3

no rank
0
1
2640495
1
2

 0.00


 0.00
1
2954706
species
1


 0.00
1
3
2675234
1
0
genus

2675276
1
2

 0.00
1
no rank
0

2926399

 0.00
1
species
1

5
0
genus
1
2

 0.00
150247


 0.00
1
294699
species
5

0
genus
5
1
4

 0.00
84406

1
0
no rank
1
2

 0.00
2620237

1
species

 0.00
1
2700081


 0.00
1
1482
species
4

genus
1
16

 0.00
1
4
2837504

1398
2
1

 0.00
13
species
5

8
strain
345219
1

 0.00

38875
1

 0.00
2
species

genus
0
3
2675230

 0.00
1
3

1

 0.00
665099
species
1

1

 0.00
1399
species
2

1329200

 0.00
1
2
genus
0
1

1
species
1

 0.00
1221500

1
2

 0.00
2837506
1
0
genus


 0.00
1
1737571
species
1

1
0
genus
1

 0.00
3
175304

2614679

 0.00
1
2
no rank
0
1


 0.00
1
2518176
species
1

genus
0
5
1

 0.00
6
129337

3
species
860353

 0.00
1

1505648

 0.00
1
3
1
0
species group

species
0
1
1462
1

 0.00
2


 0.00
1
235909
1
strain

1
species
1

 0.00
129338

2944627

 0.00
1
2
genus
0
1

2592382
1

 0.00
species
1

4
genus
0
400634

 0.00
1
4

1
2

 0.00
2636778
no rank
0
3

1

 0.00
2936682
3
species

species
1
2086577
1

 0.00

1
genus
0
2675233
1

 0.00
3

2675274
2
1

 0.00
1
no rank
0

1

 0.00
2897333
1
species

1

 0.00
36
1386
genus
9
137

86661
1

 0.00
14
0
species group
80

62
5
species
8
1

 0.00
1396

1

 0.00
226900
strain
2

0
no rank
1
1
2

 0.00
1179100

637380
1

 0.00
1
strain


 0.00
1
1454382
strain
2


 0.00
1
572264
strain
1

526991
1

 0.00
strain
50

1

 0.00
288681
1
strain

1392
3
1

 0.00
7
species
5

1
strain
1392837
1

 0.00

1

 0.00
260799
1
strain

11
7
species
1

 0.00
2
1428

1

 0.00
29339
no rank
4

1

 0.00
2672567
2
species

species group
0
2

 0.00
1
2
1792192

293387
1

 0.00
species
2

1

 0.00
86663
species
2

1
12

 0.00
653685
1
species group
35

8
5
species
1423

 0.00
1
4

2
subspecies
1
135461
1
2

 0.00

1302650

 0.00
1
strain
1

subspecies
1

 0.00
1
86029

species subgroup
3
24
1938374

 0.00
1
4

6
species
1390
1

 0.00

13
species

 0.00
1
492670

species
2

 0.00
1
659243

1
species

 0.00
1
483913

0
species subgroup
1
653388
1

 0.00
2

1

 0.00
260554
species
1


 0.00
1
5
185979
7
0
no rank

2884834

 0.00
1
1
species

species
3

 0.00
1
1071078

species
1
1

 0.00
98228

1

 0.00
467262
2
species

4
genus
0
1

 0.00
6
2675232

2675272
1

 0.00
5
0
no rank
4

1
species
3070676
1

 0.00

2954672
1

 0.00
species
1

1
species

 0.00
1
3070682

3070677

 0.00
1
1
species

genus
0
1
1

 0.00
2
2800373

1404
1

 0.00
species
1

2837508
1

 0.00
2
0
genus
2

189381
1

 0.00
species
2

genus
2
2685905

 0.00
1

45667
2
1

 0.00
0
genus
3

45668

 0.00
1
species
3

0
genus
1
3
1

 0.00
1906945

1
0
species
1426

 0.00
1
2


 0.00
1
634956
strain
1

484508
1
3

 0.00
genus
0
2

2620869
1

 0.00
2
no rank
0
2

2
species

 0.00
1
2921616

0
genus
2
351195
1
2

 0.00

1230341
1

 0.00
2
species

family
0
3
186823
1

 0.00
3

29330

 0.00
1
1
genus

432330
1

 0.00
2
genus

0
family
6

 0.00
1
3
539738

1378
2
1

 0.00
6
genus
5

1

 0.00
1379
1
species

539002
1

 0.00
6
0
no rank
4

1
5

 0.00
539742
4
no rank
0

33986

 0.00
1
4
4
genus
0

species
0
3
132920
1

 0.00
2

strain
3
1087448
1

 0.00

1

 0.00
33987
1
species

113
1
family
90964
1
25

 0.00

20
1

 0.00
1279
17
genus
105

1

 0.00
33028
species
1

1

 0.00
2912228
1
species

1

 0.00
246432
species
1

214473

 0.00
1
3
species

species
1
3051183
1

 0.00

species
2
1

 0.00
28035

29380

 0.00
1
species
1


 0.00
1
70255
species
1

5
0
species group

 0.00
1
2
2815305

species
5
283734
1

 0.00

species
49
51
1280
3
1

 0.00

1
strain
1229492
1

 0.00

strain
1
1

 0.00
1392476

species
1

 0.00
1
1290

1
species
522262
1

 0.00

1

 0.00
1282
species
17

1286
2
1

 0.00
1
species
0


 0.00
1
1287
no rank
1

29378

 0.00
1
1
species

69965
1

 0.00
genus
5

3
1

 0.00
2803850
2
0
genus

1

 0.00
42858
1
species

1
species
1

 0.00
1296


 98.21
1
342
186826
3139867
371249
order

1

 85.68
124
1300
2739337
family
143909

1301
1
84

 0.82
26076
genus
678

1
species
2173853

 0.00
1

1302

 0.00
1
18
species

2
species
1

 0.00
1341

14
species
1329

 0.00
1

1318
1

 0.00
2
1
species
3


 0.00
1
760570
2
strain

no rank
1
61

 0.00
1
11
2608887

1
species
2610896
1

 0.00

1

 0.00
2972784
species
2

species
1
3028084

 0.00
1

2420310
1

 0.00
1
species

1902136
1

 0.00
1
species

1

 0.00
2759692
3
species

1
species

 0.00
1
3077584

species
4

 0.00
1
1839799

2954513

 0.00
1
5
species

2598457

 0.00
1
species
41

5
species
82348

 0.00
1

138
species
1

 0.00
59310


 0.00
1
361101
18
species

1

 0.00
1814128
1
species

10
species
11
1
3

 0.00
1314

0
serotype
1
798299
1

 0.00
2

strain
1
1

 0.00
798300

1
species
197614
1

 0.00

1
3

 0.01
1304
species
146
164

4
strain
1048332
1

 0.00

14
strain

 0.00
1
1200793

16814
species
16968
1308
1

 0.53
12

strain
10
1435981

 0.00
1

31
strain
1

 0.00
1408178


 0.00
1
1435972
21
strain

1

 0.00
1433288
strain
17

4
strain
1051074
1

 0.00

1
strain
264199

 0.00
1

1

 0.00
1187956
strain
1


 0.00
1
1433289
strain
33

1
strain
767463
1

 0.00

strain
22

 0.00
1
1435974

13
strain

 0.00
1
1436725

1310
1

 0.00
49
species

1303
1

 0.00
4
species
41
88

strain
1
927666

 0.00
1

20
subspecies
1458253

 0.00
1

26
subspecies
1077464
1

 0.00


 0.00
1
28037
species
4

species
115
119
3
1

 0.00
1311

strain
1
1203670
1

 0.00

3
strain
1309807
1

 0.00

species group
0
4319
1
5

 0.14
119603

1334
1
3

 0.14
454
species
4317

1

 0.12
99822
subspecies
3857

subspecies
6
1

 0.00
119602

species
2
1336
1

 0.00

1
2

 0.00
1305
38
species
36

2
strain
388919
1

 0.00

species
18

 0.00
1
1343

1

 0.00
68892
12
species

1346
1

 0.00
47
species

species
3
1326

 0.00
1

1309
1
2

 0.00
4
3
species

1
strain
1441364
1

 0.00

102684

 0.00
1
2
species

1

 0.00
2993430
1
species

670
species
1501662

 0.02
1

1
2

 0.02
1348
594
species
623

strain
29
1

 0.00
873447

species
25
27
1313
1
2

 0.00

2
strain

 0.00
1
869311

1

 0.00
149016
species
9

150055
1

 0.02
species
790

1307

 0.03
1
species
1039

29389
1

 0.00
species
62

13
species group
0
671232
1

 0.00
5

species
0
2
76860
1

 0.00
2

subspecies
2
1

 0.00
184250

1

 0.00
1328
6
species

1338
1

 0.00
5
species

2
0
species
45634
2
1

 0.00

2
strain
889201

 0.00
1

45
species

 0.00
1
315405

684066
1

 0.00
species
7

2
species
1811193
1

 0.00

1357

 80.36
1
37
2569349
genus
848326

species
37
1

 0.00
2419773

319
species
2305
1

 0.07
2
1281486

1

 0.06
2906461
1986
subspecies

2749961

 0.00
1
18
species

1

 0.01
2
1364
species
0
257

297352

 0.01
1
strain
257


 0.02
1
1151742
676
species

6901
species
6872
1363
1
3

 0.22

420889
1

 0.00
strain
1


 0.00
1
1890280
28
subspecies

no rank
94
2859
2643510
1
5

 0.09

3037457
1

 0.04
1325
species

species
20
1

 0.00
2816910

species
654

 0.02
1
2879149


 0.02
1
2816912
species
766

1

 13.08
8
1358
species
302766
418225

115459
subspecies
56112
1360
1

 3.61
7

12
strain
1

 0.00
929102


 0.02
1
1046624
strain
618

58
strain
1117941

 0.00
1


 1.83
1
44688
58592
no rank

strain
1
272623
1

 0.00

66
strain
684738

 0.00
1

species
1269
1940789

 0.04
1

31
species

 0.00
1
2749962

2592653

 0.00
1
species
15

1

 0.57
1366
species
18068

species
1232477
1270362
1

 39.73
9
1359

37885
subspecies
63
1
8

 1.18
2816960

strain
7592
1295826

 0.24
1

1540
strain
1449093
1

 0.05

1104322
1

 0.22
6949
strain

strain
13155

 0.41
1
1111678

8469
strain
272622

 0.26
1

19
strain
746361
1

 0.00


 0.00
1
416870
98
strain

3
genus
0
1
2

 0.00
1930830


 0.00
1
1859475
3
species

15445
216
family
1
143

 0.48
33958

1

 0.00
2
3118928
1
genus
2

2932184

 0.00
1
species
1

genus
2
985
2742598
6
1

 0.03

species
1
1633

 0.00
1

1
2

 0.00
1598
4
species
7

1358027

 0.00
1
3
strain

1
2

 0.03
1613
species
677
975

strain
298

 0.01
1
1381124

genus
34
3909
1
27

 0.12
1578

1584
1

 0.00
4
40
species
30

1
subspecies
3
1
2

 0.00
1585

390333
1

 0.00
2
strain

1

 0.00
249265
7
subspecies

1

 0.00
3
2620435
no rank
0
2

2879107

 0.00
1
1
species

species
1
2690380

 0.00
1

species
3
2012495
1

 0.00

1
species
1

 0.00
1596

1

 0.00
227945
species
1

1579
1
2

 0.00
species
0
1

1

 0.00
272621
1
strain


 0.00
1
1604
species
1

1
species
1

 0.00
33959

1218493
1

 0.00
species
2

1

 0.01
508451
336
species

species
1
109790
1

 0.00

1587

 0.11
1
5
1615
species
3439

3
strain
767456
1

 0.00

subspecies
37
44272
1

 0.00

585520
1

 0.00
strain
38


 0.05
1
405566
1746
strain

83683
1

 0.00
21
species

6
species
47770
1

 0.00

12
species
20

 0.00
1
2
267818

subspecies
8
190906
1

 0.00

genus
0
2
2930384
1
2

 0.00

species
2
1

 0.00
2506420

3
0
genus
2767881

 0.00
1
4

species
1
53444

 0.00
1


 0.00
1
2
1625
2
species
1

1
strain
714313
1

 0.00

1253
7
1

 0.00
genus
5
53

1

 0.00
1255
species
10

species group
0
33
1
2

 0.00
1670797


 0.00
1
1254
species
33

1
2

 0.00
554805
2
0
no rank

2
species

 0.00
1
2962679

3
species

 0.00
1
51663

3
0
genus
2767888
2
1

 0.00


 0.00
1
468911
3
species

1
genus
0
2
1

 0.00
2767891

304207

 0.00
1
species
1

12
genus
221
2759736
11
1

 0.01

57037

 0.00
1
1
species

3025672

 0.00
1
species
2

1
species
171523

 0.00
1

190
species
170
1597
4
1

 0.01

subspecies
11
1

 0.00
47714


 0.00
1
1446494
1
strain

113557

 0.00
1
8
subspecies

2
species

 0.00
1
2100821

47715
2
1

 0.00
13
11
species

1088720
1

 0.00
strain
2

69
0
genus
1
4

 0.00
2767886

267363
1

 0.00
species
2

1580

 0.00
1
2
67
species
66

strain
1

 0.00
1
387344

2767887
1
6

 0.01
196
0
genus

154
species
89059

 0.00
1

1
species
762833
1

 0.00

19
species

 0.00
1
1624

7
species
1622

 0.00
1

species
15
1623

 0.00
1

1
genus
0
2
1

 0.00
2978367

species
1
2925830

 0.00
1

25
1
genus
7
1

 0.00
2767879


 0.00
1
2304606
1
species


 0.00
1
2
1612
species
1
2

strain
1
936140
1

 0.00

19
0
species

 0.00
1
2
1602

19
strain
1

 0.00
1423720


 0.00
1
1847728
species
2

8
0
genus
7
1

 0.00
2767893

152331
1

 0.00
1
species

1
species
0
1
3

 0.00
1581

2737512
2
1

 0.00
subspecies
0
1

1
strain

 0.00
1
1071400

species
1

 0.00
1
2883998

5
species

 0.00
1
33962

1
2

 0.00
2767882
130
0
genus


 0.00
1
231049
species
130

1243
1
13

 0.03
800
11
genus

species
1
1252

 0.00
1

85
species
1511761
1

 0.00

33964

 0.00
1
species
7

33968

 0.00
1
species
2

0
no rank
1
2685106
1
2

 0.00


 0.00
1
2571156
1
species


 0.00
1
2
3016637
3
1
species group

115778

 0.00
1
species
2

1246
1

 0.00
species
79

species
474
611
1245
3
1

 0.02

subspecies
12
33966

 0.00
1

125
subspecies
33967

 0.00
1

20
0
genus
1
3

 0.00
2767876

1603

 0.00
1
2
0
species
20

1

 0.00
1423721
20
strain

7
1

 0.21
2767842
6578
17
genus

species
2
1589

 0.00
1

6559
6544
species

 0.21
1
5
1590


 0.00
1
1304889
strain
4


 0.00
1
337330
subspecies
3

7
strain
1

 0.00
220668

1
strain

 0.00
1
1385856

genus
6
2150
2767885

 0.07
1
5

16
species
17
1599
1
3

 0.00

214326
1
2

 0.00
0
subspecies
1

1423833

 0.00
1
strain
1

28038

 0.07
1
species
2127

2767877
1
3

 0.00
5
1
genus

1

 0.00
2419772
species
1

1

 0.00
148814
3
species

1

 0.00
4
46254
2
genus
0

1
species
1

 0.00
2203724

1247
1

 0.00
2
1
species
0

1
strain
203123
1

 0.00

genus
0
5
1
6

 0.00
2767889

species
2
375175

 0.00
1

species
1
1607

 0.00
1

2
1
species
3
1

 0.00
1610

2
1

 0.00
115542
0
subspecies
1

1423822
1

 0.00
1
strain

2767874
2
1

 0.00
5
0
genus

5
species
1

 0.00
1720083

genus
2
50
46255
6
1

 0.00

species
34
1

 0.00
1249

species
1
1

 0.00
2714949

1

 0.00
155866
species
4

137591
1

 0.00
3
species

1631871

 0.00
1
6
species

0
genus
6
2767892

 0.00
1
2

1

 0.00
240427
6
species

36
1
family

 0.00
1
15
186827

1

 0.00
4
1375
18
genus
0

128944

 0.00
1
species
2

1

 0.00
1377
2
species

species
14
51665
1

 0.00

2
1

 0.00
2761542
0
genus
2

1

 0.00
2969821
2
species

2862144
1
2

 0.00
0
genus
10

10
species
87458

 0.00
1

46123
3
1

 0.00
0
genus
2

2
1
species
1
2

 0.00
46125

1
strain
592010

 0.00
1

genus
1

 0.00
1
13075

genus
0
2
89092
2
1

 0.00

89093
1

 0.00
2
species

81852
1
41

 0.42
13556
1444
family

1

 0.36
29
1350
genus
536
11377

species
4100
1

 0.13
37734

1

 0.00
53345
species
31

33945
1

 0.00
species
1

1
species
2839759

 0.00
1

1

 0.00
1353
species
7

species
9
1805431
1

 0.00


 0.00
1
8
2608891
34
no rank
0

9
species
2060307

 0.00
1

species
1
1834193
1

 0.00


 0.00
1
2420313
2
species

2920934
1

 0.00
species
16

species
4

 0.00
1
2603296

1

 0.00
3044568
species
1

1
species
2230879
1

 0.00

160453

 0.01
1
species
182

534
species
540

 0.02
1
5
1351


 0.00
1
565650
1
strain

strain
2

 0.00
1
565638

2
strain
1201292
1

 0.00

936153
1

 0.00
strain
1

5570
5568
species
1

 0.17
2
1352

565664
1

 0.00
2
strain

44008

 0.00
1
species
6

28
species
1354

 0.00
1

246144

 0.00
1
species
1

species
6
1

 0.00
71452

species
2
53346
1

 0.00

species
323
1

 0.01
357441

2737
1
7

 0.02
2
genus
694

species
3
1

 0.00
2562451

species
8

 0.00
1
2738

218144

 0.00
1
species
2

2991418

 0.01
1
345
species

0
no rank
334
2648499
2
1

 0.01

2925838

 0.01
1
334
species

genus
2
41
51668
4
1

 0.00

1
species
526944
1

 0.00

51669
2
1

 0.00
31
species
38

1

 0.00
945021
strain
7

family
0
244
186828

 0.01
1
18

117563
2
1

 0.00
0
genus
2

137732

 0.00
1
species
2

1
5

 0.00
1470540
2
genus
7

2
0
no rank

 0.00
1
2
2621505

2908211
1

 0.00
2
species

2
species

 0.00
1
1868794

species
1
708126
1

 0.00

228
0
genus

 0.01
1
5
2747

2
1

 0.00
257487
no rank
0
2

2
species
208596
1

 0.00

1

 0.01
2751
223
species

3
species
2748

 0.00
1

0
genus
5
191769
3
1

 0.00

1

 0.00
2
2632303
5
0
no rank

5
species
1

 0.00
1911586

29393
2
1

 0.00
2
genus
0

29394

 0.00
1
species
2

1

 0.00
143
186801
9
class
122

9
1

 0.00
2770089
2
0
order

1
0
family
4
1

 0.00
2770092

0
genus
1
499228

 0.00
1
3

499229

 0.00
1
2
0
species
1

1

 0.00
1209989
1
strain

2770093
1

 0.00
4
family
0
1

genus
0
1
291988
1

 0.00
3

1
species
0

 0.00
1
2
291990

strain
1
1

 0.00
555079

20
0
order
1

 0.00
19
3082720

1

 0.00
3
3030910
family
0
1

2060094

 0.00
1
2
0
genus
1

1
species
2697030
1

 0.00

19
1
family
186804
15
1

 0.00

1505657
1
2

 0.00
0
genus
5


 0.00
1
261299
5
species

2
1

 0.00
1505652
genus
0
1


 0.00
1
1460447
species
1

1849822
1

 0.00
2
1
genus
0

1505

 0.00
1
1
species

1501226
4
1

 0.00
4
genus
0

no rank
0
4
2626894
1
3

 0.00

1

 0.00
2708053
species
3

1
species
2724150

 0.00
1

genus
0
7
1870884
1
4

 0.00

5
species
7
1
3

 0.00
1496

strain
1

 0.00
1
699037

699035
1

 0.00
strain
1


 0.00
1
9
53433
2
0
order

0
family
1
1
4

 0.00
3046412

3
1

 0.00
32636
0
genus
1


 0.00
1
2
31909
1
0
species

strain
1
373903

 0.00
1

1
0
family
4
1

 0.00
53434

1
3

 0.00
42417
genus
0
1

species
0
1
42422
2
1

 0.00

1
strain

 0.00
1
748449

order
0
18
24
1

 0.00
3085636

3018741
4
1

 0.00
1
0
family

1

 0.00
3
698776
1
0
genus

0
species
1
1

 0.00
2
29360

642492
1

 0.00
strain
1

186803
1
16

 0.00
3
family
16

2997360
1

 0.00
2
3
genus
0

1

 0.00
2934315
species
3

1

 0.00
2
2383
genus
0
3

species
3
1

 0.00
420336

0
genus
1
1

 0.00
3
248744

1
species
0
1

 0.00
2
168384

strain
1
478749
1

 0.00

1
2

 0.00
1506577
0
genus
1

36835

 0.00
1
1
species

1

 0.00
2
2944193
0
genus
1

1
species
2763672

 0.00
1

572511

 0.00
1
1
genus


 0.00
1
3
2316020
0
genus
3

0
species
3
33038
1
2

 0.00

3
strain
1

 0.00
411470

family
0
1
1
3

 0.00
2603322

2603323
1

 0.00
2
1
0
genus

species
1

 0.00
1
2173034

3039167
1
6

 0.00
order
0
3

3
0
family
3039168

 0.00
1
5

1

 0.00
44260
2
genus

genus
0
1
202949
3
1

 0.00

161154

 0.00
1
2
species
0
1

1
strain

 0.00
1
698762

57
4
order
60
1

 0.00
186802

1
family
1

 0.00
68298

2
family
0
1491775

 0.00
1
3

2838170

 0.00
1
2
0
genus
2

species
2
1

 0.00
2731377

186807
1
4

 0.00
1
family
0

3
1

 0.00
2916693
0
genus
1

59610
1

 0.00
2
1
0
species


 0.00
1
349161
strain
1


 0.00
1
29
31979
30
0
family

4
1

 0.00
44258
2
0
genus

2629145
3
1

 0.00
0
no rank
2

3029174

 0.00
1
1
species

1

 0.00
3023090
1
species

1

 0.00
3
1155385
genus
0
1

no rank
0
1
2630696
1
2

 0.00

1
species

 0.00
1
3036945

0
genus
1

 0.00
1
3
1266

no rank
0
1
1
2

 0.00
2619470

1
species
2726954
1

 0.00

1485
1
18

 0.00
26
genus
1

1491
1

 0.00
1
species

1
species
29341

 0.00
1

1

 0.00
1492
species
1

1509

 0.00
1
species
3

36839
2
1

 0.00
1
species
0

1
strain
1121310
1

 0.00

1

 0.00
2320868
species
1

species
1

 0.00
1
1548

species
4
1

 0.00
1502

3
species
1520

 0.00
1

3
species
1488

 0.00
1

2614128
4
1

 0.00
4
no rank
0

2930219

 0.00
1
1
species


 0.00
1
1195166
species
1

1

 0.00
1155388
species
2

2
species
0
217159

 0.00
1
2

2
strain
536227

 0.00
1

1
0
family

 0.00
1
4
2937909

56112
1

 0.00
3
0
genus
1

1
0
no rank
2635733

 0.00
1
2

2907827
1

 0.00
1
species

3
family
16
1

 0.00
14
216572


 0.00
1
2
216851
1
genus
2

853

 0.00
1
species
1

2304691
1

 0.00
2
4
0
genus

species
4

 0.00
1
1510

1

 0.00
3
1637257
1
0
genus

2
1

 0.00
884684
species
0
1

699246
1

 0.00
strain
1


 0.00
1
4
35829
0
genus
4

species
1
1515
1

 0.00

288965
1

 0.00
2
3
0
species


 0.00
1
720554
strain
3


 0.00
1
2
244127
2
genus
0

species
2

 0.00
1
169435

family
0
1
186806
2
1

 0.00

1
genus
33951

 0.00
1

3046368
1

 0.00
2
0
family
1

1

 0.00
3046369
genus
1

0
order
10
1
10

 0.00
68295

1
family
0

 0.00
1
4
543372

252965

 0.00
1
3
0
genus
1

1
0
species
252966
2
1

 0.00

1
strain
697281
1

 0.00

1

 0.00
5
186814
family
0
9

genus
0
7
28895
1

 0.00
3

7
species
0
29329

 0.00
1
2

858215
1

 0.00
7
strain

2
genus
1754
1

 0.00


 0.00
1
5
485256
1
order
0

485255
4
1

 0.00
1
0
family

375928
1

 0.00
3
1
0
genus

1

 0.00
2
375929
1
0
species

1

 0.00
457570
strain
1

544448

 0.00
1
49
phylum
0
38

0
order
15
1
14

 0.00
2790996

1
3

 0.00
2790998
4
1
family

3
genus
2
2995234
1
2

 0.00

1
species

 0.00
1
2096

0
family
11
2895623
1

 0.00
10

2767358
1
5

 0.00
genus
3
7

species
1
1

 0.00
55603

species
1
171282

 0.00
1

1
species
1

 0.00
33923

1

 0.00
28903
species
1


 0.00
1
2
2923352
2
genus
1

1
species
2099

 0.00
1

2
1

 0.00
2895509
2
genus
0

2111
1

 0.00
2
species

31969
29
1

 0.00
22
4
class

order
0
10

 0.00
1
15
2085

7
family
0
2092
10
1

 0.00

2093
1
9

 0.00
1
genus
7

1
species group
2
656088
3
1

 0.00

0
species
1
1

 0.00
2
2095


 0.00
1
40480
1
subspecies

no rank
0
4
2683645
5
1

 0.00

1749074
1

 0.00
species
1

1
species
640330

 0.00
1


 0.00
1
2810348
species
1

2683967

 0.00
1
species
1

1

 0.00
4
3132029
3
0
family

3
0
genus
295595
1
3

 0.00

295596
1
2

 0.00
3
species
0

1

 0.00
1427984
3
strain

0
order
4
186328
1
8

 0.00

4
family
0
2131
1

 0.00
7

2132

 0.00
1
6
genus
1
4

2
no rank
0
2637901

 0.00
1
3

1
species
3066287
1

 0.00

species
1

 0.00
1
3066326

0
species
1
301585
2
1

 0.00

1
strain
1276218
1

 0.00

order
0
4
5
1

 0.00
186329

1
4

 0.00
2146
family
1
4

33926
1

 0.00
3
2
genus
3

85628
1
2

 0.00
1
species group
0

1
species
1

 0.00
35773

1

 0.00
5
1912503
0
class
1

order
0
1
1
4

 0.00
2975519

1
family
0
3
1

 0.00
3078170

1
0
genus
1
2

 0.00
3078171

species
1
2725268
1

 0.00

2
phylum
0
6
1

 0.00
67819

2
0
class
1042312
1
5

 0.00

2494207

 0.00
1
4
order
0
2

1
3

 0.00
2494208
2
family
0

genus
0
2
2494209
1
2

 0.00

1

 0.00
2219043
2
species

200930

 0.00
1
11
3
0
phylum

68337
10
1

 0.00
class
0
3

191393
1

 0.00
9
order
0
3

0
family
1
2945019
4
1

 0.00

0
genus
1
1
3

 0.00
117999

0
species
1

 0.00
1
2
118000

522772
1

 0.00
1
strain

2945022
4
1

 0.00
2
family
0

0
genus
2
1
3

 0.00
2351

1

 0.00
2
2352
species
0
2

strain
2
717231
1

 0.00

74152
7
1

 0.00
3
phylum
1


 0.00
1
6
641853
class
0
2

2
0
order
641854
1
5

 0.00

641876

 0.00
1
4
2
0
family

1

 0.00
3
423604
2
0
genus

0
species
2
2
1

 0.00
423605

445932
1

 0.00
2
strain

0
superkingdom
15
19
1

 0.00
2157

kingdom
0
1
1783275
1
5

 0.00

651137

 0.00
1
4
0
phylum
1

651142
1

 0.00
3
0
no rank
1

0
genus
1
1593364
1

 0.00
2

1410606
1

 0.00
1
species

3366610
13
14
kingdom

phylum
0
14
28890
1

 0.00
13

183968

 0.00
1
5
0
class
2

order
0
2

 0.00
1
4
2258

2
0
family
2259
1

 0.00
3

2260
1
2

 0.00
0
genus
2

2
species
2261

 0.00
1

1
7

 0.00
2290931
0
clade
12

1
6

 0.00
224756
12
0
class

1
5

 0.00
94695
order
0
12

12
family
0
2206
4
1

 0.00

11
genus
2207

 0.00
1

1

 0.00
2
2220
genus
0
1

1

 0.00
3072978
species
1

10239
116
1

 0.05
1514
0
superkingdom

1514
clade
0
2731341

 0.05
1
115

2731360
1
114

 0.05
kingdom
0
1514

1514
phylum
0

 0.05
1
113
2731618

2731619
1

 0.05
112
1514
0
class

genus
1

 0.00
1
2843470

1
genus
0
3
1

 0.00
2843449

1
0
no rank

 0.00
1
2
2852760

201847
1

 0.00
1
species

1623305
1

 0.03
27
1117
genus
21

0
species
1
1
2

 0.00
2845163

1
no rank

 0.00
1
1874589

2845169
1

 0.00
2
0
species
1

1

 0.00
2029665
no rank
1

2845473
1

 0.00
2
1
0
species

no rank
1
1

 0.00
1874593

2
0
species
2
1

 0.00
2845415

no rank
2

 0.00
1
2027250

2845400
2
1

 0.00
species
0
6

no rank
6
1

 0.00
2675255

2
1

 0.03
2845458
1065
species
0

1636587
1

 0.03
1065
no rank

1
species
0
2845399

 0.00
1
2

no rank
1

 0.00
1
2675254


 0.00
1
2
2845175
2
species
0


 0.00
1
2029668
no rank
2


 0.00
1
2
2845176
0
species
6

6
no rank
1

 0.00
2029669

1

 0.00
2
2845148
species
0
4

no rank
4
2029658
1

 0.00

species
0
4
2845479
1
2

 0.00

no rank
4

 0.00
1
1262538

2
1

 0.00
2845456
species
0
1

1
no rank
1636583
1

 0.00

species
0
2
1

 0.00
2
2845437

1

 0.00
2662298
2
no rank

66
1

 0.01
186532
350
genus
50

2845422

 0.00
1
2
9
0
species

9
no rank

 0.00
1
2608369

1

 0.00
2
2845440
4
species
0

2675265

 0.00
1
4
no rank

12
species
0
2845392

 0.00
1
2

12
no rank
2675244

 0.00
1

species
0
5

 0.00
1
2
2845463

2305480

 0.00
1
no rank
5

2
1

 0.00
2845149
0
species
13

13
no rank
2024334
1

 0.00

36343
1
2

 0.00
31
species
0

1

 0.00
2898540
no rank
31

10
no rank
0
3
1

 0.00
329156

6
species
1

 0.00
1862961

4
species
1862962
1

 0.00

0
species
2
2845172
1
2

 0.00

1

 0.00
2024338
2
no rank

25
species
0
1

 0.00
2
2845470

25
no rank
2305489

 0.00
1

1
2

 0.00
2845423
1
0
species

1
no rank
1862960
1

 0.00

2845388
1

 0.00
2
0
species
5

2675238
1

 0.00
5
no rank

2845393
1

 0.00
2
0
species
24

24
no rank
2675245
1

 0.00

11
species
0
2845173
2
1

 0.00


 0.00
1
2024339
no rank
11

8
0
species
2845165
1

 0.00
2

8
no rank

 0.00
1
2024337

0
species
2
2845467

 0.00
1
2

no rank
2

 0.00
1
2305487

2845403

 0.00
1
2
0
species
1

no rank
1
2675258
1

 0.00

0
species
2
1

 0.00
2
31537

2681624

 0.00
1
no rank
2

0
species
6
2845439

 0.00
1
2

no rank
6

 0.00
1
2675263

1

 0.00
2
2845466
5
species
0

5
no rank

 0.00
1
2305485

2845404

 0.00
1
2
4
0
species


 0.00
1
2675259
4
no rank

2845409
1

 0.00
2
species
0
4

4
no rank
1862959
1

 0.00

1

 0.00
2
2845469
1
species
0

2847818
1

 0.00
no rank
1


 0.00
1
2
2845465
0
species
7

no rank
7
2305484
1

 0.00

1
2

 0.00
2845177
species
0
7

no rank
7
2024340

 0.00
1


 0.00
1
2
2845471
5
species
0

5
no rank
2305483

 0.00
1

1

 0.00
2
2845408
13
species
0

13
no rank
1

 0.00
1862958

34
0
species
2845389
1
2

 0.00

2675240
1

 0.00
34
no rank

4
0
species
2845164
1
2

 0.00

no rank
4
2024336

 0.00
1

2845387
2
1

 0.00
0
species
7

7
no rank
2675237
1

 0.00

12
species
0
2845406
1

 0.00
2

no rank
12
2675260
1

 0.00

2845160
1

 0.00
2
10
species
0

no rank
10
2024335
1

 0.00

2845390
1

 0.00
2
16
species
0


 0.00
1
2675242
no rank
16

2843409
3
1

 0.00
1
0
genus

2845135

 0.00
1
2
1
0
species

146269

 0.00
1
no rank
1

2788787
1
4

 0.00
24
0
no rank

35345

 0.00
1
species
1

22
species

 0.00
1
1871687

1
species
1

 0.00
1572712

17
0
genus
1

 0.00
3
2843351


 0.00
1
2
2845430
17
species
0

no rank
17
1

 0.00
2662295

3
subfamily
0
1

 0.00
4
2169599

genus
2
3
2169622
1

 0.00
3

0
species
1
2170249

 0.00
1
2

1262521

 0.00
1
no rank
1


 0.16
1
30
2759
superkingdom
0
5178

29
1

 0.16
33154
0
clade
5178

33208

 0.16
1
28
5178
0
kingdom

6072
1

 0.16
27
clade
0
5178

26
1

 0.16
33213
clade
0
5178

clade
0
5178
1
25

 0.16
33511

7711
1
24

 0.16
phylum
0
5178

subphylum
0
5178
89593
1
23

 0.16

7742
1

 0.16
22
5178
clade
0

clade
0
5178
7776

 0.16
1
21

117570
1

 0.16
20
5178
clade
0

1
19

 0.16
117571
clade
0
5178


 0.16
1
18
8287
0
superclass
5178

1338369
17
1

 0.16
0
clade
5178

5178
clade
0
16
1

 0.16
32523

0
clade
5178

 0.16
1
15
32524

class
0
5178

 0.16
1
14
40674

5178
clade
0
32525
1
13

 0.16

9347

 0.16
1
12
0
clade
5178

5178
0
clade
1437010
11
1

 0.16

314146
1

 0.16
10
0
superorder
5178

5178
0
order

 0.16
1
9
9443

5178
suborder
0
1
8

 0.16
376913

1
7

 0.16
314293
5178
infraorder
0

5178
parvorder
0
9526
6
1

 0.16

1
5

 0.16
314295
5178
0
superfamily

5178
family
0
9604
1
4

 0.16

207598
3
1

 0.16
5178
subfamily
0

genus
0
5178
9605

 0.16
1
2

species
5178
1

 0.16
9606
