# Supplementary material for: Development and validation of a LAMP assay for detection of genetically diverse Lactococcus c2-type bacteriophages in dairy environments
Source: Front Microbiol. 2026 Aug 10;17:1912103. doi: 10.3389/fmicb.2026.1912103 (PMC13500662; doi:10.3389/fmicb.2026.1912103)
Supplement: Supplementary file 2 [file Data_Sheet_1.ZIP › Supplementary File S1/L.lactis_997_50_krona.html]

Javascript must be enabled to view this page.

magnitude
magnitudeUnassigned

krona

10166190

0
3961

0
3956

3956
44

3
231

183

44

1

20

79
49

1

2

1

1

1

1

3

1

1

3

1

4

3

1

3

1

2

2

634
1462

3

11

40

1

6

6

1

102

9

12

19

17

14

1

9

3

106

3

15

68

2

10

3

6

32

244

29

11

37

8

500

2

88
76

4

8

1
0

1

0
1

1

8

110

74

2
0

1

1

0
1

1

1
0

1

40

0
1

1

123
79

2

18

24

59
141

2

1

6

1

1

4

15

1

3

2

1

18

5

1

1

1

9

1

1

2

6

891
211

53
46

1

2

2

1

1

606
503

1

1

1

1

4

2

2

1

1

2

1

3

3

4

1

11

5

1

2

5

3

1

1

3

4

6

2

7

11

5

1

4

2

1

21
19

1

1

96
97

1

27

1

4

0
5

5

2
0

2
0

2
0

0
2

0
2

2

3
0

0
3

0
3

3
0

0
1

1

2
0

2

99355
9259959

1
0

0
1

1
0

1
0

1

0
121

2
0

2
0

0
2

2
0

2

16
0

16
0

0
16

0
16

15

1

21
0

21
0

21
0

21
0

21

0
72

0
72

0
72

1
72

71

0
9

9
0

0
9

9

1
0

0
1

0
1

0
1

1

0
3

0
3

0
3

3
1

0
1

1

1
0

1

0
71

71
0

0
71

2
42

14
40

3

2

1

3

1

6

10

29
0

29
6

1

1

10

2

5

1

1

2

1
0

0
1

1
0

0
1

1
0

1

0
1352

1352
1

1256
0

1256
0

1256
255

490

1

3

1

12

1

4

1

3

2

6

231

73

1

5

109

1

1

22

1

23

9

1

0
95

0
95

49
89

2

3

4

5

1

2

23

4
0

4

0
1

1

1

1861
9098998

0
2

0
2

1
2

1

37
0

37
0

6
25

0
1

1

2
0

2

3
0

3

1
0

1

12
1

1

9

1

0
11

0
7

4

3

2

2
0

2

0
1

1

0
162

154
0

154
0

13
153

99

25

5

6

4

1

0
1

1

0
8

8
0

4
7

2

1

0
1

1

5
557

505
0

494
12

87
18

2

17

12

24

3

11

0
1

1

85
3

22

60

2
0

2

5
0

5

0
302

302

1
0

0
1

1

7
0

0
7

7

0
3

2
0

2

1
0

1

0
47

4

43

26533
9096028

3142
53

23
544

9
0

1

6

1

1

9

1
0

1

1
88

2

1

84

1
0

1

0
7

7

200
27

11

2

3

1

1

3

3

24

10

14

10

4

20

4

1

16

12

1

10

1

1

20

1

37
33

1

1

1

1

1
0

1

0
1

1

31
17

12

1

1

0
2

2

17
0

2

1

1

3

1

1

1

1

1

5

1
0

1

0
2

2

0
4

3

1

3
0

2

1

0
6

3

3

8
5

1

1

1

0
1

1

0
2

2

1
2

1

1
78

1

1

2

73

1
0

1

2

0
1

1

0
2

1

1

0
3

1

1

1

1
0

1

141
7

128
10

2

1

1

1

2

27

1

2

1

8

1

1

1

2

5

60

1

1

1
0

1

5
0

1

4

5
0

0
2

2

2
0

2

1
0

1

69
0

69
1

3

44

21

37
1

1

7
0

6

1

2
0

2

1
15

1

2

1

1

7

2

0
1

1

8
0

1

1

1

2

2

1

2
1

1

2164
34

0
1

1

0
19

16

3

0
8

1

7

26
0

24

1

1

1
0

1

185
2048

264

1

9

2

4

42

475

669

14

1

1

10

1

4

2

1

5

2

11

4

1

15

8

143

85

1

5

3

16

54

5

3

2

27
0

27

0
72

13
0

13

9
59

4

30

2

12

2

0
6

6
0

6

17
50

2

1

1

9

1

2

10

1

6

1
0

0
1

1

454451
9066353

3058
17665

0
1

1

225
24

1

10

1

30

51

91

2

14

1

1
0

1

8
4

1

2

1

117
2061

26

1

1

1

644

1

5

1

1

7

97

1

1

1147

4

5

1

79
0

6

2

2

1

22

1

44

1

35
528

453

2

20

18

1
0

1

805
1

761

37

2

1

3

18
0

2

1

6

8

1

10
12

1

1

0
72

4

8

1

59

0
6

6

11

144
39

31

74

2
123

23

25

67

6

1
0

1

50
0

6

44

48
0

1

43

4

3686
9637

465

56

155

2

7

1

4972

64

1

135

81

12

4
0

4

25
6

2

1

3

1

1

11

0
3

2

1

731
32

54

2

640

2

1

0
13

9

4

32
3390

382
3145

5

5

7

9

26

7

2

1473

2

6

1

870

249

1

11

1

1

1

2

8

76

7
69

55

7

1
144

3

43

1

2

12

5

1

68

6

2

15
184

2
0

2

0
23

23

0
5

5

0
1

1

1
0

1

0
3

3

120
19

9

12

2

7

71

0
14

14

165
0

7
52

3

7

30

2

3

0
7

2

4

1

0
3

1

1

1

36
0

36

32
0

29

3

0
3

3

10
32

3

19

8590498
216443

4
0

4

3963703
8359816

1068

409

98

3375438

38

1923

345903

1015

3401

99

142

663740

52

17

2629

140

1

1783
14235

119

54

25

3

4

2

4

22

12

10

2

2

18

2

2

47

2

64

2

2

3

65

2

8

15

8

1

2

212

3257

2

1

61

116

44

3

6

156

60

4

22

2

69

1

6751

9

124

15

4

24

4

5

1

2

38

6

3

49

1

63

20

15

20

243

2

1

11

173

42

4

171

17

12

13

3

3

85

7
351

3
0

3
0

2
0

1

1

1
0

1

45
0

1
0

1
0

1

2
0

0
2

2

3
42

1
0

1

0
2

2

6
0

6

1
0

1

5
0

2

3

3
0

3

0
1

1

0
1

1

2
1

1

1
0

1

10
0

10

0
2

1

1

2
0

2

2
0

2

60
0

2
0

0
2

2

13
0

13
0

13

10
0

0
9

1

8

0
1

1

2
35

0
3

3

0
1

1

27
0

27

0
1

1

1
0

1

8
218

3
0

3
1

1

1

1
0

0
1

1

8
3

0
4

4

0
1

1

29
2

0
4

4

7
6

1

0
1

1

5
0

5

1
5

2

1

1

0
3

3

2
0

2

3
0

3
0

3

0
1

1

22
0

21

1

0
1

1
0

1

137
0

1
0

1

18
132

5

1

3

4

1

76

1

1

1

4

5

1

2

7

1

1

1

1
0

1

2
0

2

5
0

5
0

5

1
0

0
1

1
0

1

4
0

1
0

1
0

1

0
3

0
3

3

5
0

5
0

4
0

4

0
1

1

6
0

0
5

5

1
0

1
0

1

0
2

2
0

0
2

2

1630
21

0
1016

1016
11

7
155

0
1

1

1
2

1

1
0

1

0
3

3

0
1

1

6
2

1

1

1

1

1
0

1

3
0

3

2
4

1

1

14
4

3

3

2

1

1

31
92

3

1

1

1

3

1

1

1

1

2

1

1

2

1

1

1

1

2

1

1

4

4

4

1

1

1

1

1

1

1

7

1

1

1

1

1

1

1

1

0
1

1

0
3

1

1

1

0
5

2

3

2
1

1

0
1

1

1
0

1

1

0
1

1

2
0

2

1
2

1

0
1

1

0
1

1

76
821

0
1

1

2
68

2

1

54

1

4

3

1

4
0

3

1

0
97

92

5

1
0

1

0
2

2

1
12

10

1

134
472

222

82

34

87
17

11

2

2

1

4

1

2

1

1

1

1

2

1

1

1

3

2

3

4

1

12

1

1

2

1

2

5

1

0
1

1

28
0

0
28

28

0
114

3
114

84
7

3
0

2

1

13
59

1

1

3

4

4

1

3

3

1

1

2

6

1

1

2

2

3

1

1

2

3

14
0

13

1

0
1

1

14
0

3
0

3

0
3

2

1

0
6

6

2
0

2

5
0

3
5

1

1

1
0

1

1
0

0
1

1

0
1

0
1

1

5
0

5
1

1

1

1

1

0
413

410
24

5
0

0
5

1

1

3

42
0

3
42

5

9

3

18

4

1
18

7
0

2

5

10
1

1

1

2

1

1

3

0
2

2
0

2

2
311

35
0

2

33

30
225

16

20

1

42

3

53

13

17

7

3

17

3

49
2

12

35

7
0

7
0

6

1

0
1

1
0

1

3
0

0
3

2
0

2

0
1

1

0
26

26
0

26
0

1
0

1

0
2

2

1
0

1

0
1

1

1
0

1

3
0

3

1
0

1

1
0

1

0
1

1

8
10

1

1

2
0

2

2
0

1

1

0
37

0
37

2
37

19
3

1

1

1

1

3

1

2

6

10
2

1

2

4

1

0
6

1

1

1

3

0
3

0
3

2
0

1
0

1

0
1

1

1
0

0
1

1

2
0

2
0

2
0

0
2

2
0

2

28
40045

0
133

130
0

0
106

0
2

1

1

99
0

99

0
5

1

4

0
24

0
23

23

1
0

1

0
3

3
1

2
0

1

1

2
22

18
3

3
0

1
3

1

1

6
0

0
6

1

2

3

5
0

0
5

5

1
0

1
0

1

2
0

0
2

2
0

2

0
1

1
0

0
1

0
1

1

0
1

0
1

1
0

1
0

1

19
0

19
5

0
13

0
7

7

2
0

2

3
1

1

1

1
0

1

0
1

1
0

1

394
39841

1
0

1
0

1
0

1

219
0

3
219

207
55

1

1

1

1

1

1

1

1

1

1

2

1

1

2

3

2

1

2

1

1

3

1

1

1

1

2

2

1

1

1

1

1

1

4

1

1

1

1

1

1

7

2

2

1

4

1

1

1

1

1

1

4

2

1

1

1

1

1

1

1

1

1

1

1

1

1

1

2

1

1

1

1

23

1

1

1

2

1

1

1

5

4

2

2

4

1

1

1

1
8

2

1

1

1

1

1

0
1

1

0
1138

1138
3

98
32

16

1

1

9

32

1

1

1

4

151
1037

11

89

575

211

0
5

0
5

0
4

2

2

0
1

1

0
190

3
190

0
1

1

109
8

1

1

1

89

1

1

2

1

1

2

1

2
1

1

5
0

2

1

1

1

1
0

1

0
2

2

3
0

3

5
0

5

0
3

1

1

1

0
1

1

4
31

24

1

2

1
0

1

5
22

1

2

5

5

2

1

1

0
1

1

10
0

0
10

0
9

3

1

2

1

2

1
0

1

0
2

2
0

2
0

2

0
9

0
9

9
0

2

4

1

2

9
0

0
9

0
9

3

6

54
3293

0
124

0
1

1

15
123

2

1

1

1

27

3

2

7

1

57

2

1

1

2

2
12

2
0

2

1
0

1

2
6

1

1

1

1

0
1

1

104
5

0
5

5

1
0

1

0
5

5

0
1

1

25
81

4

16

1

2

24

9

6
0

6

3
0

3
0

3

64
2

43
1

1

5

1

35

19
3

1

2

3

2

5

2

1

112
3

80
28

2

1

2

1

2

7

21

1

1

14

6
29

4

14

5

0
38

0
3

3

4
0

4

31
0

27

4

44
417

0
2

1

1

2
10

1

6

1

0
3

1

1

1

0
1

1

7
2

1

1

1

1

1

1
0

1

7
0

1

6

246
113

1

1

3

1

1

2

1

1

3

1

2

12

1

2

1

12

3

1

2

5

1

1

1

1

1

5

3

1

1

1

1

2

1

3

1

1

1

1

6

3

2

1

1

1

2

1

1

4

1

1

2

10

1

4

1

1

2

2

2

1

2
1

1

19
2

1

2

6

5

3

2
5

1

1

1

2

0
9

4

1

2

2

0
2

2

1
3

1

1

5
10

1

1

1

1

1

2
0

1

1

3
0

3

1
0

1

9
0

9

0
1

1

0
3

3

1
0

1

2
0

1

1

2
0

2

2
20

1

1

1

10

1

1

2

1

5
0

0
5

1

2

2

0
4

0
1

1

3
0

3

0
3

3
0

1

2

34
0

34
0

34

2
0

2

0
4

3
0

2

1

0
1

1

38
2293

1
0

1

3
0

3

1
2

1

190
1

73

8

9

35

19

45

1536
1097

8

20

33

16

28

280

10

10

2

29

2

1

0
4

3

1

0
1

1

4
0

2

2

0
1

1

18
453

58

6

2

4

256

1

2

106

9
1

1

1

1

3

2

3
48

3

1

2

2

9

1

2

13

1

1

2

2

1

1

1

1

1

1

0
3

3

0
18

1
0

1

0
2

2

15
0

15

2
0

0
2

1

1

0
418

418
1

0
5

5

3
103

15

64

1

9

11

2
1

1

1
0

1

0
3

2

1

0
3

1

1

1

0
4

4

0
60

60

2
0

2

3
0

1

2

1
0

1

79
230

15

6

1

2

1

5

7

66

1

1

19

2

6

11

2

4

2

0
53

9
53

2
7

4

1

2
19

9

8

18
5

2

5

2

4

14
29675

0
3

3
2

1

3
278

45
254

4

4

5

3

3

5

2

1

5

2

3

4

3

1

4

2

1

1

2

1

3

61

1

9

18

2

1

2

4

4

1

2

7

5

9

3

3

1

2

3

2

1

9

2
0

2

3
8

1

2

1

1

2
0

2

0
9

9

10
29380

2
0

2

797
29310

3

27753

492

34

231

20
1

1

8

10

11
1

5

5

1
5

3

1

0
1

1

3
0

3

0
4

2

2

12
3

3

1

4

1

0
2

2

29
4343

0
377

46
377

1

3

1

1

3

2

2

3

1

60

4

204

1

19

1

2

23

0
3331

592
3331

3

127

7

1

37

3

1

7

4

1

1

2

24

25

5

6

1

144

52

14

9

2

1

2

2

1

1

246

8

1

47

26

11

3

1

3

7

18

1

9

35

5

86

2

2

141

7

4

1

9

3

43

1

1

6

7

34

7

2

3

22

1

794

1

248

2

26

6

118

1

1

1

1

34

3

1

5

10

62

1

1

3

5

1

22

1

18

1

1

1

3

2

79

295
35

4
0

1

1

2

1
0

1

138
55

1

1

1

1

1

3

2

1

1

1

2

1

1

2

2

1

1

1

2

1

1

22

10

5

1

1

1

3

1

7

2

1

1

16
117

1

1

1

2

1

3

2

1

1

7

1

4

1

1

1

2

2

6

2

2

1

1

42

6

1

1

1

1

3

1

1

3
0

0
3

2

1

238
24

87
0

84

3

16
4

2

2

1

1

2

1

1

1

1

25
111

5

2

1

1

2

1

1

3

1

65

3

1

23
0

0
23

23

0
47

47
11

9

4

3

3

2

15

0
53

53
0

1
0

1

1
0

1

1
0

1

0
1

1

0
4

4

1

1
0

1

0
2

2

9
1

1

1

2

1

2

1

32
10

1

2

1

1

1

2

2

2

1

1

1

1

2

1

1

1

1

29
2

2
12

1
2

1

2
0

1

1

0
1

1

1
5

1

2

1

0
10

9
2

1

2

2

1

1

0
1

1

5
0

1
0

1

3
2

1

1
0

1

9
0

0
4

4
0

4
0

4
0

2

2

5
0

2
0

2
0

0
2

2

3
0

1
3

0
2

1

1

22
2

6
0

0
4

1
0

1

0
3

3

0
1

1
0

1

0
1

0
1

1

1
0

1
0

1
0

1

13
0

13
0

0
7

3
0

2

1

0
4

2

2

1
0

1
0

1

0
5

0
2

2

0
1

1

1
2

1

0
24

1
0

0
1

0
1

1
0

1

5
0

4

0
1

0
1

1
0

1

3
17

2
0

0
2

1
0

1

1
0

1

0
1

1
0

1
0

1

0
5

5
0

1

0
1

1

2
0

2

0
1

1

0
6

0
6

1
0

1

5
0

5

0
1

1
0

1
0

1
0

1

7
69

39
0

0
2

0
2

2
0

1

1

0
37

0
37

0
37

37

4
0

0
4

0
4

1
0

1

2
0

1

1

0
1

1

5
0

0
5

0
4

4
0

4

0
1

1

0
3

2
3

0
1

1
0

1

10
0

0
10

0
6

0
1

1

0
5

5

0
4

0
3

3

0
1

1

1
0

1
0

1
0

0
1

1

16960
233

0
23

23
0

23
0

0
23

23

1
0

1
0

1
0

0
1

1

3665
171

0
600

600
7

5
13

2

4

1

1

2
0

2

552
157

6

16

1

9

15

1

2

44

5

46

5

9

4

4

3

26

7

1

157

1

7

1

3

5

1

9

3

2

2

26
4

1

12

1

1

3

1

2

1

155
1

32
1

3
6

1

1

1

1
0

1

5
0

5

19
6

2

1

2

1

2

3

1

1

0
3

3
0

3

0
3

0
3

3

7
105

1

0
1

1

41
79

29

3

2

4

0
3

3

2
0

2

7
2

2

1

2

2
0

2

1
0

1

0
1

1

1
0

1

3
0

2
3

1

8
0

4
0

2

2

0
2

1

1

0
2

2

0
3

0
3

0
3

1

1

1

570
26

515
14

0
10

10

2
0

1

1

1
0

1

0
2

1

1

1
0

1

2
0

2

1
0

1

1
0

1

4
1

2

1

1
0

1

4
0

1

3

0
5

1

4

1
0

1

1
0

1

1
0

1

0
1

1

0
1

1

138
461

1

2

3

2

44

6

40

2

10

2

2

19

19

3

2

3

3

9

1

2

17

1

1

9

2

89

22

6

1

0
1

1

2
29

1
0

1

0
1

1

1
0

1

1
0

1

1
2

1

1
0

1

3
1

2

1
4

1

1

1

0
1

1

1
0

1

0
1

1

1
0

1

5
0

5

0
1

1

3
0

2

1

772
40

0
7

0
4

1

1

2

3
0

3

63
654

0
1

1

0
5

5

192
71

71

2

1

2

3

1

3

3

3

4

7

5

2

3

1

6

2

1

1

3
0

2

1

3
0

3

278
66

10

2

4

2

3

3

1

4

2

6

7

1

1

1

1

1

8

1

1

1

1

1

4

2

5

1

5

1

1

3

4

2

7

1

2

2

8

3

1

3

4

5

1

2

1

8

2

3

4

1

1

13

2

5

38

3

1

1

33
6

1

1

2

1

1

1

1

7

4

4

2

1

1

2
5

2

1

0
1

1

0
1

1

0
1

1

0
4

2

1

1

62
4

14

3

6

2

3

2

1

2

2

12

1

3

1

1

4

1

2
0

2

13
71

1
16

5

6

1

2

1

0
2

1

1

1
0

1

0
5

1

4

0
4

2

1

1

9
0

1

1

1

6

3
0

2

1

2
18

1

2

1

1

1

1

2

6

1

1
0

1
0

1
0

1

2
0

2
0

0
1

1

1
0

1

0
1

0
1

1

8
1

0
4

0
1

1

3
2

1

3
0

1
0

1

1
2

1

65
1381

0
1

1
0

1

34
1

0
1

1

0
1

1

27
7

1

2

1

2

1

3

1

2

2

2

2

1

4
0

3

1

30
0

30
13

1

2

1

2

5

2

1

1

2

0
1

0
1

1

3
0

2

0
1

1

0
5

0
5

5

1
0

1
0

1

0
1

1

0
2

2
0

2

6
0

0
2

2

1
0

1

3
2

1

130
24

1
0

1

5
20

1

8

1

1

4

6
2

1

1

1

1

2
1

1

13
4

3

2

3

1

1
4

1

1

1

1
17

3

1

12

0
2

2

0
2

1

1

39
12

1

7

1

1

1

4

1

2

3

1

1

1

1

2

1
20

1
0

1

18
3

1

1

1

4

2

3

1

1

1

644
35

64
16

15

14

19

5
0

3

1

1

540
328

4

4

3

117

1

1

4

1

7

1

1

8

6

6

3

7

1

4

3

1

5

17

7

0
9

1
6

4

1

1
2

1

0
1

1

406
10

198
380

1

7

2

1

6

3

5

3

4

3

1

4

2

1

2

2

2

3

7

11

1

1

3

1

1

1

2

1

2

2

1

9

2

1

2

3

1

1

2

1

3

3

2

1

1

8

12

14

1

26

2

1

1
0

1

0
2

1

1

12
1

4

2

5

0
1

1

1
0

1
0

1

0
2

0
2

2

0
1

1
0

1

1
0

1
0

1

1
0

1
0

1

0
4

1
0

1

2
0

1

1

1
0

1

0
2

0
1

1

1
0

1

0
3

3

8
1

0
5

5

0
2

1

1

0
1

0
1

0
1

1

23
2413

184
2

0
4

1
0

1

0
3

1

1

1

0
170

1
0

1

169
62

54

1

52

8
1

0
3

1

2

4
1

1

1

1

0
89

1
0

1
0

1

2
0

1
0

1

0
1

1

6
0

0
1

1

0
1

1

1

0
2

1

1

1
0

1

80
6

0
1

1

0
1

1

4
0

4

2
0

1

1

65
29

1

1

8

1

1

3

1

9

11

1
0

1

1
0

0
1

1

2081
104

0
3

3

5
0

3

2

220
12

0
3

3

0
8

8

0
1

1

10
0

4

6

71
18

4

12

25

1

5

3

3

19
5

1

13

2
0

2

1
0

1

44
0

44

49
0

43

4

2

62
548

5
29

3

2

2

2

3

4

3

2

1

2

1
0

1

0
1

1

4
0

1

3

87
62

1

2

1

1

1

1

4

2

2

9

1

5
0

5

5
0

5

6
0

3

1

2

7
0

2

4

1

12
102

3

1

3

9

1

9

1

3

57

2

1

2
10

4

2

2

115
96

11

5

1

2

4
0

1

3

0
1

1

11
96

3

2

1

1

2

3

59

1

7

5

1

12
5

4

3

1
0

1

2
0

2

28
915

187
737

6

15

58

5

6

1

399

2

58

15
4

2

3

2

1

1

1

1

21
0

21

1
3

1

1

45
9

7

1

1

3

2

3

2

10

4

1

1

1

7
2

2

1

2

1
5

3

1

0
8

8

12
46

2

7

3

1

2

1

1

17

247
29

2
0

2

31
143

1

1

4

35

6

2

3

38

1

4

15

2

9
0

1

2

1

1

1

3

0
2

2

0
22

22

4
0

1

2

1

3
0

1

2

32
9

7

6

4

1

1

2

2

1
0

1

0
2

2

35
1

0
1

1

2
0

2

4
14

1

1

3

1

1

3

7
1

1

1

1

1

2

0
8

3

3

2

0
1

1

1
0

1

1
35

0
1

0
1

1

0
11

0
10

10

1
0

1

0
17

0
1

1

0
16

16

2
0

0
1

1

0
1

1

2
0

2
0

2

1
0

1
0

1

135
10625

137
0

137
5

1

0
1

1

1
0

1

3
0

3

1
0

1

3
13

1

5

3

1

112
6

81

2

1

1

4

11

6

0
5

0
5

1
0

1

0
4

1

1

2

0
6

6
0

2
0

1

1

4
2

2

2

334
4

317
21

58
8

2

36

9

1

1

1

67
21

1

1

1

1

1

1

9

1

30

35
98

4

18

1

6

1

1

7

1

1

9

2

4

8

6
12

2

1

1

2

8
1

1

1

3

2

53
45

1

1

3

2

1

2
13

0
2

2

0
1

1

0
2

2

0
3

1

2

0
3

1

2

0
10

5
0

4
0

4

1
0

1

2
0

0
2

2

0
3

3
0

1

1

1

4025
0

22
4025

2071
710

1216

41

2

55

47

67
28

2

1

1

7

1

3

1

1

8

1

1

6

1

5

943
1865

5

1

1

2

1

30

1

1

1

1

2

1

2

2

4

4

1

31

1

1

1

4

40

219

2

1

7

18

1

4

1

2

1

12

1

1

3

4

1

2

4

3

17

1

42

10

1

2

1

8

355

2

1

1

3

1

50

2

0
3164

2
0

2
0

2

3162
72

16
60

1

1

42

3022
1795

4

3

16

2

1

9

2

7

14

2

4

1

1

1

15

2

3

7

23

18

3

45

1

1

1

1

1

3

1

1

3

2

1

2

1

2

1

1

11

22

2

1

8

1

15

1

1

1

1

2

1

1

5

1

170

1

2

2

2

1

1

18

1

1

3

2

3

31

3

3

1

18

2

4

2

1

1

1

3

2

1

1

1

1

12

2

3

1

1

1

22

1

2

2

1

1

1

1

2

1

1

1

4

1

1

53

2

3

301

1

1

5

4

1

1

3

2

1

23

1

10

2

1

2

1

3

1

2

2

2

1

1

2

3

60

1

3

1

3

2

1

15

1

31

4

1

1

2

2

2

3

1

1

1

1

1

1

1

27

1
7

1

4

1

1
0

1

29
0

0
29

0
27

27

2
0

2

201
3

0
2

2
0

2

34
184

1
0

1

34
117

1

4

1

10

6

6

8

2

1

6

1

9

1

2

1

3

1

3

1

2

3

11

2
27

23

2

2
1

1

0
1

1

2
0

2

9
0

1

1
7

1

4

1

1
0

1

0
3

1
0

1

1
2

1

0
2

2

3
0

0
3

3
0

3

54
0

0
50

39
4

14

21

0
9

9

1
0

1

1
0

1

0
1

1
0

1

0
3

1
0

1

0
2

2

2
0

2
0

2
0

2

48
0

0
48

47
24

1

2

6

14

1
0

1

0
57

57
0

48
5

2

1

4

1

2

6

1

21

1

2

2

1
0

1

0
1

1

7
0

6

1

0
9

5
0

5
0

1

1

1

2

0
4

0
2

2

1
0

1

1

65
2348

0
51

0
1

1

1
3

1

1

0
3

3

40
11

4

2

1

2

1

1

4

13

1

2

0
2

1

1

1070
2

98
0

98

955
8

1

913

1

5

27

13
6

3

1

2

1

0
2

2

0
6

2
0

2

1

3
1

2

36
0

4
5

1

1
2

1

0
20

20

0
9

9

165
906

1
0

1

0
1

1

6
7

1

1
4

3

12
1

11

114
131

1

6

2

2

3

3

1

0
72

72

391
347

1

1

42

1
0

1

0
2

2

1
0

1

1
19

1

17

39
24

2

1

2

2

1

3

4

4

55
4

2

3

41

5

14
0

1
11

1

9

1
0

1

1

1
0

1

0
200

200
0

200

0
54

34
0

14
34

9

1

1

1

1

2

1

1

1

1

1

8
0

0
1

1

1
0

1

6
0

5

1

9
0

9
4

4

1

3
0

1
3

2

0
1

1
0

1
0

1
0

0
1

1

2
8

0
6

6
0

0
6

6
2

2

1

1

1
183

1

37
181

25
0

6
0

0
5

1

4

1
0

1

2
0

1

0
1

1

0
17

13
17

4

30
0

0
30

0
30

30

3
0

0
1

0
1

1

0
2

2
0

2

0
1

1
0

1
0

1

49
6

0
6

0
2

2

1
0

1

2
0

1

1

1
0

1

1
0

1

0
7

0
7

2

5

0
3

2
3

1

0
1

1
0

1

25
1

0
1

1

22
9

2

3

1

1

1

2

2

1

1
0

1

0
2

2
0

2
0

2

1
8

0
3

1
3

2

0
3

1
3

1

1

0
1

1
0

1

0
7

0
7

0
6

2

2

1

1

1
0

1

1
0

0
1

0
1

1

10
0

10
0

10
6

2

2

8
0

3
0

1
0

1

2
0

2

2
0

0
2

2

3
0

0
1

1

0
2

1

1

8
0

8
0

8
0

8
0

8
0

8

0
4

0
4

0
4

0
4

4
0

4

0
7

0
7

0
7

0
1

0
1

1

3
6

0
3

3

0
7

7
0

6
0

6
0

0
1

1

5
0

2

3

1
0

1

4
0

0
4

4
0

4
0

0
4

4

8
0

0
8

8
0

0
8

8

0
20

0
20

0
1

0
1

0
1

1

0
2

2
0

2
0

1

1

1
17

2
0

2
0

1

1

8
0

1
8

1

1

1

1

1

1

1

0
4

3
4

1

2
0

0
1

1

0
1

1

0
47

47
0

47
0

28
0

28
0

2

1

12

1

1

9

2

12
0

0
2

1

1

0
2

1

1

6
0

4

2

0
2

2

7
0

7

0
1

0
1

1
0

1
0

0
1

1

980
0

947
0

0
943

7
0

5

2

0
936

1

1

934

4
0

1
0

1

0
3

2

1

0
1

0
1

1
0

0
1

1

0
32

0
4

4

0
21

0
13

3
13

6

1

1

1

1

8
0

8
0

6

2

0
7

0
4

4
0

1

3

3
0

0
3

3

0
1

1
0

0
1

1
0

1
0

1

4
0

0
3

0
3

3
1

2
0

2

0
1

0
1

0
1

0
1

1

13
0

6
0

0
6

6
0

6
1

3

2

5
0

0
5

1
0

0
1

1

0
4

4
0

4

2
0

2
0

1
0

1
0

1

0
1

1

810805

465
91423

2
0

2
0

0
2

0
2

2

6
1603

1584
0

0
1584

1584
0

5
1584

8

1571

0
4

4
0

4
0

0
4

3

1

2
0

2
0

2
0

0
1

1

0
1

1

7
0

3
5

0
2

2
0

2

2
0

2
0

0
2

2

7
209

0
1

0
1

1
0

1

2
31

1
20

0
6

3
6

2

1

9
0

9
0

8

1

3
0

0
1

1

1
0

1

1
0

1

1
0

1
0

1

1
8

1
0

1
0

1

5
0

5
3

2

1

1
0

0
1

1

1
85

84
2

1
0

1
0

1

3
81

5
0

1

4

9
73

42

5

4

10

3

7
0

7
0

7
0

0
2

2

4
0

4

1
0

1

1
0

1
0

0
1

0
1

1

0
56

56
0

56
0

0
4

3

1

32
0

32

0
18

18

1
0

1

0
1

1

8
0

8
0

8
0

8
0

8

13
0

0
6

6
3

2
0

2

0
1

1

0
7

0
4

4
0

4

3
0

3
0

3

2
0

2
0

2
0

0
2

2

0
1

1
0

1
0

1

0
89036

0
89036

89036
0

0
89036

0
89036

89036

0
1

0
1

1
0

1

81
0

1
0

0
1

0
1

0
1

1

80
0

68
0

17
0

17
0

17

51
0

0
51

51

0
12

12
0

3
12

1

1

2

5

11
0

11
0

11
0

0
11

0
11

11

0
12

0
12

0
12

0
12

1
12

11

0
42

36
0

0
28

28
2

0
1

1

0
4

0
1

1

0
3

1

2

0
14

0
10

10

1

0
1

1

1
0

1

0
1

1

0
1

1
0

1

4
0

1
2

1

1
0

1

1
0

1

2
0

0
1

1

1
0

1

1
0

1
0

1
0

1
0

1

2
0

0
2

0
2

2
0

2

2
0

1
0

1
0

1
0

1

1
0

0
1

1
0

1

0
3

0
3

3
0

0
2

1

1

1
0

1

0
1

0
1

1
0

0
1

1
0

1

0
4

1
0

1

0
3

3
0

0
3

3
0

1

1

1

1
0

1
0

1
